# Synthesis of 1,3-Diaminoisoquinoline Derivatives via *N*‑Oxide Intermediates from *o*‑Cyanobenzyl Cyanides

**DOI:** 10.1021/acs.joc.6c00531

**Published:** 2026-07-10

**Authors:** Daisuke Sakamoto, Alessandro Prescimone, Philippe H. M. Marliere, Konrad Tiefenbacher

**Affiliations:** † Department of Chemistry, 27209 University of Basel, Mattenstrasse 22, CH-4058 Basel, Switzerland; ‡ Department of Biosystems Science and Engineering, ETH Zurich, Klingelbergstrasse 48, 4056 Basel, Switzerland; § The European Syndicate of Synthetic Scientists and Industrialists (TESSSI), 81 rue Réaumur, 75002 Paris, France

## Abstract

1,3-Diaminoisoquinolines represent distinctive scaffolds that merge the isoquinoline core, prevalent in natural products and pharmaceuticals, with a 2,6-diaminopyridine-type hydrogen-bonding motif. Despite their attractive structural features, access to these frameworks remains limited by existing synthetic methods. Herein, we introduce a facile synthetic method for the preparation of 1,3-diaminoisoquinolines via cyclization of *o*-cyanobenzyl cyanides with hydroxylamine, followed by a one-pot reduction using B_2_pin_2_. This approach exhibits broad substrate tolerance and enables rapid, efficient access to diverse 1,3-diaminoisoquinoline derivatives.

## Graphical abstract

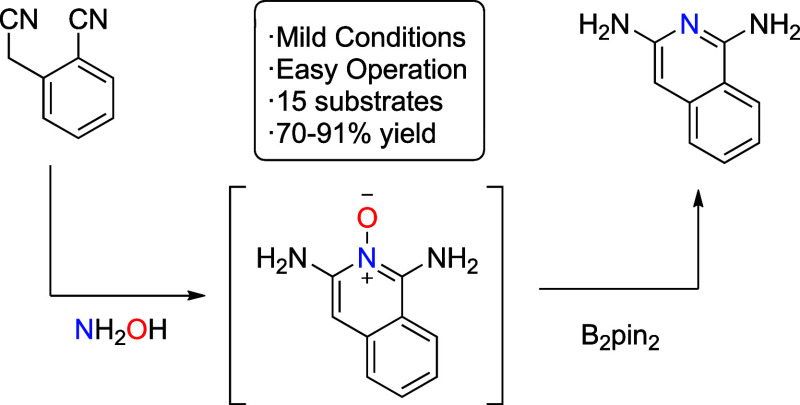

Isoquinoline derivatives are prevalent in natural products and pharmaceuticals, with papaverine used clinically as a nonspecific smooth-muscle relaxant and vasodilator.^1^ The isoquinoline framework, accordingly, is of significant pharmacological interest. In addition, 2,6-diaminopyridine moieties act as donor–acceptor–donor (DAD) hydrogen-bonding motifs that enable molecular recognition and drive supramolecular assembly.^2–5^ They also appear as PNP pincer ligands^6–8^ and as foldamer elements,^9,10^ illustrating their wide utility. Despite the potential of combining these two features in a single scaffold, the synthesis of 1,3-diaminoisoquinoline and its derivatives remains underexplored. Metal-catalyzed cyclizations employing Cu,^11^ Au,^12^ Rh,^13^ or dual catalyst of Cu/Zn^14^ have been described, alongside metal-free approaches such as POCl_3_-initiated cyclizations of amide/urea substrates^15,16^ and ring closures of *o*-cyanobenzyl cyanide derivatives with secondary amines to afford 1,3-diaminoisoquinoline derivatives.^17^ Given their limited synthetic accessibility, reports on the applications of 1,3-diaminoisoquinoline derivatives are few and mainly concern bioactive scaffolds^18^ or coordination ligands,^19^ with biological uses.^20^

To date, the only reported synthesis of the unprotected and unsubstituted parent 1,3-diaminoisoquinoline was disclosed by Elvidge et al. in 1964 and involves the reaction of *o*-cyanobenzyl cyanide with ammonia at 140 °C for 24 h (Scheme 1a).^21^ Although effective, this method employs harsh conditions and reagents that are difficult to handle, and has not been widely explored since. Thus, we developed a modified approach that overcomes this severe limitation and report the details here.

**Scheme 1:**
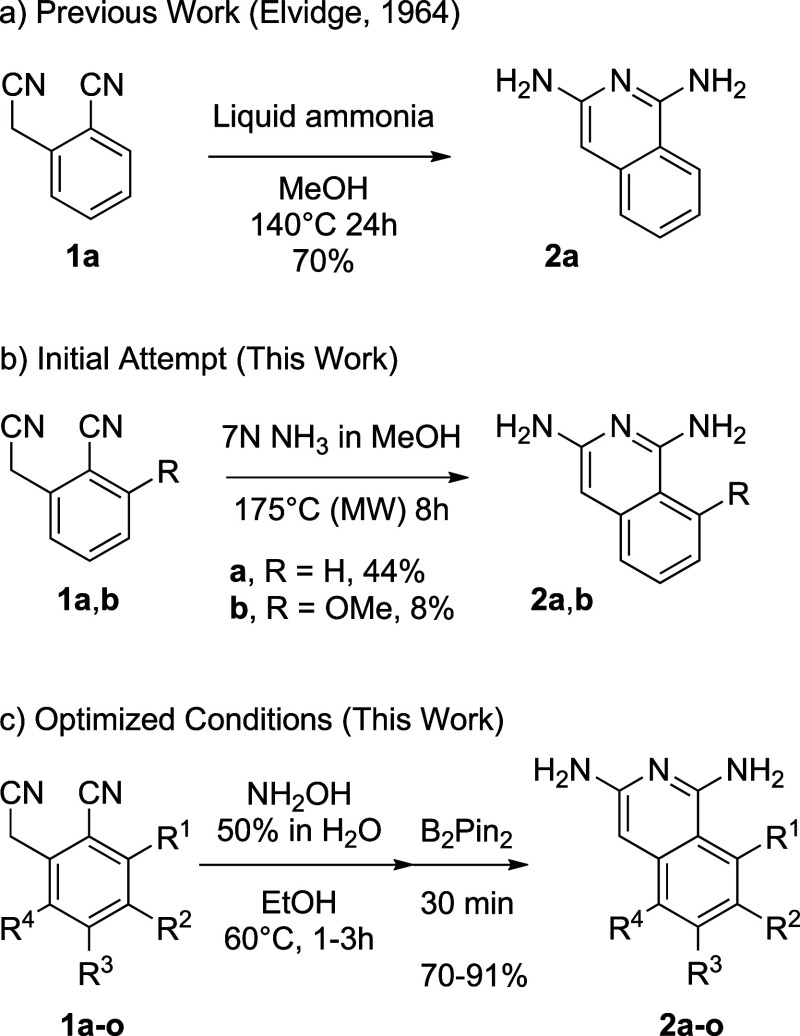
(a–c) 1,3-Diaminoisoquinoline Formation

At the outset of this study, we tried to optimize the reported procedure by Elvidge utilizing ammonia. To improve the practicality of the method, we initially used a commercially available 7 N ammonia in methanol solution instead of liquid ammonia. The mixture of *o*-cyanobenzyl cyanide and the ammonia solution in methanol was subjected to microwave heating at 175 °C for 8 h, yielding 1,3-diaminoisoquinoline (44%, Scheme 1b) along with byproducts, while a small amount of starting material (13%) remained unreacted. Performing the reaction is easier than the reported method, but the conditions to give the desired product are still harsh, and the generation of the undesired compounds decreases yield.

More importantly, the reaction of the 6-methoxy derivative **1b** resulted in only an 8% yield, likely due to the deactivating, electron-donating effect of the substituent.

We therefore turned to hydroxylamine as a more nucleophilic nitrogen source. Its use with *o*-cyanobenzyl cyanide was first reported in 1889,^22^ and the product was later assigned as 1-amino-3-hydroxyaminoisoquinoline **3** (Scheme 2) on the basis of NMR data.^21,23^ However, upon repetition of this reaction, we found that the assignment was incorrect. The product was unambiguously reassigned to 1,3-diaminoisoquinoline *N*-oxide **4a** by X-ray crystallography.

**Scheme 2:**
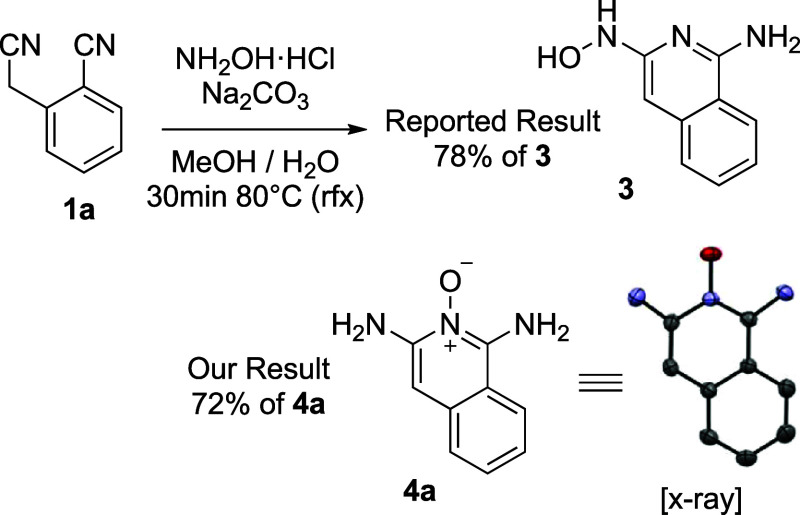
Structural Reassignment of the Reported Product Formed from Hydroxylamine and *o*-Cyanobenzyl Cyanide as 1,3-Diaminoisoquinoline *N*-Oxide

Following this structural assignment, we optimized the reaction conditions. In the previously reported conditions, hydroxylamine was generated in situ from hydroxylamine hydrochloride and sodium carbonate. However, we opted to use commercially available aqueous hydroxylamine for simplicity. Solvent screening was conducted to identify suitable media for the cyclization (Table 1). The reaction proceeded slowly in chloroform, while solvents such as THF, DMF, DMSO, MeOH, and EtOH all gave high yields of the desired product within 6 h. Notably, EtOH resulted in the fastest conversion and was chosen for further studies.

**Table 1:**
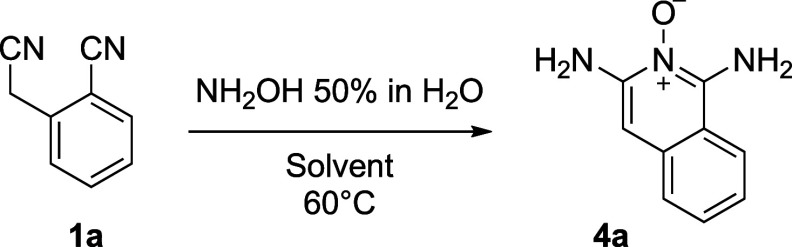
Solvent Screening^a^

		yield (%)^c^		
entry^b^	solvent	1 h	3 h	6 h
1	CHCl_3_	7	11	15
2	THF	55	83	92
3	DMF	65	87	93
4	DMSO	74	91	92
5	MeOH	78	90	94
6	EtOH	86	94	94

a 1,3,5-Trimethoxybenzene is used as the internal standard.

b The reaction was performed using **1a** (0.5 mmol), NH_2_OH 50% in H_2_O (1.0 mmol), 1,3,5-TMB (0.056 mmol), and solvent (1 mL). See the Supporting Information for details.

c Yield was calculated by ^1^H NMR analysis.

Using EtOH as the solvent for the formation of the isoquinoline *N*-oxide, we next developed a one-pot protocol to access the isoquinoline core. After completion of the *N*-oxide formation, the reaction mixture was cooled to 0 °C and B_2_pin_2_ ^24^ (2.2 equiv) was added; upon warming to room temperature, the N–O bond was reduced smoothly within 30 min to afford 1,3-diaminoisoquinoline. (Entry 1, Table 2)

**Table 2:**
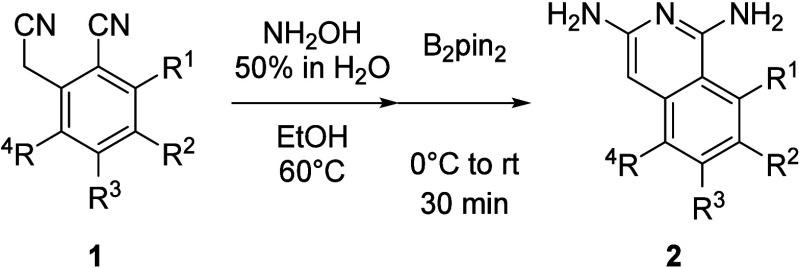
Substrate Scope

	**substrate**					
entry^a^/S.M.	**R** ^1^	**R** ^**2**^	**R** ^3^	**R** ^4^	**time (h)**	**yield** (%)^b^
1/**1a**	H	H	H	H	3	88
2/**1b**	OMe	H	H	H	3	80
3/**1c**	H	OMe	H	H	3	88
4/**1d**	H	H	OMe	H	3	91
5/**1e**	H	H	H	OMe	3	89
6/**1f**	H	OMe	OMe	H	3	86
7/**1g**	Br	H	H	H	1	70
8/**1h**	H	Br	H	H	1	88
9/**1i**	H	H	Br	H	1	78
10/**1j**	H	H	H	Br	1	91
11/**1k**	Me	H	H	H	3	88
12/**1l**	H	NO_2_	H	H	0.5	75^c^
13/**1m**	H	NH_2_	H	H	3	84
14/**1n**	H	H	3-Py	H	2	89
15/**1o**	H	H	H	CF_3_	1	88

a The reaction was performed using **1** (1.0 mmol), NH_2_OH 50% in H_2_O (2.0 mmol), EtOH (2 mL), and B_2_pin_2_ (2.2 mmol). See the Supporting Information for details.

b Isolated yield.

c The reduction with B_2_pin_2_ required 3 h.

After optimizing the conditions to obtain 1,3-diaminoisoquinoline, we explored the substrate scope (Table 2), with particular emphasis on methoxy-substituted substrates that had failed under the original ammonia conditions (Scheme 1b). Substrates bearing methoxy groups at various positions on the benzene ring were efficiently converted to the corresponding isoquinolines in high yields of 80–91% (entries 2–6, Table 2). These results highlight the advantages of the optimized method. Importantly, the method enabled the synthesis of 6,7-dimethoxy-1,3-diaminoisoquinoline **2f**. This substitution pattern is found in natural isoquinoline alkaloids such as papaverine, which are biosynthesized from dopamine bearing hydroxyl groups at these positions. Additionally, substrates bearing bromine substituents at various positions were compatible with the reaction conditions (entries 7–10, Table 2). The resulting bromo-substituted products offer potential for further diversification through cross-coupling reactions. Furthermore, substrates bearing methyl, nitro, amino, 3-pyridyl, and trifluoromethyl substituents also afforded the corresponding 1,3-diaminoisoquinolines in good yields (entries 11–15, Table 2).

To gain insight into the relative reactivity of the two amino groups in 1,3-diaminoisoquinoline, we carried out an acetylation study (Scheme 3). Under standard acetylation conditions, selective acylation at the 3-amino position was observed, and the structure of the monoacetylated product was unambiguously confirmed by single-crystal X-ray diffraction.

**Scheme 3:**
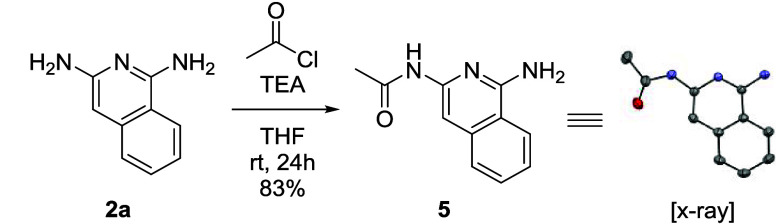
Regioselective Acetylation

In conclusion, we have developed a concise and operationally simple method for the synthesis of 1,3-diaminoisoquinoline and its derivatives via hydroxylamine mediated cyclization and one-pot reduction. This strategy utilizes only readily available reagents, proceeds under mild conditions, tolerates a variety of functional groups, including electron-donating and electron-withdrawing substituents, and enables rapid access to diverse isoquinoline scaffolds. Importantly, in contrast to the previous method utilizing ammonia, this approach exhibits high reactivity with electron-rich substrates and avoids the use of liquid ammonia. Furthermore, structural reassignment of a previously reported intermediate was achieved by X-ray crystallography, revealing that the actual product is the *N*-oxide **4a**. These findings collectively provide a robust platform for further exploration of 1,3-diaminoisoquinoline derivatives in medicinal and materials chemistry.

## Supplementary Material



## Data Availability

The data underlying this study are available in the published article and its Supporting Information. The raw data underlying this study are openly available in Zenodo at https://zenodo.org/records/20773748.

## Notes

General information, reaction optimization, experimental procedures, characterization data of compounds, crystallographic details, and NMR spectra (PDF)
