# Supplementary material for: Synthesis of 1,3-Diaminoisoquinoline Derivatives via N‑Oxide Intermediates from o‑Cyanobenzyl Cyanides
Source: J Org Chem. 2026 Jul 10;91(29):10303–6. doi: 10.1021/acs.joc.6c00531 (PMC13411050; doi:10.1021/acs.joc.6c00531)
Supplement: Supplementary file 1 [file jo6c00531_si_001.pdf]

Synthesis of 1,3-Diaminoisoquinoline Derivatives via *N*-Oxide Intermediates from *o*-Cyanobenzyl Cyanides

Daisuke Sakamoto,*[1,3] Alessandro Prescimone,[1] Philippe H. M. Marliere,[3] and Konrad Tiefenbacher[1,2]

[1] Department of Chemistry, University of Basel, Mattenstrasse 22, 4058 Basel, Switzerland
[2] Department of Biosystems Science and Engineering, ETH Zurich, Klingelbergstrasse 48, 4056 Basel, Switzerland
3 The European Syndicate of Synthetic Scientists and Industrialists (TESSSI), 81 Rue Réaumur, 75002 Paris, France

Table of Contents

1. General information S2
2. Synthesis of *o*-cyanobenzeneacetonitrile derivatives S3
   General procedure A: SNAr reaction of methyl cyanoacetate with o-fluorobenzonitrile S3
   General procedure B: Decarboxylation S3
   The synthesis of 5-amino-2-(cyanomethyl)benzonitrile (1m) S8
   The synthesis of 2-(cyanomethyl)-4-(pyridin-3-yl)benzonitrile (1n) S8
3. Formation of isoquinolines using NH_2OH and B_2pin_2 S9
   General procedure C: Formation of isoquinoline S9
4. Other experiments S17
   Isoquinoline formation using 7N NH_3/MeOH S17
   The reaction of o-cyanobenzyl cyanide and $NH_2OH·HCl$ in MeOH [1] S18
   Solvent screening for isoquinoline N-oxide formation (NMR experiment) S19
   Acetylation of 1,3-diaminoisoquinoline S19
5. Crystallographic details for 4a and 5 S20
6. ^{1}H and $^{13}C\{^{1}H\}$ NMR Chart S34
7. References S63

1. General information

Experimental: Reactions were carried out under an atmosphere of argon in dried glassware unless otherwise indicated. Analytical thin-layer chromatography (TLC) was performed on Merck silica gel 60 F254 glass-backed plates, which were analysed after exposure to standard staining solutions (CAM: cerium ammonium molybdate, anisaldehyde or basic $KMnO_4$). All NMR experiments were performed on a Bruker Avance Neo and a Bruker Avance III HD NMR spectrometer operating at 500 MHz and 600 MHz proton frequency, respectively. The instruments were equipped with a direct observe 5-mm BBFO smart probe (500 MHz) or a five-channel cryogenic 5 mm QCI probe (600 MHz). All probes were equipped with actively shielded z-gradients (10 A). The experiments were performed at 298 K, VT NMR measurements were performed at 373 K. Chemical shifts of ^1H NMR and ^{13}C NMR are given in ppm. The following solvent residual signals of the deuterated solvents were used as reference: $CDCl_3$: 7.26 ppm (δ^1H), 77.16 ppm ($\delta^{13}C$), DMSO-d_6: 2.50 ppm (δ^1H), 39.52 ppm ($\delta^{13}C$). Standard abbreviations indicating multiplicity were used as follows: s (singlet), d (doublet), t (triplet), dd (doublet of doublets), dt (doublet of triplets), dq (doublet of quartets), ddd (doublet of doublet of doublets), br-s (broad singlet), m (multiplet). HRMS (ESI) was measured on a Bruker maXis 4G instrument using methanol as the solvent. Microwave-assisted reactions were performed in sealed microwave vials using an Anton Paar Monowave 400 microwave reactor. Reaction temperatures were monitored by an IR sensor and controlled by the instrument software. Infrared spectra were measured on a Bruker Alpha IR spectrometer (attenuated total reflection, ATR). Abbreviations indicating intensity were used as follows: s (strong), m (medium), w (weak).

Sources of solvents: Deuterated chloroform ($CDCl_3$, 99.8%) and dimethyl sulfoxide D_6 (DMSO-d_6, 99.8%) were purchased from Eurisotop. Ethanol (99.5%, Extra Dry), methanol (99.8%, Extra Dry), dimethyl sulfoxide (99.7%+, Extra Dry), *N,N*-dimethylformamide (99.8%, Extra Dry) and tetrahydrofuran (99.5%, Extra Dry) were purchased from Thermo Scientific. Chloroform (J.T.Baker) was purchased from Avantor.

Sources of chemicals: Acetyl chloride, 1,3,5-trimethoxybenzene, and triethylamine were purchased from Sigma-Aldrich. Methyl cyanoacetate, Ammonia (ca. 7 N solution in methanol), and α-Cyano-*o*-toluonitrile (*o*-Cyanobenzyl cyanide) were purchased from Thermo Scientific. Iron, 99%, powder, -70 mesh was purchased from Acros Organics. Bis(pinacolato)diboron, 2-Bromo-6-fluorobenzonitrile, 4,5-Dimethoxy-2-fluorobenzonitrile, 2-Fluoro-5-methoxybenzonitrile, 2-Fluoro-6-methylbenzonitrile and 2-Fluoro-4-methoxybenzonitrile were purchased from Apollo Scientific. Sodium hydride (60% in Paraffin oil), 2-Fluoro-6-methoxybenzonitrile, 4-Bromo-2-cyanobenzeneacetonitrile, and 5-Bromo-2-cyanobenzeneacetonitrile were purchased from Fluorochem. 3-Bromo-2-fluorobenzonitrile, 2-Fluoro-5-nitrobenzonitrile, 2-Fluoro-3-(trifluoromethyl)benzonitrile, Pyridin-3-ylboronic acid, and 2-Fluoro-3-methoxybenzonitrile were purchased from BLD Pharm. Hydroxylamine hydrochloride was purchased from Alfa Aesar. SiliaFlash® Irregular Silica Gel (P60, 40 - 63 μm) was purchased from SiliCycle. Hydroxylamine (50% solution in water) was purchased from Fluka. Sodium chloride, hydrochloric acid (37 wt%), anhydrous sodium sulfate, sodium hydrogencarbonate, and ammonium chloride were purchased from VWR. All chemicals were used as received. Transfer of liquids with a volume ranging from 1 to 10 μL or from 10 to 100 μL was performed with a Microman M1 pipette (Gilson, systematic error: 1.40% - 1.60%) equipped with 10 μL or 100 μL pipette tips, respectively.

2. Synthesis of *o*-cyanobenzeneacetonitrile derivatives

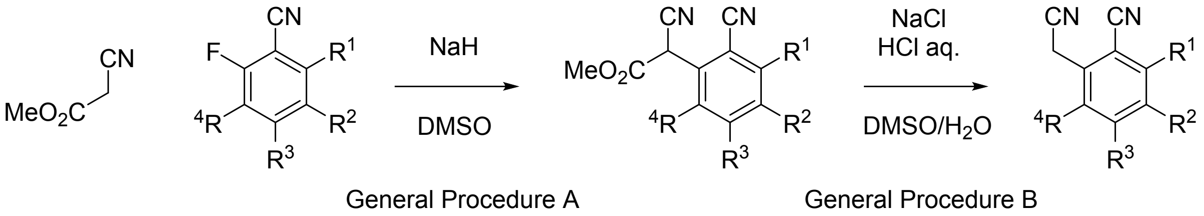

<u>General procedure A:</u> SNAr reaction of methyl cyanoacetate with *o*-fluorobenzonitrile

Methyl cyanoacetate (2.0 equiv. 20.0 mmol) was dissolved in DMSO (1.0 M) under an argon atmosphere. Sodium hydride (60% in mineral oil, 2.0 equiv. 20.0 mmol) was then slowly added to the mixture, and the resulting solution was stirred at room temperature for 30 minutes. Subsequently, *o*-fluorobenzonitrile (1.0 equiv. 10.0 mmol) was introduced to the mixture, followed by heating to 120°C in an oil bath. Upon completion of the reaction, the mixture was cooled to 0°C and quenched with 10 mL of 3M HCl aqueous solution. Extraction of the mixture was carried out using ethyl acetate (15 mL × 3), and the organic layer was subsequently washed with 45 mL of saturated NaCl solution (45 mL). After drying over $MgSO_4$, the organic layer was concentrated under reduced pressure, yielding the crude product, which was utilized in the subsequent reaction.

<u>General procedure B</u>: Decarboxylation

The crude product of General Procedure A was dissolved in DMSO (0.25 M) under an argon atmosphere. Sodium chloride (15.0 equiv.) and 1.5M HCl aqueous solution (1.0 equiv.) were then added to the mixture, and the resulting solution was stirred at 120 °C in an oil bath for 1 hour. Upon completion of the reaction, the mixture was cooled to 0°C and diluted with saturated NaCl aqueous solution. Extraction of the mixture was carried out using ethyl acetate (15 mL, thrice), and the organic layer was subsequently washed with saturated NaCl solution (45 mL, once). After drying over $MgSO_4$, the organic layer was concentrated under reduced pressure, yielding the crude product, which was purified with silica gel column chromatography.

2-(cyanomethyl)-6-methoxybenzonitrile (1b)

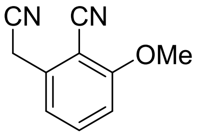

Chemical Formula: $C_{10}H_8N_2O$
Molecular Weight: 172.19

Silica gel column chromatography eluent: DCM.

Yield: 872 mg of a beige solid, 84% (6.00 mmol of starting material was used for the reaction.)

R_f 0.74 (Et_2O/DCM=1/9)

^1H NMR (500 MHz, DMSO-d_6) δ 7.71 (dd, *J* = 8.6, 7.7 Hz, 1H), 7.25 (dd, *J* = 8.7, 0.8 Hz, 1H), 7.19 (dd, *J* = 7.7, 0.8 Hz, 1H), 4.22 (s, 2H), 3.94 (s, 3H).

$^{13}C\{^1H\}$ NMR (126 MHz, DMSO-d_6) δ 161.8, 136.2, 135.3, 121.0, 117.5, 114.3, 111.7, 100.4, 56.6, 21.8.

HRMS (ESI$^+$) m/z calculated for $C_{10}H_8N_2NaO$ $[M + Na]^+$: 195.0529; found 195.0525.

IR (ATR) ν_{max}/cm^{-1} 2914 (m), 2851 (m), 2254 (w), 2219 (m), 1583 (s), 1474 (s), 1435 (s), 1272 (s), 1115 (w), 1066 (s), 782 (s), 736 (w), 716 (w).

2-(cyanomethyl)-5-methoxybenzonitrile (1c)

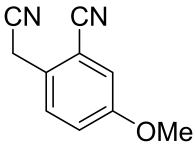

Chemical Formula: $C_{10}H_8N_2O$
Molecular Weight: 172.19

Silica gel column chromatography eluent: DCM/cyclohexane = 1:2 to 3:2

Yield: 236 mg of a yellow solid, 23% (6.00 mmol of starting material was used for the reaction.)

R_f 0.70 (Et_2O/DCM=1/9)

^{1}H NMR (500 MHz, DMSO-d_6) δ 7.55 (d, J = 8.7 Hz, 1H), 7.51 (d, J = 2.8 Hz, 1H), 7.33 (dd, J = 8.6, 2.8 Hz, 1H), 4.17 (s, 2H), 3.82 (s, 3H).

^{13}C{^{1}H} NMR (126 MHz, DMSO-d_6) δ 158.8, 131.0, 126.5, 120.3, 118.1, 117.8, 116.6, 112.4, 55.9, 20.9.

HRMS (ESI$^+$) m/z calculated for $C_{10}H_8N_2NaO$ [M + Na]$^+$: 195.0529; found 195.0529

IR (ATR) ν_{max}/cm^{-1} 3081 (w), 2914 (w), 2226 (m), 1606 (m), 1498 (m), 1461 (m), 1291 (s), 1255 (m), 1201 (m), 1084 (m), 1022 (m), 924 (m), 882 (m), 824 (s).

2-(cyanomethyl)-4-methoxybenzonitrile (1d)

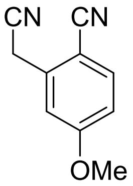

Chemical Formula: $C_{10}H_8N_2O$
Molecular Weight: 172.19

Silica gel column chromatography eluent: DCM/cyclohexane = 1:2 to 2:1

Yield: 1.06 g of a pale yellow solid, 61% (10.0 mmol of starting material was used for the reaction.)

R_f 0.64 (Et_2O/DCM=1/9)

^{1}H NMR (500 MHz, DMSO-d_6) δ 7.85 (d, J = 8.6 Hz, 1H), 7.19 (d, J = 2.5 Hz, 1H), 7.10 (dd, J = 8.7, 2.6 Hz, 1H), 4.21 (s, 2H), 3.87 (s, 3H).

^{13}C{^{1}H} NMR (126 MHz, DMSO-d_6) δ 163.0, 136.8, 135.4, 117.5, 117.1, 115.8, 114.2, 103.0, 56.0, 21.9.

HRMS (ESI$^+$) m/z calculated for $C_{10}H_8N_2NaO$ [M + Na]$^+$: 195.0529; found 195.0526

IR (ATR) ν_{max}/cm^{-1} 2219 (m), 1608 (m), 1565 (m), 1498 (m), 1287 (m), 1261 (m), 1188 (m), 1105 (m), 1026 (m), 865 (m), 829 (s), 683 (m).

2-(cyanomethyl)-3-methoxybenzonitrile (1e)

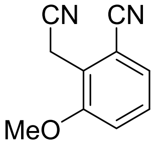

Chemical Formula: $C_{10}H_8N_2O$
Molecular Weight: 172.19

Silica gel column chromatography eluent: DCM/cyclohexane = 1:2 to 3:2

Yield: 1.29 g of a pale yellow solid, 75% (10.0 mmol of starting material was used for the reaction.)

R_f 0.72 (Et_2O/DCM=1/9)

^1H NMR (500 MHz, DMSO-d_6) δ 7.57 (t, J = 8.1 Hz, 1H), 7.50 – 7.46 (m, 2H), 4.00 (s, 2H), 3.93 (s, 3H).

$^{13}C\{^1H\}$ NMR (126 MHz, DMSO-d_6) δ 157.1, 130.9, 124.8, 122.1, 117.0, 116.9, 116.7, 112.8, 56.5, 16.4.

HRMS (ESI$^+$) m/z calculated for $C_{10}H_8N_2NaO$ [M + Na]$^+$: 195.0529; found 195.0530

IR (ATR) ν_{max}/cm^{-1} 2232 (m), 1581 (s), 1467 (s), 1435 (m), 1271 (s), 1068 (s), 791 (s), 737 (m), 683 (m).

2-(cyanomethyl)-4,5-dimethoxybenzonitrile (1f)

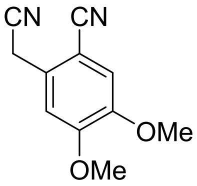

Chemical Formula: $C_{11}H_{10}N_2O_2$
Molecular Weight: 202.21

Silica gel column chromatography eluent: DCM/pentane = 1:2 to 4:1

Yield: 697 mg of a pale yellow solid, 35% (10.0 mmol of starting material was used for the reaction.)

R_f 0.55 (Et_2O/DCM=1/9)

^1H NMR (500 MHz, DMSO-d_6) δ 7.45 (s, 1H), 7.23 (s, 1H), 4.13 (s, 2H), 3.86 (s, 3H), 3.81 (s, 3H).

$^{13}C\{^1H\}$ NMR (126 MHz, DMSO-d_6) δ 152.8, 148.4, 128.7, 117.8, 117.2, 115.3, 112.9, 102.7, 56.1, 56.0, 21.3.

HRMS (ESI$^+$) m/z calculated for $C_{11}H_{10}N_2NaO_2$ [M + Na]$^+$: 225.0634; found 225.0636

IR (ATR) ν_{max}/cm^{-1} 2912 (w), 2219 (m), 1601 (m), 1517 (s), 1443 (m), 1409 (m), 1354 (m), 1310 (m), 1268 (s), 1220 (s), 1186 (s), 1086 (s), 984 (s), 857 (s), 745 (m), 635 (m).

2-bromo-6-(cyanomethyl)benzonitrile (1g)

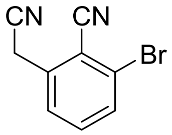

Chemical Formula: $C_9H_5BrN_2$
Molecular Weight: 221.06

Silica gel column chromatography eluent: DCM/pentane = 1:2 to 2:1, then DCM

Yield: 1.77 g of a beige solid, 80% (10.0 mmol of starting material was used for the reaction.)

R_f 0.73 (Et_2O/DCM=1/9)

^{1}H NMR (500 MHz, DMSO-d_6) δ 7.89 (dd, *J* = 7.8, 1.4 Hz, 1H), 7.70 (t, *J* = 7.8 Hz, 1H), 7.68 – 7.65 (m, 1H), 4.34 (s, 2H).

$^{13}C\{^1H\}$ NMR (126 MHz, DMSO-d_6) δ 137.8, 135.2, 132.5, 128.4, 125.7, 117.2, 115.3, 114.6, 22.5.

HRMS (ESI$^+$) m/z calculated for $C_9H_5BrN_2Na$ [M + Na]$^+$: 242.9528; found 242.9531

IR (ATR) ν_{max}/cm^{-1} 2931 (w), 2912 (w), 2250 (w), 2227 (w), 1587 (w), 1558 (m), 1437 (m), 1401 (m), 827 (m), 788 (s), 716 (w), 667 (m)

3-bromo-2-(cyanomethyl)benzonitrile (1j)

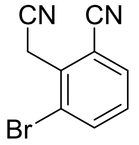

Chemical Formula: $C_9H_5BrN_2$
Molecular Weight: 221.06

Silica gel column chromatography eluent: DCM/pentane = 1:2 to 2:1, then DCM

Yield: 1.70 g of a beige solid, 77% (10.0 mmol of starting material was used for the reaction.)

R_f 0.73 (Et_2O/DCM=1/9)

^{1}H NMR (500 MHz, DMSO-d_6) δ 8.09 (dd, *J* = 8.1, 1.2 Hz, 1H), 7.99 (dd, *J* = 7.7, 1.2 Hz, 1H), 7.53 (t, *J* = 8.0 Hz, 1H), 4.25 (s, 2H).

$^{13}C\{^1H\}$ NMR (126 MHz, DMSO-d_6) δ 138.2, 133.8, 133.2, 131.1, 124.8, 116.3, 116.0, 114.1, 23.3.

HRMS (ESI$^+$) m/z calculated for $C_9H_5BrN_2Na$ [M + Na]$^+$: 242.9528; found 242.9529

IR (ATR) ν_{max}/cm^{-1} 2966 (w), 2231 (w), 1557 (w), 1451 (w), 1434 (w), 1406 (m), 1118 (m), 913 (m), 793 (s), 724 (m), 638 (w).

2-(cyanomethyl)-6-methylbenzonitrile (1k)

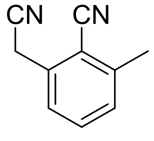

Chemical Formula: $C_{10}H_8N_2$
Molecular Weight: 156.1880

Silica gel column chromatography eluent: DCM/cyclohexane = 1:2 to 1:1

Yield: 971 mg of a white solid, 62% (10.0 mmol of starting material was used for the reaction.)

R_f 0.83 (Et_2O/DCM=5/95)

^{1}H NMR (500 MHz, DMSO-d_6) δ 7.64 (t, *J* = 7.8 Hz, 1H), 7.49 – 7.43 (m, 2H), 4.25 (s, 2H), 2.51 (s, 3H).

$^{13}C\{^1H\}$ NMR (126 MHz, DMSO-d_6) δ 143.4, 135.4, 133.9, 130.2, 127.2, 118.1, 116.3, 112.4, 22.5, 20.7.

HRMS (ESI$^+$) m/z calculated for $C_{10}H_9N_2$ [M + H]$^+$: 157.0760; found 157.0760.

IR (ATR) ν_{max}/cm^{-1} 2928 (w), 2251 (w), 2223 (m), 1591 (w), 1464 (m), 1405 (w), 1378 (w), 1316 (w), 1243 (w), 1218 (w), 1168 (w), 1039 (w), 785 (s), 595 (w), 565 (w).

2-(cyanomethyl)-5-nitrobenzonitrile (1l)

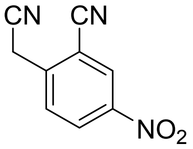

Chemical Formula: $C_9H_5N_3O_2$
Molecular Weight: 187.1580

Silica gel column chromatography eluent: EtOAc/cyclohexane = 1:10 to 1:4

Yield: 900 mg of a dark yellow solid, 48% (10.0 mmol of starting material was used for the reaction.)

R_f 0.71 (Et_2O/DCM=5/95)

^{1}H NMR (500 MHz, DMSO-d_6) δ 8.82 (d, J = 2.5 Hz, 1H), 8.58 (dd, J = 8.6, 2.5 Hz, 1H), 7.92 (dd, J = 8.7, 0.6 Hz, 1H), 4.48 (s, 2H).

^{13}C{^{1}H} NMR (126 MHz, DMSO-d_6) δ 147.1, 141.7, 130.9, 128.6, 128.6, 116.9, 115.2, 113.0, 22.2.

HRMS (ESI$^-$) m/z calculated for $C_9H_4N_3O_2$ [M - H]$^-$: 186.0309; found 186.0312.

IR (ATR) ν_{max}/cm^{-1} 3080 (w), 2917 (w), 2257 (w), 2234 (w), 1524 (s), 1346 (s), 930 (m), 828 (m), 801 (m), 740 (m).

2-(cyanomethyl)-3-(trifluoromethyl)benzonitrile (1o)

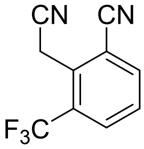

Chemical Formula: $C_{10}H_5F_3N_2$
Molecular Weight: 210.1592

Silica gel column chromatography eluent: DCM/cyclohexane = 1:2 to 1:1

Yield: 1.46 g of a pale yellow solid, 69% (10.0 mmol of starting material was used for the reaction.)

R_f 0.83 (Et_2O/DCM=5/95)

^{1}H NMR (500 MHz, DMSO-d_6) δ 8.31 (d, J = 7.9 Hz, 1H), 8.18 (d, J = 7.5 Hz, 1H), 7.84 (t, J = 7.9 Hz, 1H), 4.28 (s, 2H).

^{13}C{^{1}H} NMR (126 MHz, DMSO-d_6) δ 137.8 (q, $J_{C–F}$ = 0.8 Hz), 132.8 (q, $J_{C–F}$ = 1.7 Hz), 131.4 (q, $J_{C–F}$ = 5.5 Hz), 130.3, 128.8 (q, $J_{C–F}$ = 31.1 Hz), 123.6 (q, $J_{C–F}$ = 275 Hz), 116.2, 115.8, 115.4, 19.5 (q, $J_{C–F}$ = 2.5 Hz).

HRMS (ESI$^+$) m/z calculated for $C_{10}H_6F_3N_2$ [M + H]$^+$: 211.0478; found 211.0475.

IR (ATR) ν_{max}/cm^{-1} 3083 (w), 3038 (w), 2993 (w), 2953 (w), 2261 (w), 2233 (w), 1456 (m), 1435 (m), 1317 (s), 1206 (m), 1163 (m), 1130 (m), 1101 (s), 940 (m), 820 (s), 756 (m), 717 (s).

The synthesis of 5-amino-2-(cyanomethyl)benzonitrile (1m)

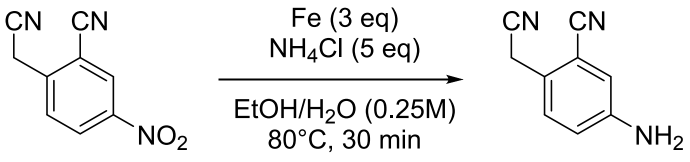

1l **1m**

2-(Cyanomethyl)-5-nitrobenzonitrile (**1l**) (1.0 equiv, 1.50 mmol, 281 mg), ammonium chloride (5.0 equiv, 7.50 mmol, 401 mg), and iron (3.0 equiv, 4.50 mmol, 251 mg) were added to ethanol (3.0 mL) and water (3.0 mL). The mixture was heated to 80 °C in an oil bath and stirred for 30 min. The reaction mixture was then cooled to room temperature and filtered through Celite. The filtrate was concentrated under reduced pressure, and the residue was purified by column chromatography on silica gel (DCM/cyclohexane = 1:1 to 100% DCM) to afford 5-amino-2-(cyanomethyl)benzonitrile **1m** (54.1 mg, pale yellow solid, 23%).

5-amino-2-(cyanomethyl)benzonitrile (1m)

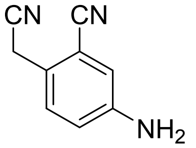

Chemical Formula: $C_9H_7N_3$
Molecular Weight: 157.1760

R_f 0.09 (EtOAc/Cyclohexane=1/2)

^{1}H NMR (500 MHz, DMSO-d_6) δ 7.24 (d, J = 8.4 Hz, 1H), 6.92 (d, J = 2.5 Hz, 1H), 6.86 (dd, J = 8.4, 2.5 Hz, 1H), 5.69 (s, 2H), 3.98 (s, 2H).

$^{13}C\{^1H\}$ NMR (126 MHz, DMSO-d_6) δ 149.0, 130.5, 120.0, 118.6, 118.3, 117.4, 116.8, 111.6, 20.7.

HRMS (ESI$^+$) m/z calculated for $C_9H_7N_3Na$ [M + Na]$^+$: 180.0532; found 180.0531.

IR (ATR) ν_{max}/cm^{-1} 3442 (m), 3354 (m), 2223 (m), 1631 (s), 1572 (m), 1503 (s), 1411 (m), 1318 (m), 1263 (m), 858 (m), 834 (s).

The synthesis of 2-(cyanomethyl)-4-(pyridin-3-yl)benzonitrile (1n)

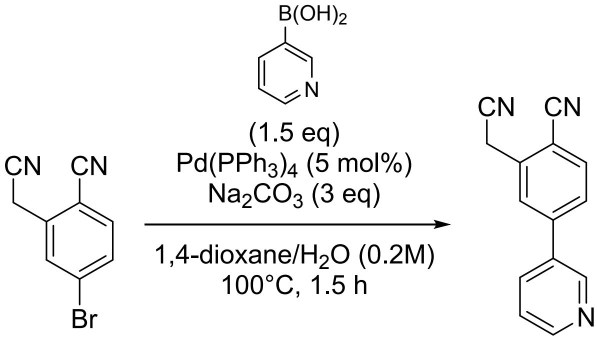

1i **1n**

1,4-Dioxane (6.0 mL) and water (1.5 mL) were added to a 30 mL one-neck flask, and the mixture was bubbled with argon for 15 min. 4-Bromo-2-(cyanomethyl)benzonitrile (1.0 equiv, 1.50 mmol, 332 mg), pyridin-3-ylboronic acid (1.5 equiv, 2.25 mmol, 277 mg), $Pd(PPh_3)_4$ (5.0 mol%, 0.075 mmol, 86.7 mg), and Na_2CO_3 (3.0 equiv, 4.50 mmol, 477 mg) were then added, and the mixture was heated at 100 °C in an oil bath and stirred for 1.5 h. The reaction mixture was cooled to room temperature and

filtered through Celite, rinsing with DCM and MeOH. The filtrate was concentrated under reduced pressure, and the crude material was purified by silica gel column chromatography (EtOAc/cyclohexane = 1:1 to 2:1) to afford 2-(cyanomethyl)-4-(pyridin-3-yl)benzonitrile **1n** (312 mg, pale yellow solid, 95%).

2-(cyanomethyl)-4-(pyridin-3-yl)benzonitrile (1n)

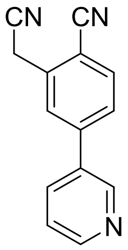

Chemical Formula: $C_{14}H_9N_3$
Molecular Weight: 219.2470

R_f 0.16 (EtOAc/Cyclohexane=2/1)

^{1}H NMR (500 MHz, DMSO-d$_6$) δ 8.97 (dd, *J* = 2.4, 0.9 Hz, 1H), 8.67 (dd, *J* = 4.8, 1.6 Hz, 1H), 8.17 (ddd, *J* = 8.0, 2.5, 1.6 Hz, 1H), 8.06 (d, *J* = 8.1 Hz, 1H), 8.03 (d, *J* = 1.9 Hz, 1H), 7.95 (dd, *J* = 8.1, 1.8 Hz, 1H), 7.56 (ddd, *J* = 8.0, 4.8, 0.9 Hz, 1H), 4.33 (s, 2H).

^{13}C{^{1}H} NMR (126 MHz, DMSO-d$_6$) δ 150.0, 148.0, 142.3, 135.6, 134.7, 134.3, 133.5, 128.3, 127.2, 124.1, 117.5, 116.7, 111.1, 22.0.

HRMS (ESI$^+$) m/z calculated for $C_{14}H_{10}N_3$ [M + H]$^+$: 220.0869; found 220.0871.

IR (ATR) ν_{max}/cm^{-1} 2920 (w), 2225 (m), 1607 (m), 1574 (w), 1479 (m), 1426 (m), 1394 (m), 849 (s), 804 (s), 712 (s).

3. Formation of isoquinolines using NH_2OH and B_2pin_2

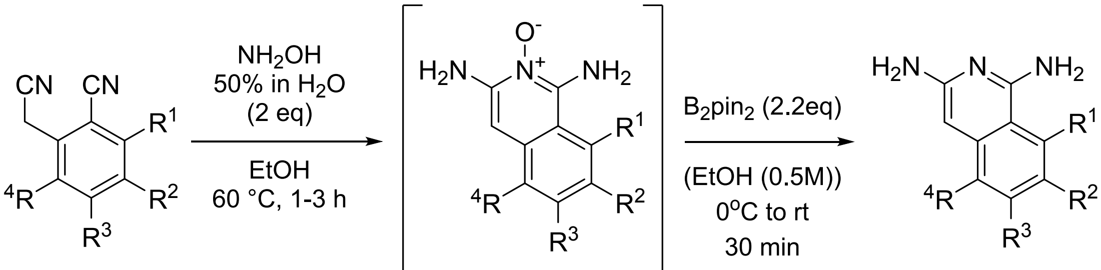

General procedure C: Formation of isoquinoline

o-Cyanobenzyl cyanide derivative (1 equiv. 1.00 mmol) was dissolved in EtOH (0.5 M, 2.0 mL), and 50% aqueous NH_2OH solution (2 equiv. 2.00 mmol, 123 µL) was added. The reaction mixture was heated at 60 °C in an oil bath and monitored by TLC. After complete consumption of the starting material, the reaction was cooled to 0 °C. Bis(pinacolato)diboron (2.2 equiv. 2.20 mmol, 559 mg) was added, and the mixture was stirred at room temperature for 30 min. The volatiles were removed under reduced pressure, and the crude product was purified by column chromatography on silica gel. When an impurity derived from B_2pin_2 remained after purification, it was removed by heating at 60°C in an oil bath under reduced pressure (0.5 mbar).

isoquinoline-1,3-diamine (2a)

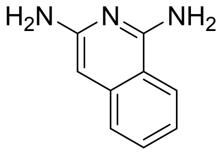

Chemical Formula: $C_9H_9N_3$
Molecular Weight: 159.19

Silica gel column chromatography eluent: MeOH/DCM = 1:50 to 1:10

Yield: 140 mg of a beige solid, 88%

R_f 0.20 (MeOH/DCM=1/9)

^{1}H NMR (500 MHz, DMSO-d$_6$) δ 7.89 (dd, J = 8.2, 1.0 Hz, 1H), 7.28 (ddd, J = 7.9, 6.5, 1.2 Hz, 1H), 7.23 (dd, J = 8.2, 1.3 Hz, 1H), 6.91 (ddd, J = 8.2, 6.6, 1.4 Hz, 1H), 6.46 (s, 2H), 5.83 (d, J = 0.8 Hz, 1H), 5.29 (s, 2H).

^{13}C{^{1}H} NMR (126 MHz, DMSO-d$_6$) δ 157.0, 155.0, 140.5, 129.5, 124.2, 123.9, 119.3, 111.4, 87.0.

HRMS (ESI$^+$) m/z calculated for $C_9H_{10}N_3$ [M + H]$^+$: 160.0869; found 160.0869

IR (ATR) ν_{max}/cm^{-1} 3415 (m), 3321 (w), 3173 (w), 1619 (m), 1557 (m), 1504 (m), 1428 (s), 1342 (m), 802 (m), 696 (m).

8-methoxyisoquinoline-1,3-diamine (2b)

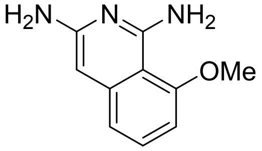

Chemical Formula: $C_{10}H_{11}N_3O$
Molecular Weight: 189.22

Silica gel column chromatography eluent: MeOH/DCM = 1:50 to 1:20

Yield: 152 mg of a yellowish brown solid, 80%

R_f 0.23 (MeOH/DCM=1/9)

^{1}H NMR (500 MHz, DMSO-d$_6$) δ 7.14 (t, *J* = 7.9 Hz, 1H), 6.78 (d, *J* = 8.2 Hz, 1H), 6.60 (br-s, 2H), 6.37 (d, *J* = 7.8 Hz, 1H), 5.75 (s, 1H), 5.26 (s, 2H), 3.87 (s, 3H).

^{13}C{^{1}H} NMR (126 MHz, DMSO-d$_6$) δ 157.8, 156.4, 155.2, 143.4, 129.7, 116.8, 102.4, 99.6, 87.2, 55.5.

HRMS (ESI$^+$) m/z calculated for $C_{10}H_{12}N_3O$ [M + H]$^+$: 190.0975; found 190.0972

IR (ATR) ν_{max}/cm^{-1} 3463 (w), 3317 (w), 3180 (w), 1603 (w), 1557 (m), 1491 (w), 1270 (w), 1249 (w), 1189 (w), 1098 (w), 1057 (w), 808 (w), 755 (w), 678 (w).

7-methoxyisoquinoline-1,3-diamine (2c)

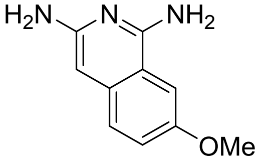

Chemical Formula: $C_{10}H_{11}N_3O$
Molecular Weight: 189.22

Silica gel column chromatography eluent: MeOH/DCM = 1:50 to 1:20

Yield: 166 mg of a dark brown solid, 88%

R_f 0.22 (MeOH/DCM=1/9)

^{1}H NMR (500 MHz, DMSO-d_6) δ 7.34 (d, J = 2.5 Hz, 1H), 7.22 (d, J = 8.9 Hz, 1H), 7.01 (dd, J = 8.9, 2.5 Hz, 1H), 6.33 (s, 2H), 5.84 (d, J = 0.8 Hz, 1H), 5.04 (s, 2H), 3.78 (s, 3H).

^{13}C{^{1}H} NMR (126 MHz, DMSO-d_6) δ 155.9, 153.3, 153.2, 135.7, 125.6, 121.6, 111.4, 103.6, 87.3, 55.3.

HRMS (ESI$^+$) m/z calculated for $C_{10}H_{12}N_3O$ [M + H]$^+$: 190.0975; found 190.0974

IR (ATR) ν_{max}/cm^{-1} 3451 (w), 3398 (w), 3361 (w), 3306 (w), 3171 (w), 1596 (m), 1558 (w), 1507 (w), 1418 (m), 1230 (m), 1147 (w), 1110 (w), 1067 (w), 1032 (w), 828 (w), 679 (w)

6-methoxyisoquinoline-1,3-diamine (2d)

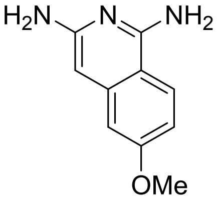

Chemical Formula: $C_{10}H_{11}N_3O$
Molecular Weight: 189.22

Silica gel column chromatography eluent: MeOH/DCM = 1:50 to 1:20

Yield: 173 mg of a beige solid, 91%

R_f 0.44 (MeOH/DCM=1/9)

^{1}H NMR (500 MHz, DMSO-d_6) δ 7.80 (d, J = 9.0 Hz, 1H), 6.63 (d, J = 2.6 Hz, 1H), 6.52 (dd, J = 9.0, 2.5 Hz, 1H), 6.30 (s, 2H), 5.78 (s, 1H), 5.23 (s, 2H), 3.77 (s, 3H).

^{13}C{^{1}H} NMR (126 MHz, DMSO-d_6) δ 160.1, 156.8, 155.5, 142.4, 126.0, 111.1, 106.5, 102.4, 87.2, 54.9.

HRMS (ESI$^+$) m/z calculated for $C_{10}H_{12}N_3O$ [M + H]$^+$: 190.0975; found 190.0978

IR (ATR) ν_{max}/cm^{-1} 3419 (w), 3325 (w), 3176 (w), 1615 (m), 1563 (w), 1501 (m), 1418 (m), 1236 (m), 1026 (m), 843 (m), 790 (m).

5-methoxyisoquinoline-1,3-diamine (2e)

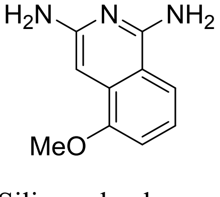

Chemical Formula: $C_{10}H_{11}N_3O$
Molecular Weight: 189.22

Silica gel column chromatography eluent: MeOH/DCM = 1:50 to 1:20

Yield: 169 mg of a brown solid, 89%

R_f 0.28 (MeOH/DCM=1/9)

^{1}H NMR (500 MHz, DMSO-d_6) δ 7.47 (dt, J = 8.3, 1.0 Hz, 1H), 6.84 (dd, J = 8.3, 7.5 Hz, 1H), 6.78 (dd, J = 7.6, 0.9 Hz, 1H), 6.35 (s, 2H), 6.09 (d, J = 0.9 Hz, 1H), 5.27 (s, 2H), 3.83 (s, 3H).

$^{13}C\{^1H\}$ NMR (126 MHz, DMSO-d_6) δ 156.8, 154.9, 152.4, 132.8, 118.7, 116.1, 111.6, 107.0, 81.9, 55.2.

HRMS (ESI$^+$) m/z calculated for $C_{10}H_{12}N_3O$ [M + H]$^+$: 190.0975; found 190.0974

IR (ATR) ν_{max}/cm^{-1} 3412 (m), 3328 (m), 3172 (w), 2917 (w), 2818 (w), 1605 (s), 1562 (m), 1500 (s), 1459 (m), 1418 (m), 1250 (s), 1214 (m), 1062 (m), 1000 (m), 820 (m), 799 (m), 724 (m).

6,7-dimethoxyisoquinoline-1,3-diamine (2f)

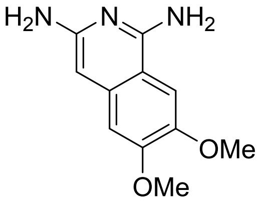

Chemical Formula: $C_{11}H_{13}N_3O_2$
Molecular Weight: 219.24

Silica gel column chromatography eluent: MeOH/DCM = 1:20 to 1:10

Yield: 189 mg of a brown solid, 86%

R_f 0.09 (MeOH/DCM=1/9)

^1H NMR (500 MHz, DMSO-d_6) δ 7.30 (s, 1H), 6.69 (s, 1H), 6.19 (s, 2H), 5.81 (s, 1H), 5.04 (s, 2H), 3.79 (s, 3H), 3.77 (s, 3H).

$^{13}C\{^1H\}$ NMR (126 MHz, DMSO-d_6) δ 155.5, 153.8, 152.2, 144.7, 136.9, 105.2, 104.5, 103.3, 87.5, 55.7, 55.2.

HRMS (ESI$^+$) m/z calculated for $C_{11}H_{14}N_3O_2$ [M + H]$^+$: 220.1081; found 220.1083

IR (ATR) ν_{max}/cm^{-1} 3438 (w), 3382 (w), 3110 (w), 1610 (m), 1566 (w), 1507 (w), 1416 (m), 1289 (w), 1245 (w), 1217 (m), 1182 (w), 1113 (w), 1050 (w), 855 (m), 835 (w), 775 (w).

8-bromoisoquinoline-1,3-diamine (2g)

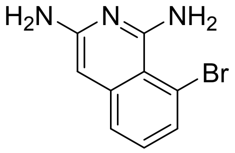

Chemical Formula: $C_9H_8BrN_3$
Molecular Weight: 238.09

Silica gel column chromatography eluent: MeOH/DCM = 1:100 to 1:50

Yield: 167 mg of a yellow solid, 70%

R_f 0.53 (MeOH/DCM=1/9)

^1H NMR (500 MHz, DMSO-d_6) δ 7.24 (dd, *J* = 8.3, 1.3 Hz, 1H), 7.16 (dd, *J* = 7.3, 1.2 Hz, 1H), 7.05 (dd, *J* = 8.2, 7.3 Hz, 1H), 6.61 (s, 2H), 5.93 (s, 1H), 5.56 (s, 2H).

$^{13}C\{^1H\}$ NMR (126 MHz, DMSO-d_6) δ 155.5, 155.0, 144.1, 129.6, 126.0, 124.9, 118.6, 108.6, 89.0.

HRMS (ESI$^+$) m/z calculated for $C_9H_9BrN_3$ [M + H]$^+$: 237.9974; found 237.9976

IR (ATR) ν_{max}/cm^{-1} 3469 (w), 3391 (w), 3307 (m), 3185 (w), 1601 (m), 1536 (s), 1494 (w), 1427 (m), 1345 (m), 916 (m), 811 (m), 670 (m).

7-bromoisoquinoline-1,3-diamine (2h)

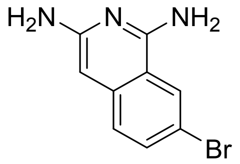

Chemical Formula: $C_9H_8BrN_3$
Molecular Weight: 238.09

Silica gel column chromatography eluent: MeOH/DCM = 1:50 to 1.5:50

Yield: 209 mg of a yellowish brown solid, 88%

R_f 0.42 (MeOH/DCM=1/9)

^{1}H NMR (500 MHz, DMSO-d$_6$) δ 8.15 (d, *J* = 2.0 Hz, 1H), 7.36 (dd, *J* = 8.8, 2.0 Hz, 1H), 7.20 (d, *J* = 8.8 Hz, 1H), 6.55 (s, 2H), 5.83 (d, *J* = 0.8 Hz, 1H), 5.42 (s, 2H).

^{13}C{^{1}H} NMR (126 MHz, DMSO-d$_6$) δ 156.3, 155.7, 139.3, 132.3, 126.4, 126.3, 112.3, 111.0, 86.9

HRMS (ESI$^+$) m/z calculated for $C_9H_9BrN_3$ [M + H]$^+$: 237.9974; found 237.9974

IR (ATR) ν_{max}/cm^{-1} 3406 (w), 3341 (w), 3128 (m), 1609 (m), 1549 (m), 1496 (m), 1428 (m), 869 (m), 831 (m), 701 (m), 594 (m).

6-bromoisoquinoline-1,3-diamine (2i)

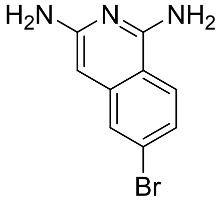

Chemical Formula: $C_9H_8BrN_3$
Molecular Weight: 238.09

Silica gel column chromatography eluent: MeOH/DCM = 1:50 to 1:20

Yield: 187 mg of a yellowish brown solid, 78%

R_f 0.31 (MeOH/DCM=1/9)

^{1}H NMR (500 MHz, DMSO-d$_6$) δ 7.82 (d, *J* = 8.9 Hz, 1H), 7.45 (d, *J* = 2.0 Hz, 1H), 6.97 (dd, *J* = 8.7, 2.0 Hz, 1H), 6.56 (s, 2H), 5.78 (s, 1H), 5.47 (s, 2H).

^{13}C{^{1}H} NMR (126 MHz, DMSO-d$_6$) δ 157.2, 156.2, 142.2, 126.6, 125.4, 123.7, 121.7, 109.7, 86.1.

HRMS (ESI$^+$) m/z calculated for $C_9H_9BrN_3$ [M + H]$^+$: 237.9974; found 237.9978

IR (ATR) ν_{max}/cm^{-1} 3416 (m), 3335 (w), 3293 (w), 3168 (w), 1605 (m), 1555 (s), 1486 (m), 1413 (m), 1330 (m), 905 (m), 873 (m), 794 (m), 665 (w)

5-bromoisoquinoline-1,3-diamine (2j)

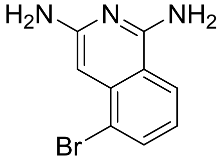

Chemical Formula: $C_9H_8BrN_3$
Molecular Weight: 238.09

Silica gel column chromatography eluent: MeOH/DCM = 1:50 to 1.5:50

Yield: 218 mg of a beige solid, 91%

R_f 0.39 (MeOH/DCM=1/9)

^{1}H NMR (500 MHz, DMSO-d_6) δ 7.93 (dt, *J* = 8.3, 1.1 Hz, 1H), 7.63 (dd, *J* = 7.4, 1.0 Hz, 1H), 6.80 (dd, *J* = 8.3, 7.4 Hz, 1H), 6.65 (s, 2H), 6.03 (d, J = 0.9 Hz, 1H), 5.64 (s, 2H).

^{13}C{^{1}H} NMR (126 MHz, DMSO-d_6) δ 157.8, 156.6, 139.3, 133.4, 124.3, 119.3, 118.0, 112.5, 85.7.

HRMS (ESI$^+$) m/z calculated for $C_9H_9BrN_3$ [M + H]$^+$: 237.9974; found 237.9970

IR (ATR) ν_{max}/cm^{-1} 3411 (m), 3321 (w), 3163 (w), 1613 (m), 1552 (w), 1485 (m), 1411 (m), 794 (m), 735 (m).

8-methylisoquinoline-1,3-diamine (2k)

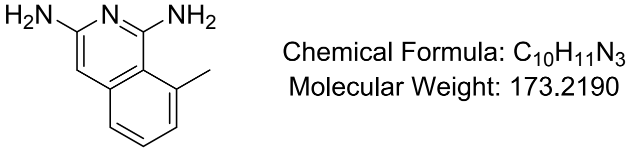

Silica gel column chromatography eluent: MeOH/DCM = 1:50 to 1:2

Yield: 153 mg of a yellowish brown solid, 88%

R_f 0.27 (MeOH/DCM=1/9)

^{1}H NMR (500 MHz, DMSO-d_6) δ 7.13–7.04 (m, 2H), 6.68 (d, *J* = 6.4 Hz, 1H), 5.88 (s, 1H), 5.85 (s, 2H), 5.25 (s, 2H), 2.73 (s, 3H).

^{13}C{^{1}H} NMR (126 MHz, DMSO-d_6) δ 158.1, 154.5, 143.1, 135.6, 129.3, 123.5, 123.3, 112.5, 89.7, 24.5.

HRMS (ESI$^+$) m/z calculated for $C_{10}H_{12}N_3$ [M + H]$^+$: 174.1026; found 174.1028.

IR (ATR) ν_{max}/cm^{-1} 3513 (m), 3363 (w), 3305 (w), 3182 (m), 1603 (m), 1558 (s), 1494 (m), 1452 (w), 1427 (m), 1392 (w), 1354 (m), 1132 (m), 815 (m), 761 (w), 675 (m).

7-nitroisoquinoline-1,3-diamine (2l)

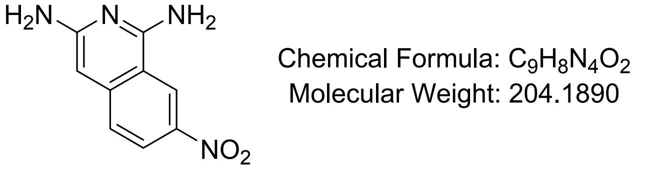

Silica gel column chromatography eluent: MeOH/DCM = 1:50 to 1:10

Yield: 154 mg of a brown solid, 75%

R_f 0.40 (MeOH/DCM=1/9)

^{1}H NMR (500 MHz, DMSO-d_6) δ 9.00 (d, *J* = 2.2 Hz, 1H), 7.92 (dd, *J* = 9.3, 2.3 Hz, 1H), 7.24 (d, *J* = 9.2 Hz, 1H), 7.20 (s, 2H), 6.30 (s, 2H), 5.91 (s, 1H).

^{13}C{^{1}H} NMR (126 MHz, DMSO-d_6) δ 159.8, 159.7, 143.8, 138.3, 124.1, 123.3, 123.2, 108.7, 88.4.

HRMS (ESI$^+$) m/z calculated for $C_9H_9N_4O_2$ [M + H]$^+$: 205.0720; found 205.0722.

IR (ATR) ν_{max}/cm^{-1} 3472 (m), 3431 (m), 3379 (m), 3151 (m), 1669 (m), 1612 (s), 1510 (m), 1468 (s), 1300 (s), 1193 (m), 1089 (m), 831 (s), 739 (m).

isoquinoline-1,3,7-triamine (2m)

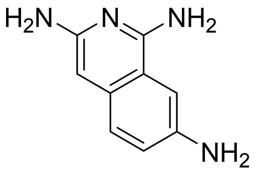

Chemical Formula: $C_9H_{10}N_4$
Molecular Weight: 174.2070

Silica gel column chromatography eluent: NEt_3/MeOH/DCM = 1:4:95 to 1:9:90

Yield: 51.4 mg of a yellowish-black solid, 84% (0.35 mmol of starting material was used for the reaction).

R_f 0.17 (H_2O/MeOH/DCM=1/3/10)

^{1}H NMR (500 MHz, DMSO-d_6) δ 7.08 (d, J = 8.6 Hz, 1H), 6.90 (d, J = 2.2 Hz, 1H), 6.85 (dd, J = 8.6, 2.2 Hz, 1H), 5.93 (s, 2H), 5.78 (s, 1H), 4.77 (br-s, 2H), 4.71 (br-s, 2H).

$^{13}C\{^1H\}$ NMR (126 MHz, DMSO-d_6) δ 154.9, 151.4, 141.9, 133.2, 125.0, 122.0, 112.9, 104.2, 88.0.

HRMS (ESI$^+$) m/z calculated for $C_9H_{11}N_4$ $[M + H]^+$: 175.0978; found 175.0981.

IR (ATR) ν_{max}/cm^{-1} 3413 (w), 3334 (w), 1612 (m), 1558 (w), 1514 (w), 1437 (m), 1340 (m), 830 (m), 792 (w), 732 (w), 703 (w).

6-(pyridin-3-yl)isoquinoline-1,3-diamine (2n)

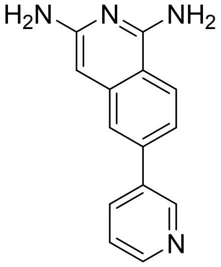

Chemical Formula: $C_{14}H_{12}N_4$
Molecular Weight: 236.2780

Silica gel column chromatography eluent: MeOH/DCM = 1:20 to 1:10

Yield: 211 mg of a yellow solid, 89%

R_f 0.11 (MeOH/DCM=1/9)

^{1}H NMR (500 MHz, DMSO-d_6) δ 8.96 (d, J = 2.5 Hz, 1H), 8.58 (dd, J = 4.8, 1.6 Hz, 1H), 8.14 (dt, J = 7.9, 2.0 Hz, 1H), 8.02 (d, J = 8.6 Hz, 1H), 7.60 (d, J = 1.8 Hz, 1H), 7.49 (dd, J = 7.9, 4.7 Hz, 1H), 7.27 (dd, J = 8.6, 1.8 Hz, 1H), 6.55 (s, 2H), 5.94 (d, J = 1.6 Hz, 1H), 5.39 (s, 2H).

$^{13}C\{^1H\}$ NMR (126 MHz, DMSO-d_6) δ 157.0, 155.6, 148.6, 147.8, 140.9, 137.8, 135.6, 134.2, 125.3, 123.8, 121.8, 118.1, 110.7, 87.3.

HRMS (ESI$^+$) m/z calculated for $C_{14}H_{13}N_4$ $[M + H]^+$: 237.1135; found 237.1138.

IR (ATR) ν_{max}/cm^{-1} 3381 (m), 3308 (m), 1621 (s), 1562 (s), 1502 (m), 1475 (m), 1423 (s), 1398 (w), 1348 (m), 803 (s), 707 (m), 676 (w).

5-(trifluoromethyl)isoquinoline-1,3-diamine (2o)

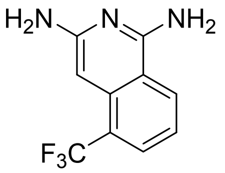

Chemical Formula: $C_{10}H_8F_3N_3$
Molecular Weight: 227.1902

Silica gel column chromatography eluent: MeOH/DCM = 1:50 to 1:20

Yield: 199 mg of a yellow solid, 88%

R_f 0.36 (MeOH/DCM=1/9)

^{1}H NMR (500 MHz, DMSO-d_6) δ 8.18 (d, J = 8.2 Hz, 1H), 7.70 (d, J = 6.7 Hz, 1H), 6.96 (t, J = 7.8 Hz, 1H), 6.77 (s, 2H), 5.99 (br q, J = 2.3 Hz, 1H), 5.76 (s, 2H).

^{13}C{^{1}H} NMR (126 MHz, DMSO-d_6) δ 158.0, 156.8, 136.5, 129.3, 128.5 (q, $J_{C\text{–}F}$ = 5.7 Hz), 125.1 (q, $J_{C\text{–}F}$ = 272.7 Hz), 119.8 (q, $J_{C\text{–}F}$ = 29.0 Hz), 116.9, 111.6, 83.0.

HRMS (ESI$^+$) m/z calculated for $C_{10}H_9F_3N_3$ [M + H]$^+$: 228.0743; found 228.0746.

IR (ATR) ν_{max}/cm^{-1} 3415 (m), 3320 (w), 3150 (w), 1620 (m), 1565 (w), 1505 (m), 1461 (w), 1423 (m), 1301 (s), 1240 (w), 1168 (w), 1112 (s), 807 (m), 755 (m), 727 (w).

4. Other experiments

Isoquinoline formation using 7N NH_3/MeOH

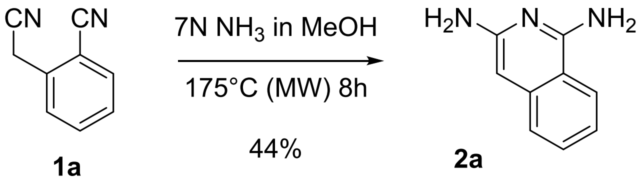

o-Cyanobenzyl cyanide (1.00 mmol, 143 mg) and 7 N NH_3/MeOH (0.8M, 1.25 mL) were placed in a microwave reaction tube. The mixture was heated under microwave irradiation in a sealed vessel at 175 °C for 2 h, and a small aliquot was taken and analyzed by ^{1}H NMR. The reaction was then continued at 175 °C for an additional 6 h. After cooling to room temperature, the volatiles were removed under reduced pressure. The crude product was purified by column chromatography on silica gel (MeOH/DCM = 1:50 to 1:10) to afford 1,3-diaminoisoquinoline (69.9 mg, 44%).

After 2 h from the start of the reaction, although a significant amount of starting material **1a** remained, the reaction already produced the target material **2a** along with byproducts. These results indicate that it is difficult to obtain **2a** in good yield using the method with 7 N NH_3 in methanol.

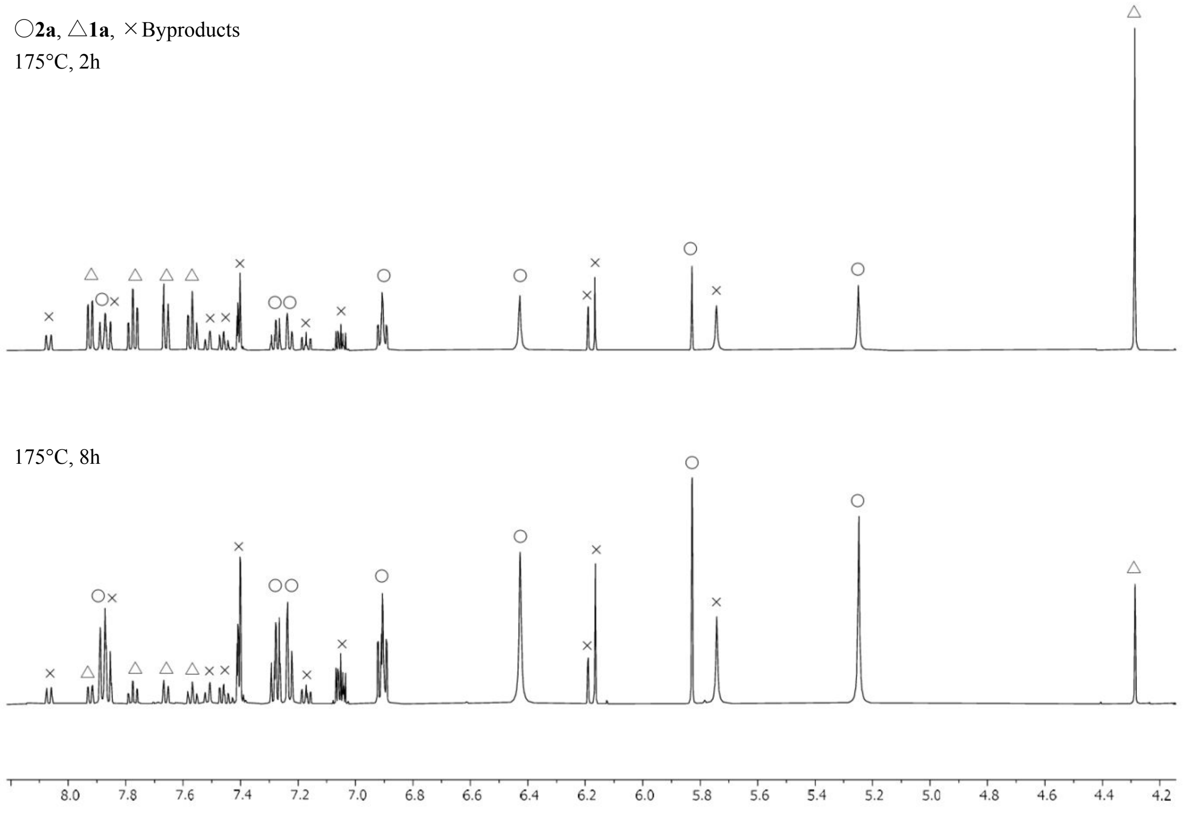

The reaction of *o*-cyanobenzyl cyanide and $NH_2OH{\cdot}HCl$ in MeOH [1]

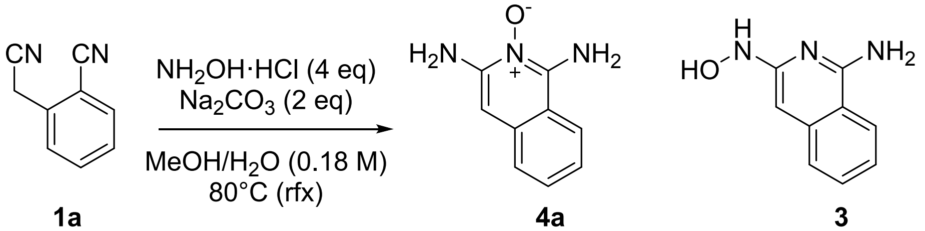

1a **4a** **3**

Our Result
72% of **4a**

Reported Result
78% of **3**

A mixture of *o*-Cyanobenzyl cyanide (1.0 equiv. 1.00 mmol, 142 mg), hydroxylamine hydrochloride (4.0 equiv. 4.00 mmol, 278 mg), and sodium carbonate (2.0 equiv. 2.00 mmol, 212 mg) were heated to reflux in MeOH (3.3 mL) /H_2O (2.2 mL) in an oil bath for 30 min. The reaction mixture was concentrated under reduced pressure. Water (1.5 mL) was added to the residue, and the mixture was heated to 100 °C with stirring. After cooling to room temperature, the precipitate was collected by filtration and washed with ice-cold water (1 mL) to afford 1,3-diaminoisoquinoline 2-oxide (126 mg of a brown solid, 72%). A portion of the compound was recrystallized by slow evaporation from an acetone/ethyl acetate solution to afford single crystals suitable for X-ray crystallography.

1,3-diaminoisoquinoline 2-oxide (4a)

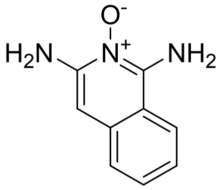

Chemical Formula: $C_9H_9N_3O$
Molecular Weight: 175.19

R_f 0.46 (MeOH/DCM=1/4)

^{1}H NMR (500 MHz, DMSO-d_6) δ 7.96 (d, *J* = 8.5 Hz, 1H), 7.55 (s, 2H), 7.41 (d, *J* = 7.4 Hz, 1H), 7.33 (ddd, *J* = 8.1, 6.7, 1.1 Hz, 1H), 7.10 (ddd, *J* = 8.1, 6.7, 1.2 Hz, 1H), 6.55 (s, 2H), 6.23 (s, 1H).

$^{13}C\{^1H\}$ NMR (126 MHz, DMSO-d_6) δ 146.9, 146.4, 131.4, 128.4, 124.2, 122.8, 121.1, 109.7, 86.5.

HRMS (ESI$^+$) m/z calculated for $C_9H_{10}N_3O$ $[M + H]^+$: 176.0818; found 176.0820

IR (ATR) ν_{max}/cm^{-1} 3424 (w), 3046 (m), 1607 (s), 1507 (w), 1469 (m), 1249 (w), 1191 (m), 1106 (w), 1042 (w), 891 (w), 865 (w), 786 (w), 708 (w).

Comparison of the literature[1] and our ^{1}H NMR data

ppm, coupling, integration	
Literature Data (**3**)	Our Data (**4a**)
8.04, br-d, 1H	7.96, d, 1H
7.67, br-s, 2H	7.55, s, 2H
7.45-6.95, complex, 3H	7.41, d, 1H
	7.33, ddd, 1H
	7.10,ddd, 1H
6.64, broadened, 2H	6.55, s, 2H
6.32, s, 1H	6.23, s, 1H

3.58, broadened (H_2O)	3.35, s (H_2O)

For comparison, the literature ^{1}H NMR data, originally reported on the τ scale, were converted to ppm using δ = 10 − τ. The converted values are in reasonable agreement with our data, supporting the assignment of the same structure.

Solvent screening for isoquinoline *N*-oxide formation (NMR experiment)

o-Cyanobenzyl cyanide (1.0 equiv, 0.500 mmol, 71.1 mg) and 1,3,5-trimethoxybenzene (0.11 equiv, 0.0556 mmol, 9.3 mg) as an internal standard were dissolved in the appropriate solvent (0.5 M, 1.0 mL), and 50% aqueous NH_2OH solution (2.0 equiv, 1.00 mmol, 61.3 µL) was added. The reaction mixture was heated at 60 °C in an oil bath. At 1, 3, and 6 h after the start of the reaction, small aliquots were withdrawn from the reaction mixture and analyzed by ^{1}H NMR. The reaction progress was evaluated from the ratio of the integral values of the product signals to those of the internal standard.

Acetylation of 1,3-diaminoisoquinoline

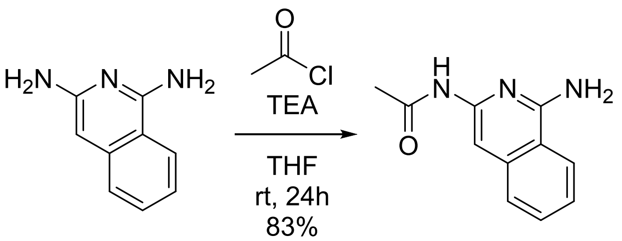

2a **5**

1,3-Diaminoisoquinoline (**2a**) (1.0 equiv. 0.400 mmol, 63.7 mg) and triethylamine (2.0 equiv. 0.800 mmol, 111 µL) were dissolved in ethanol (4.0 mL), and acetyl chloride (1.1 equiv. 0.440 mmol, 31.3 µL) was added at room temperature. The reaction mixture was stirred for 24 h, then silica gel (1 g) was added and the volatiles were removed under reduced pressure. The residue was purified by column chromatography on silica gel (EtOAc / Cyclohexane = 2/1 to 3/1) to afford **5** (66.6 mg of a beige solid, 83%). A portion of **5** was recrystallized by slow evaporation from an acetone/heptane solution to afford single crystals suitable for X-ray crystallography.

N-(3-aminoisoquinolin-1-yl)acetamide (5)

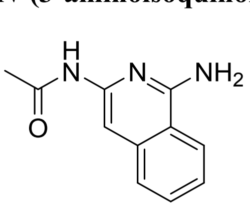

Chemical Formula: $C_{11}H_{11}N_3O$
Molecular Weight: 201.23

R_f 0.50 (MeOH/DCM=1/9)

^{1}H NMR (500 MHz, DMSO-d_6) δ 9.81 (s, 1H), 8.12 (dq, *J* = 8.3, 0.9 Hz, 1H), 7.60 (s, 1H), 7.58 (dd, *J* = 8.2, 1.2 Hz, 1H), 7.51 (ddd, *J* = 8.1, 6.8, 1.2 Hz, 1H), 7.28 (ddd, *J* = 8.2, 6.8, 1.3 Hz, 1H), 6.66 (s, 2H), 2.07 (s, 3H).

^{13}C{^{1}H} NMR (126 MHz, DMSO-d_6) δ 168.7, 156.4, 145.8, 139.0, 130.0, 126.2, 123.9, 123.4, 115.0, 96.3, 24.0.

HRMS (ESI$^+$) m/z calculated for $C_{11}H_{12}N_3O$ [M + H]$^+$: 202.0975; found 202.0978

IR (ATR) ν_{max}/cm^{-1} 3394 (w), 3315 (m), 3229 (w), 1679 (m), 1638 (m), 1533 (s), 1428 (s), 1383 (m), 1340 (m), 1270 (m), 1163 (m), 828 (m), 748 (m), 687 (w).

5. Crystallographic details for 4a and 5

1,3-diaminoisoquinoline 2-oxide (4a)

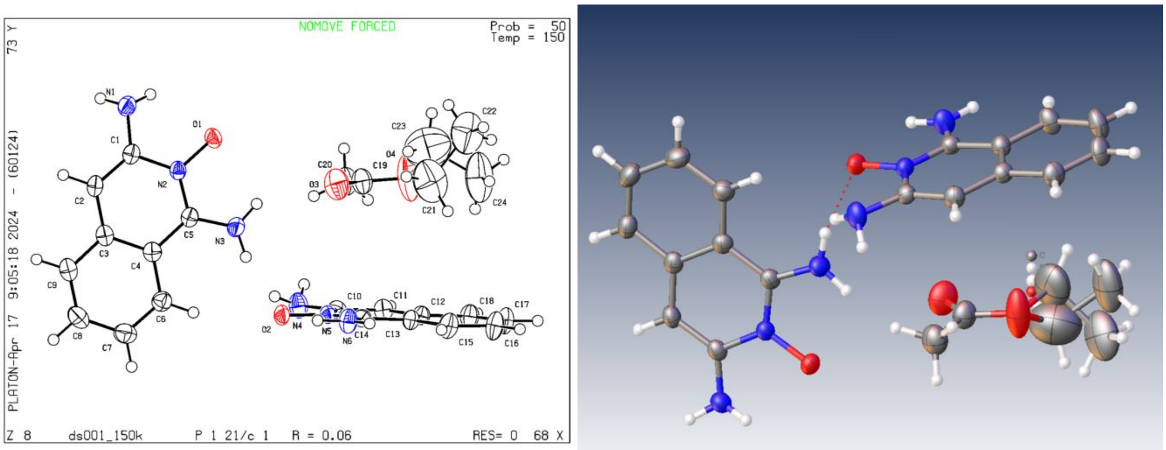

ORTEP drawing of the structure with ellipsoids shown at the 50% probability level.

Table 1 Crystal data and structure refinement for DS001_150K.

Identification code	DS001_150K
Empirical formula	$C_{11.25}H_{13.5}N_3O_2$
Formula weight	222.75
Temperature/K	150
Crystal system	monoclinic
Space group	$P2_1/c$
a/Å	10.2642(2)
b/Å	13.0171(2)
c/Å	17.4736(3)
α/°	90
β/°	106.2430(10)
γ/°	90
Volume/Å^3	2241.46(7)
Z	8
ρ_{calc}g/cm^3	1.320
μ/mm^{-1}	0.488
F(000)	944.0
Crystal size/mm^3	0.21 × 0.197 × 0.18
Radiation	GaKα (λ = 1.34143)
2Θ range for data collection/°	7.478 to 111.21
Index ranges	$-12 \leq h \leq 10$, $-15 \leq k \leq 15$, $-21 \leq l \leq 20$
Reflections collected	30617
Independent reflections	4334 [R_{int} = 0.0267, R_{sigma} = 0.0148]
Data/restraints/parameters	4334/74/312
Goodness-of-fit on F^2	1.053
Final R indexes [I>=2σ (I)]	R_1 = 0.0614, wR_2 = 0.1699
Final R indexes [all data]	R_1 = 0.0625, wR_2 = 0.1711
Largest diff. peak/hole / e Å^{-3}	0.52/-0.56

Table 2 Fractional Atomic Coordinates (×10[4]) and Equivalent Isotropic Displacement Parameters (Å[2]×10[3]) for DS001_150K. U_{eq} is defined as 1/3 of the trace of the orthogonalised U_{IJ} tensor.

Atom	*x*	*y*	*z*	U(eq)
O1	2725.7(13)	3909.5(9)	6687.6(7)	29.0(3)
O2	2321.9(14)	4286.4(9)	3590.4(8)	30.7(3)
N2	2436.4(14)	3000.3(11)	6304.2(9)	24.6(3)
N5	2209.9(14)	5293.6(11)	3401.7(8)	24.1(3)
N6	4422.7(16)	5222.7(12)	3362.2(10)	33.3(4)
N3	2369.4(17)	3879.2(12)	5169.0(9)	33.0(4)
N1	2623.3(17)	2299.2(13)	7541.2(9)	32.8(4)
O3	3507.5(18)	6076.1(13)	5504.3(12)	57.3(5)
N4	23.4(17)	5191.0(13)	3523.6(11)	37.1(4)
C1	2382.3(17)	2124.8(13)	6750.0(11)	26.1(4)
C14	3290.9(17)	5782.7(13)	3273.0(10)	25.0(4)
C5	2252.5(17)	2980.3(13)	5505.5(10)	25.7(4)
C12	1926.9(17)	7345.3(13)	2989.0(10)	26.6(4)
C10	989.3(17)	5788.9(14)	3358.6(10)	26.4(4)
C4	1929.7(17)	2021.2(13)	5092.4(11)	26.9(4)
C3	1902.6(17)	1120.7(13)	5545.4(11)	27.3(4)
C2	2129.4(18)	1190.4(13)	6379.3(11)	28.5(4)
C11	853.9(18)	6805.2(14)	3160.3(11)	29.0(4)
C13	3165.1(17)	6834.5(13)	3039.9(10)	26.1(4)
O4	3132(2)	7734.0(14)	5287.6(19)	86.7(8)
C9	1640.8(19)	170.1(14)	5137.0(12)	33.4(4)
C6	1628(2)	1957.6(15)	4253.3(11)	32.8(4)
C18	1820(2)	8398.2(15)	2770.6(12)	33.7(4)
C7	1348(2)	1023.8(17)	3878.5(12)	39.0(5)
C8	1375(2)	125.1(16)	4327.7(13)	39.2(5)
C17	2884(2)	8907.5(15)	2618.9(13)	39.7(5)
C15	4241(2)	7372.3(16)	2863.0(13)	36.5(5)
C19	2758(2)	6795.0(16)	5396.5(14)	41.8(5)
C16	4106(2)	8388.9(17)	2664.7(14)	42.8(5)
C20	1297(2)	6750.6(19)	5367.1(16)	50.4(6)
C23	4639(13)	8024(12)	5787(8)	151(5)
C24	4802(8)	8895(6)	5113(8)	107(3)
C22	4926(7)	8905(7)	5868(6)	101(3)
C21	4541(4)	7876(4)	5241(4)	106.3(15)

Table 3 Anisotropic Displacement Parameters ($Å^2×10^3$) for DS001_150K. The Anisotropic displacement factor exponent takes the form: $-2\pi^2[h^2a^{*2}U_{11}+2hka^*b^*U_{12}+…]$.

Atom	U_{11}	U_{22}	U_{33}	U_{23}	U_{13}	U_{12}
O1	34.7(7)	20.1(6)	34.9(7)	-2.6(5)	14.0(5)	0.7(5)
O2	44.3(8)	18.1(6)	33.7(7)	1.3(5)	17.5(6)	1.1(5)
N2	26.2(7)	20.2(7)	30.2(7)	0.4(5)	12.2(6)	1.9(5)
N5	28.5(8)	18.7(7)	26.6(7)	0.1(5)	10.1(6)	0.4(5)
N6	30.4(8)	25.2(8)	48.0(9)	6.2(7)	17.2(7)	7.2(6)
N3	46.6(9)	24.4(8)	30.5(8)	2.9(6)	14.9(7)	1.3(6)
N1	42.2(9)	28.0(8)	32.4(8)	2.3(6)	17.5(7)	-0.3(7)
O3	50.1(10)	41.5(9)	77.7(12)	5.8(8)	13.6(9)	14.6(7)
N4	30.6(8)	30.6(9)	53.9(10)	1.5(7)	17.7(7)	-3.8(6)
C1	23.4(8)	25.4(9)	32.1(9)	3.9(7)	12.2(7)	2.8(6)
C14	25.8(8)	24.0(8)	26.1(8)	-0.2(6)	8.7(6)	2.2(6)
C5	24.6(8)	23.9(8)	31.0(9)	2.8(7)	11.7(7)	4.1(6)
C12	26.3(8)	25.0(9)	28.4(8)	1.4(7)	7.5(7)	2.4(7)
C10	23.4(8)	28.1(9)	28.3(8)	-2.5(7)	7.9(7)	-2.6(7)
C4	23.6(8)	26.8(9)	32.5(9)	-0.1(7)	11.6(7)	2.8(6)
C3	21.8(8)	25.1(9)	37.0(9)	0.0(7)	11.7(7)	0.9(6)
C2	29.1(9)	22.4(8)	35.9(9)	5.1(7)	12.5(7)	0.8(7)
C11	23.1(8)	28.2(9)	36.4(9)	0.9(7)	9.4(7)	2.9(7)
C13	24.5(8)	24.1(8)	30.2(9)	3.3(7)	8.3(7)	1.2(6)
O4	46.8(10)	39.4(10)	183(3)	16.2(12)	46.5(13)	2.7(8)
C9	32.4(10)	25.4(9)	44.5(11)	-1.2(8)	14.1(8)	-1.7(7)
C6	34.8(10)	32.5(10)	32.9(9)	1.1(7)	12.6(8)	2.3(7)
C18	32.3(9)	26.6(9)	42.0(10)	5.9(8)	10.4(8)	7.1(7)
C7	40.3(11)	43.0(11)	34.6(10)	-6.7(8)	12.0(8)	-3.3(9)
C8	39.6(11)	33.6(10)	46.4(11)	-11.4(8)	15.5(9)	-5.8(8)
C17	39.9(11)	26.4(9)	52.7(12)	12.7(8)	12.9(9)	2.1(8)
C15	27.5(9)	35.0(10)	49.9(11)	9.5(8)	15.7(8)	2.7(8)
C19	39.2(11)	32.0(10)	53.8(13)	2.7(9)	12.5(9)	5.0(8)
C16	34.9(10)	35.8(11)	61.1(13)	15.9(10)	18.7(9)	-2.4(8)
C20	41.3(12)	48.0(13)	64.5(15)	3.0(11)	19.2(11)	-1.5(10)
C23	148(8)	180(9)	133(7)	-2(7)	53(7)	12(7)
C24	54(4)	74(4)	204(10)	25(5)	55(5)	-2(3)
C22	46(3)	109(6)	133(7)	24(5)	2(4)	-17(4)
C21	68(2)	105(3)	162(4)	-10(3)	60(3)	-17(2)

Table 4 Bond Lengths for DS001_150K.

Atom	Atom	Length/Å	Atom	Atom	Length/Å
O1	N2	1.3514(18)	C4	C3	1.419(2)
O2	N5	1.3489(18)	C4	C6	1.414(3)
N2	C1	1.390(2)	C3	C2	1.413(3)
N2	C5	1.355(2)	C3	C9	1.416(3)
N5	C14	1.352(2)	C13	C15	1.413(3)
N5	C10	1.392(2)	O4	C19	1.311(3)
N6	C14	1.343(2)	O4	C23	1.595(13)
N3	C5	1.330(2)	O4	C21	1.483(4)
N1	C1	1.354(2)	C9	C8	1.365(3)
O3	C19	1.192(3)	C6	C7	1.372(3)
N4	C10	1.353(2)	C18	C17	1.366(3)
C1	C2	1.368(3)	C7	C8	1.405(3)
C14	C13	1.424(2)	C17	C16	1.407(3)
C5	C4	1.434(2)	C15	C16	1.365(3)
C12	C11	1.408(2)	C19	C20	1.487(3)
C12	C13	1.415(2)	C23	C24	1.677(13)
C12	C18	1.419(3)	C22	C21	1.704(10)
C10	C11	1.365(3)			

Table 5 Bond Angles for DS001_150K.

Atom	Atom	Atom	Angle/°	Atom	Atom	Atom	Angle/°
O1	N2	C1	118.55(14)	C2	C3	C4	119.93(16)
O1	N2	C5	118.38(14)	C2	C3	C9	121.95(17)
C5	N2	C1	123.04(15)	C9	C3	C4	118.12(17)
O2	N5	C14	118.73(14)	C1	C2	C3	120.01(16)
O2	N5	C10	118.50(14)	C10	C11	C12	120.53(16)
C14	N5	C10	122.76(15)	C12	C13	C14	118.55(15)
N1	C1	N2	114.22(16)	C15	C13	C14	121.68(16)
N1	C1	C2	126.08(16)	C15	C13	C12	119.78(16)
C2	C1	N2	119.69(16)	C19	O4	C23	114.8(6)
N5	C14	C13	119.18(15)	C19	O4	C21	117.0(3)
N6	C14	N5	116.66(15)	C8	C9	C3	120.96(18)
N6	C14	C13	124.15(16)	C7	C6	C4	120.17(18)
N2	C5	C4	118.77(15)	C17	C18	C12	121.02(18)
N3	C5	N2	115.76(15)	C6	C7	C8	120.15(18)
N3	C5	C4	125.46(16)	C9	C8	C7	120.68(18)
C11	C12	C13	119.62(16)	C18	C17	C16	120.32(18)
C11	C12	C18	122.24(16)	C16	C15	C13	120.40(18)
C13	C12	C18	118.13(16)	O3	C19	O4	123.6(2)
N4	C10	N5	115.08(16)	O3	C19	C20	125.1(2)
N4	C10	C11	125.65(17)	O4	C19	C20	111.35(19)
C11	C10	N5	119.26(15)	C15	C16	C17	120.32(18)
C3	C4	C5	118.44(16)	O4	C23	C24	92.8(7)
C6	C4	C5	121.73(16)	O4	C21	C22	96.8(4)
C6	C4	C3	119.83(17)				

Table 6 Torsion Angles for DS001_150K.

A	B	C	D	Angle/°	A	B	C	D	Angle/°
O1	N2	C1	N1	0.6(2)	C12	C13	C15	C16	1.8(3)
O1	N2	C1	C2	-178.15(15)	C12	C18	C17	C16	1.0(3)
O1	N2	C5	N3	-0.4(2)	C10	N5	C14	N6	177.62(15)
O1	N2	C5	C4	-179.29(14)	C10	N5	C14	C13	-3.6(2)
O2	N5	C14	N6	-1.5(2)	C4	C3	C2	C1	-0.4(3)
O2	N5	C14	C13	177.32(14)	C4	C3	C9	C8	-2.2(3)
O2	N5	C10	N4	0.7(2)	C4	C6	C7	C8	-0.8(3)
O2	N5	C10	C11	-179.32(15)	C3	C4	C6	C7	-1.9(3)
N2	C1	C2	C3	-1.0(3)	C3	C9	C8	C7	-0.3(3)
N2	C5	C4	C3	-4.0(2)	C2	C3	C9	C8	177.57(18)
N2	C5	C4	C6	175.33(16)	C11	C12	C13	C14	-0.6(3)
N5	C14	C13	C12	3.0(2)	C11	C12	C13	C15	179.56(17)
N5	C14	C13	C15	-177.15(17)	C11	C12	C18	C17	179.03(19)
N5	C10	C11	C12	1.0(3)	C13	C12	C11	C10	-1.4(3)
N6	C14	C13	C12	-178.27(17)	C13	C12	C18	C17	-0.4(3)
N6	C14	C13	C15	1.5(3)	C13	C15	C16	C17	-1.3(3)
N3	C5	C4	C3	177.23(16)	C9	C3	C2	C1	179.84(16)
N3	C5	C4	C6	-3.4(3)	C6	C4	C3	C2	-176.49(16)
N1	C1	C2	C3	-179.60(16)	C6	C4	C3	C9	3.3(2)
N4	C10	C11	C12	-179.02(18)	C6	C7	C8	C9	1.9(3)
C1	N2	C5	N3	-178.37(15)	C18	C12	C11	C10	179.18(17)
C1	N2	C5	C4	2.7(2)	C18	C12	C13	C14	178.85(16)
C14	N5	C10	N4	-178.42(16)	C18	C12	C13	C15	-1.0(3)
C14	N5	C10	C11	1.6(3)	C18	C17	C16	C15	-0.2(4)
C14	C13	C15	C16	-177.99(19)	C19	O4	C23	C24	152.4(6)
C5	N2	C1	N1	178.53(15)	C19	O4	C21	C22	-133.6(5)
C5	N2	C1	C2	-0.2(3)	C23	O4	C19	O3	-35.3(7)
C5	C4	C3	C2	2.9(2)	C23	O4	C19	C20	144.7(6)
C5	C4	C3	C9	-177.33(16)	C21	O4	C19	O3	4.5(5)
C5	C4	C6	C7	178.80(17)	C21	O4	C19	C20	-175.5(4)

Table 7 Hydrogen Atom Coordinates (Å×10^4) and Isotropic Displacement Parameters (Å^2×10^3) for DS001_150K.

Atom	*x*	*y*	*z*	U(eq)
H6A	4433.83	4571.07	3498.09	40
H6B	5153.09	5507.31	3284.76	40
H3A	2555.17	4437.69	5463.28	40
H3B	2260.9	3915.57	4651.99	40
H1A	2398.42	2922.59	7614.59	39
H1B	2148.31	1883.01	7738.58	39
H4A	403.05	4662.1	3788.75	45
H4B	-386.34	5538.46	3805.74	45
H2	2106.44	588.5	6682.65	34
H11	28.86	7150.85	3137.69	35
H9	1650.12	-445.31	5430.79	40
H6	1619.51	2562.77	3948.05	39
H18	995.35	8754.47	2729.28	40
H7	1135.05	985.18	3314.09	47
H8	1206.89	-520.72	4065.25	47
H17	2798.78	9615.47	2481.81	48
H15	5063.1	7024.38	2882.13	44
H16	4841.45	8747.91	2557.02	51
H20A	961.95	6047.38	5245.8	76
H20B	780.26	7216.5	4951.84	76
H20C	1186.31	6958.65	5884.27	76
H23A	5282.84	7443.63	5844.9	181
H23B	4689.01	8328.33	6313.44	181
H24A	4174.41	8728.86	4592.4	160
H24B	5735.58	8885.59	5072.48	160
H24C	4591.89	9579.82	5278.65	160
H22A	4358.24	9490.57	5627.98	151
H22B	5884.53	9084.5	5958.47	151
H22C	4756.97	8730.25	6377.17	151
H21A	4578.3	8044.41	4695.24	128
H21B	5124	7274.52	5446.35	128

Table 8 Atomic Occupancy for DS001_150K.

Atom	*Occupancy*	Atom	*Occupancy*	Atom	*Occupancy*
C23	0.5	H23A	0.5	H23B	0.5
C24	0.5	H24A	0.5	H24B	0.5
H24C	0.5	C22	0.5	H22A	0.5
H22B	0.5	H22C	0.5		

Crystal structure determination of [DS001_150K]

Crystal Data for C11.25H13.5N3O2 (M =222.75 g/mol): monoclinic, space group P21/c (no. 14), a = 10.2642(2) Å, b = 13.0171(2) Å, c = 17.4736(3) Å, β = 106.2430(10)°, V = 2241.46(7) Å3, Z = 8, T = 150 K, μ(GaKα) = 0.488 mm-1, Dcalc = 1.320 g/cm3, 30617 reflections measured (7.478° ≤ 2Θ ≤ 111.21°), 4334 unique (Rint = 0.0267, Rsigma = 0.0148) which were used in all calculations. The final R1 was 0.0614 (I > 2σ(I)) and wR2 was 0.1711 (all data).

***N*-(3-aminoisoquinolin-1-yl)acetamide (5)**

DS3-076-2_150K

Submitted by: **Daisuke Sakamoto**
University of Basel
Solved by: **Alessandro Prescimone**
Sample ID: **DS3-076-2**

***R_1*=4.62%**

Crystal Data and Experimental

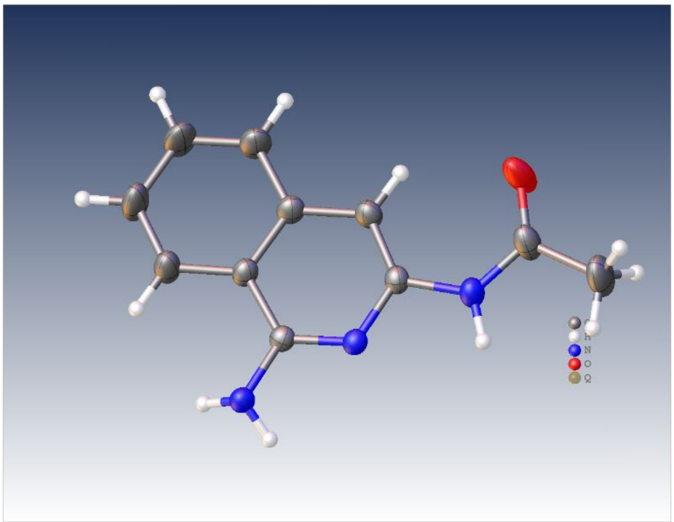

Experimental. Single colourless block-shaped crystals of **DS3-076-2_150K** were used as supplied. A suitable crystal with dimensions 0.30 × 0.23 × 0.20 mm was selected and mounted on a STOE STADIVARI Cu diffractometer. The crystal was kept at a steady T = 150 K during data collection. The structure was solved with the ShelXT 2018/2 (Sheldrick, 2018) solution program using dual methods and by using Olex2 1.5 (Dolomanov et al., 2009) as the graphical interface. The model was refined with ShelXL 2019/3 (Sheldrick, 2015) using full matrix least squares minimisation on $|F|^2$.

Crystal Data. $C_{11}H_{11}N_3O$, M_r = 201.23, tetragonal, $I4_1/a$ (No. 88), a = 17.0442(4) Å, b = 17.0442(4) Å, c = 14.9648(4) Å, $\alpha = \beta = \gamma = 90^{\circ}$, V = 4347.3(2) Å^3, T = 150 K, Z = 16, Z' = 1, μ(Cu K$_{\alpha}$) = 0.670, 39733 reflections measured, 2229 unique (R_{int} = 0.0227) which were used in all calculations. The final wR_2 was 0.1291 (all data) and R_1 was 0.0462 (I≥2σ(I)).

**Compound**	**DS3-076-2_150K**
Formula	$C_{11}H_{11}N_3O$
$D_{calc.}$/ g cm^{-3}	1.230
μ/mm^{-1}	0.670
Formula Weight	201.23
Colour	colourless
Shape	block-shaped
Size/mm	0.30×0.23×0.20
T/K	150
Crystal System	tetragonal
Space Group	$I4_1/a$
a/Å	17.0442(4)
b/Å	17.0442(4)
c/Å	14.9648(4)
α/°	90
β/°	90
γ/°	90
V/Å^3	4347.3(2)
Z	16
Z'	1
Wavelength/Å	1.54186
Radiation type	Cu K_α
Θ_{min}/°	3.931
Θ_{max}/°	74.800
Measured Refl's.	39733
Indep't Refl's	2229
Refl's I≥2σ(I)	1977
R_{int}	0.0227
Parameters	142
Restraints	0
Largest Peak/eÅ^3	0.265
Deepest Hole/eÅ^3	-0.337
GooF	1.033
wR_2 (all data)	0.1291
wR_2	0.1240
R_1 (all data)	0.0512
R_1	0.0462

Structure Quality Indicators

**Reflections:**	d min (CuKα) 2Θ=149.6° 0.80	I/σ(I) 151.5	Rint m=18.27 2.27%	Full 135.4° 100
**Refinement:**	Shift 0.000	Max Peak 0.3	Min Peak -0.3	GooF 1.033

A colourless block-shaped crystal with dimensions 0.30 × 0.23 × 0.20 mm was mounted. Data were collected using a STOE STADIVARI Cu diffractometer equipped with an Oxford Cryosystems low-temperature device operating at T = 150 K.

Data were measured using rotation method, ω scans with Cu K_{α} radiation. The diffraction pattern was indexed and the total number of runs and images was based on the strategy calculation from the program X-Area Pilatus3_SV 1.31.170.0 (STOE, 2020) . The maximum resolution achieved was Θ = 74.800° (0.80 Å).

The unit cell was refined using X-Area Pilatus3_SV 1.31.170.0 (STOE, 2020) on 50026 reflections, 126% of the observed reflections.

Data reduction, scaling and absorption corrections were performed using X-Area Pilatus3_SV 1.31.170.0 (STOE, 2020). The final completeness is 100.00 % out to 74.800° in Θ. A multi-scan absorption correction was performed using STOE. The absorption coefficient μ of this material is 0.670 mm^{-1} at this wavelength (λ = 1.54186Å) and the minimum and maximum transmissions are 0.416 and 0.741.

The structure was solved in the space group $I4_1/a$ (# 88) by ShelXT 2018/2 (Sheldrick, 2018) using dual methods. It was refined by full matrix least squares minimisation on $|F|^2$ using version 2019/3 of ShelXL 2019/3 (Sheldrick, 2015). All non-hydrogen atoms were refined anisotropically.

Most hydrogen atom positions were calculated geometrically and refined using the riding model, but some hydrogen atoms were refined freely.

_exptl_absorpt_process_details: STOE X-Red32, absorption correction by Gaussian integration, analogous toP. Coppens in: F. R. Ahmed (Editor), "Crystallographic Computing", Munksgaard,Copenhagen (1970), 255 - 270. Afterwards scaling of reflection intensities wasperformed within STOE LANA. J. Koziskova, F. Hahn, J. Richter, J. Kozisek, ActaChimica Slovaca, vol. 9, no. 2, 2016, pp. 136 - 140.Finally a spherical absorption correction was done within STOE LANA.

There is a single formula unit in the asymmetric unit, which is represented by the reported sum formula. In other words: Z is 16 and Z' is 1. The moiety formula is C11 H11 N3 O.

Data Plots: Diffraction Data

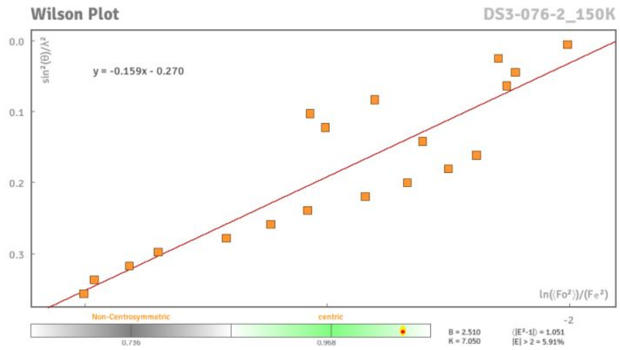

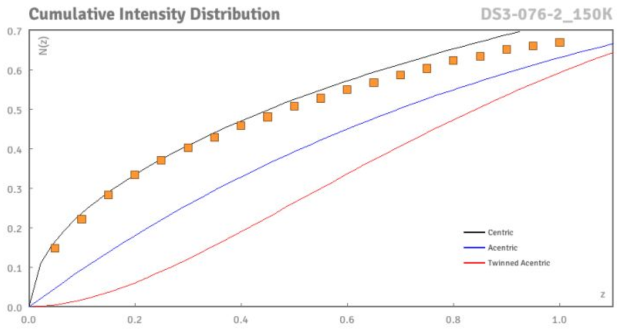

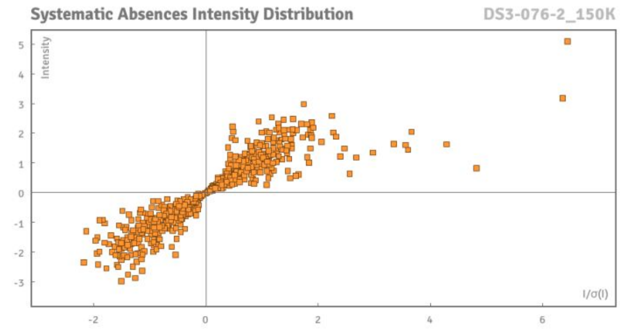

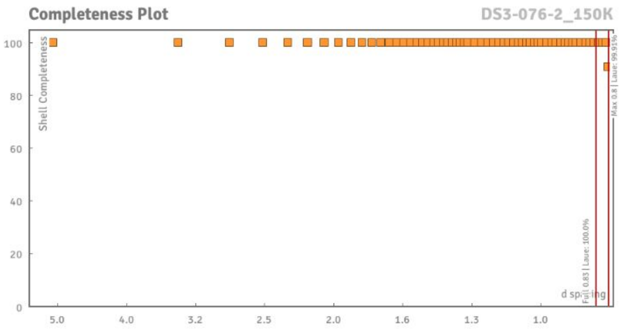

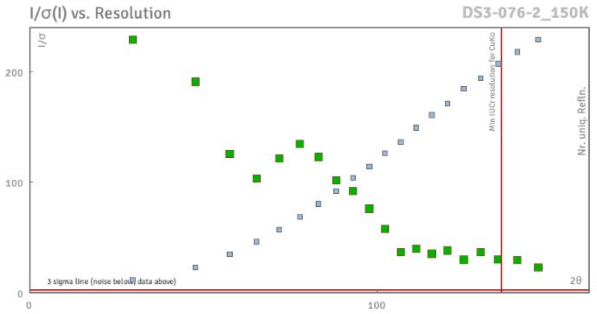

Data Plots: Refinement and Data

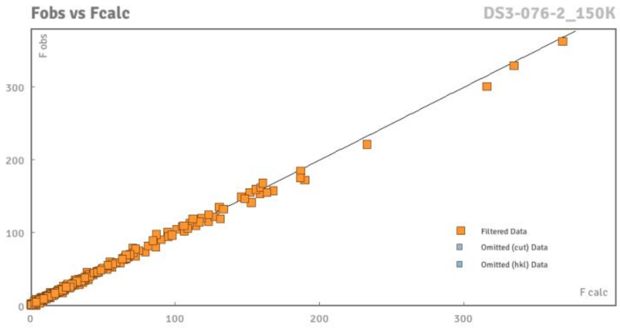

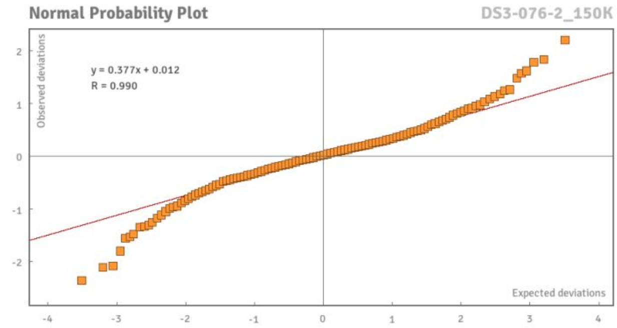

Reflection Statistics

Total reflections (after filtering)	40724	Unique reflections	2229
Completeness	0.999	Mean I/σ	82.79
hkl_{max} collected	(18, 21, 18)	hkl_{min} collected	(-21, -21, -14)
hkl_{max} used	(15, 21, 18)	hkl_{min} used	(-14, 0, 0)

Lim d_{max} collected	100.0	Lim d_{min} collected	0.77
d_{max} used	12.05	d_{min} used	0.8
Friedel pairs	3736	Friedel pairs merged	1
Inconsistent equivalents	32	R_{int}	0.0227
R_{sigma}	0.0066	Intensity transformed	0
Omitted reflections	0	Omitted by user (OMIT hkl)	0
Multiplicity	(2947, 2517, 1951, 1561, 1183, 830, 566, 313, 202, 95, 26, 16, 3)	Maximum multiplicity	44
Removed systematic absences	991	Filtered off (Shel/OMIT)	0

Table 1: Fractional Atomic Coordinates (×10^4) and Equivalent Isotropic Displacement Parameters (Å^2×10^3) for **DS3-076-2_150K.** U_{eq} is defined as 1/3 of the trace of the orthogonalised U_{ij}.

Atom	x	y	z	U_{eq}
N2	5786.2(6)	3582.5(6)	4449.6(7)	23.0(3)
N3	5945.5(7)	2340.5(6)	4992.5(8)	26.7(3)
N1	5685.2(8)	4756.7(7)	3728.1(8)	30.3(3)
O1	6157.2(10)	5985.2(6)	3973.2(8)	55.5(4)
C3	5856.7(8)	4377.8(8)	4540.2(9)	24.3(3)
C7	5970.5(7)	3129.8(7)	5133.4(9)	22.5(3)
C4	6078.7(8)	4744.9(8)	5308.4(10)	28.7(3)
C6	6231.4(8)	3441.0(8)	5975.7(9)	26.9(3)
C5	6276.0(9)	4267.8(8)	6050.6(9)	29.4(3)
C1	5847.9(11)	5501.1(9)	3479.3(10)	38.3(4)
C8	6452.0(11)	2965.5(9)	6704.0(10)	40.6(4)
C11	6539.2(12)	4590.0(9)	6869.3(11)	44.6(4)
C9	6706.5(15)	3297.5(11)	7482.9(12)	56.9(6)
C10	6750.3(15)	4118.0(11)	7564.0(12)	57.8(6)
C2	5627.1(15)	5693.6(11)	2530.1(12)	57.1(6)

Table 2: Anisotropic Displacement Parameters (×10^4) for **DS3-076-2_150K.** The anisotropic displacement factor exponent takes the form: $-2\pi^2[h^2a^{*2} \times U_{11} + ... + 2hka^* \times b^* \times U_{12}]$

Atom	U_{11}	U_{22}	U_{33}	U_{23}	U_{13}	U_{12}
N2	25.0(5)	23.6(5)	20.5(5)	-0.6(4)	-1.2(4)	1.5(4)
N3	36.3(6)	22.8(5)	21.0(6)	-0.9(4)	-4.5(5)	-1.0(4)
N1	43.0(7)	25.7(6)	22.1(6)	2.0(5)	-2.6(5)	2.6(5)
O1	110.0(12)	24.1(6)	32.5(7)	-1.6(5)	0.6(7)	-4.6(6)
C3	26.5(6)	24.0(6)	22.5(7)	1.4(5)	1.3(5)	3.4(5)
C7	23.1(6)	23.6(6)	20.7(6)	-1.7(5)	1.1(5)	2.3(5)
C4	38.8(8)	21.9(6)	25.6(7)	-2.4(5)	0.6(6)	3.0(5)
C6	33.9(7)	26.5(7)	20.2(6)	-2.2(5)	-1.4(5)	3.9(5)
C5	38.5(8)	26.8(7)	22.9(7)	-4.8(5)	-1.4(6)	3.6(5)
C1	62.2(10)	26.4(7)	26.4(8)	2.0(6)	5.1(7)	6.9(7)
C8	68.1(11)	28.7(7)	25.1(8)	-0.9(6)	-9.8(7)	6.6(7)
C11	74.5(12)	30.4(8)	28.9(8)	-8.7(6)	-11.4(8)	3.9(7)
C9	103.5(17)	39.3(9)	27.8(9)	-1.3(7)	-23.2(9)	13.0(10)
C10	103.4(17)	41.7(10)	28.4(9)	-10.9(7)	-24.3(9)	7.6(10)
C2	100.5(17)	39.5(9)	31.3(9)	11.9(7)	-4.5(9)	1.6(10)

Table 3: Bond Lengths in Å for **DS3-076-2_150K.**

Atom	Atom	Length/Å
N2	C3	1.3676(17)
N2	C7	1.3194(17)
N3	C7	1.3624(17)
N1	C3	1.4071(17)
N1	C1	1.3510(19)
O1	C1	1.227(2)
C3	C4	1.3624(19)
C7	C6	1.4381(18)
C4	C5	1.417(2)
C6	C5	1.4157(19)
C6	C8	1.409(2)
C5	C11	1.416(2)
C1	C2	1.506(2)
C8	C9	1.366(2)
C11	C10	1.363(2)
C9	C10	1.406(3)

Table 4: Bond Angles in ° for **DS3-076-2_150K**.

Atom	Atom	Atom	Angle/°
C7	N2	C3	118.80(11)
C1	N1	C3	128.78(13)
N2	C3	N1	110.55(11)
C4	C3	N2	124.28(12)
C4	C3	N1	125.15(12)
N2	C7	N3	116.74(12)
N2	C7	C6	122.53(12)
N3	C7	C6	120.63(12)
C3	C4	C5	117.63(12)
C5	C6	C7	116.95(12)
C8	C6	C7	123.24(13)
C8	C6	C5	119.80(13)
C6	C5	C4	119.76(12)
C11	C5	C4	122.08(13)
C11	C5	C6	118.14(13)
N1	C1	C2	114.41(15)
O1	C1	N1	123.64(15)
O1	C1	C2	121.95(15)
C9	C8	C6	120.40(15)
C10	C11	C5	120.99(15)
C8	C9	C10	120.18(15)
C11	C10	C9	120.48(15)

Table 5: Torsion Angles in ° for **DS3-076-2_150K**.

Atom	Atom	Atom	Atom	Angle/°
N2	C3	C4	C5	2.4(2)
N2	C7	C6	C5	0.7(2)
N2	C7	C6	C8	-178.11(14)
N3	C7	C6	C5	176.97(12)
N3	C7	C6	C8	-1.8(2)
N1	C3	C4	C5	-176.17(13)
C3	N2	C7	N3	-175.41(11)

Atom	Atom	Atom	Atom	Angle/˚
C3	N2	C7	C6	0.99(19)
C3	N1	C1	O1	-3.4(3)
C3	N1	C1	C2	175.90(16)
C3	C4	C5	C6	-0.5(2)
C3	C4	C5	C11	177.82(15)
C7	N2	C3	N1	176.08(11)
C7	N2	C3	C4	-2.65(19)
C7	C6	C5	C4	-0.9(2)
C7	C6	C5	C11	-179.32(14)
C7	C6	C8	C9	178.98(18)
C4	C5	C11	C10	-177.81(19)
C6	C5	C11	C10	0.5(3)
C6	C8	C9	C10	0.0(3)
C5	C6	C8	C9	0.2(3)
C5	C11	C10	C9	-0.4(4)
C1	N1	C3	N2	-164.02(14)
C1	N1	C3	C4	14.7(2)
C8	C6	C5	C4	177.93(15)
C8	C6	C5	C11	-0.5(2)
C8	C9	C10	C11	0.1(4)

Table 6: Hydrogen Fractional Atomic Coordinates (×10^4) and Equivalent Isotropic Displacement Parameters (Å^2×10^3) for **DS3-076-2_150K.** U_{eq} is defined as 1/3 of the trace of the orthogonalised U_{ij}.

Atom	x	y	z	U_{eq}
H3A	5821.67	2106.6	5482.49	32
H3B	5598.49	2237.8	4591.23	32
H4	6100.44	5300.9	5345.12	34
H8	6423.58	2410.61	6653.07	49
H11	6569.21	5143.5	6935.44	54
H9	6854.16	2973.05	7970.84	68
H10	6928.4	4345.14	8107.08	69
H2A	5490.48	5209.61	2212.65	86
H2B	6071.62	5946.91	2231.22	86
H2C	5175.58	6049.53	2528.6	86
H1	5474(11)	4437(11)	3303(14)	44(5)

Citations

O.V. Dolomanov and L.J. Bourhis and R.J. Gildea and J.A.K. Howard and H. Puschmann, Olex2: A complete structure solution, refinement and analysis program, *J. Appl. Cryst.*, (2009), **42**, 339-341.

STOE & Cie GmbH, X-Area, software package for collecting single-crystal or multi-domain crystal data on STOE area-detector diffractometers, for image processing, for the correction and scaling of reflection intensities and for outlier rejection, version 1.90, Darmstadt 2020

Sheldrick, G.M., Crystal structure refinement with ShelXL, *Acta Cryst.*, (2015), **C71**, 3-8.

Sheldrick, G.M., ShelXT-Integrated space-group and crystal-structure determination, *Acta Cryst.*, (2015), **A71**, 3-8.

X-Area Integrate 1.78.3.0

X-Area Pilatus3_SV 1.31.170.0 (STOE, 2020)

X-Area Recipe 1.36.0.0

6. ^{1}H and ^{13}C{^{1}H} NMR Chart

^{1}H NMR (500 MHz, DMSO-d$_6$, 298K)

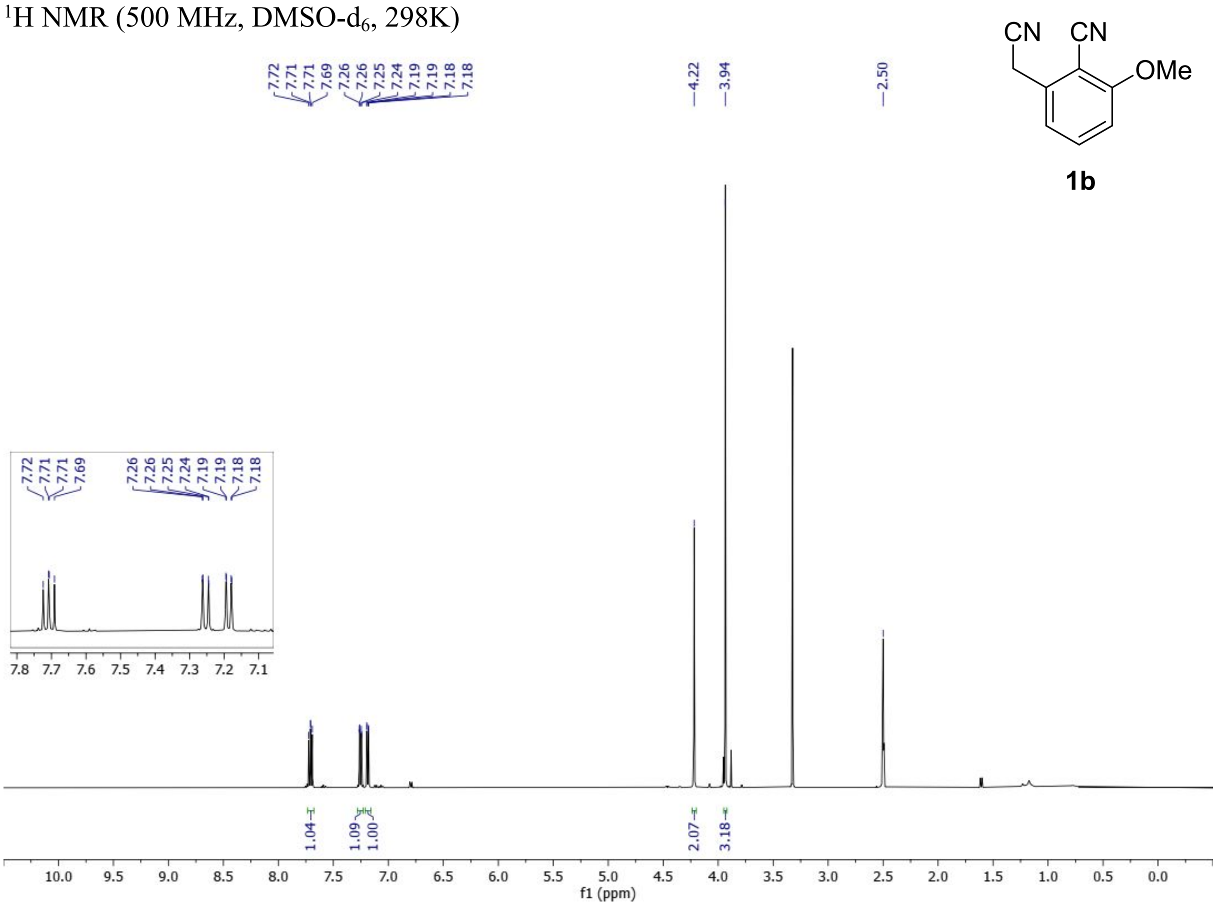

1b

^{13}C{^{1}H} NMR (126 MHz, DMSO-d$_6$, 298K)

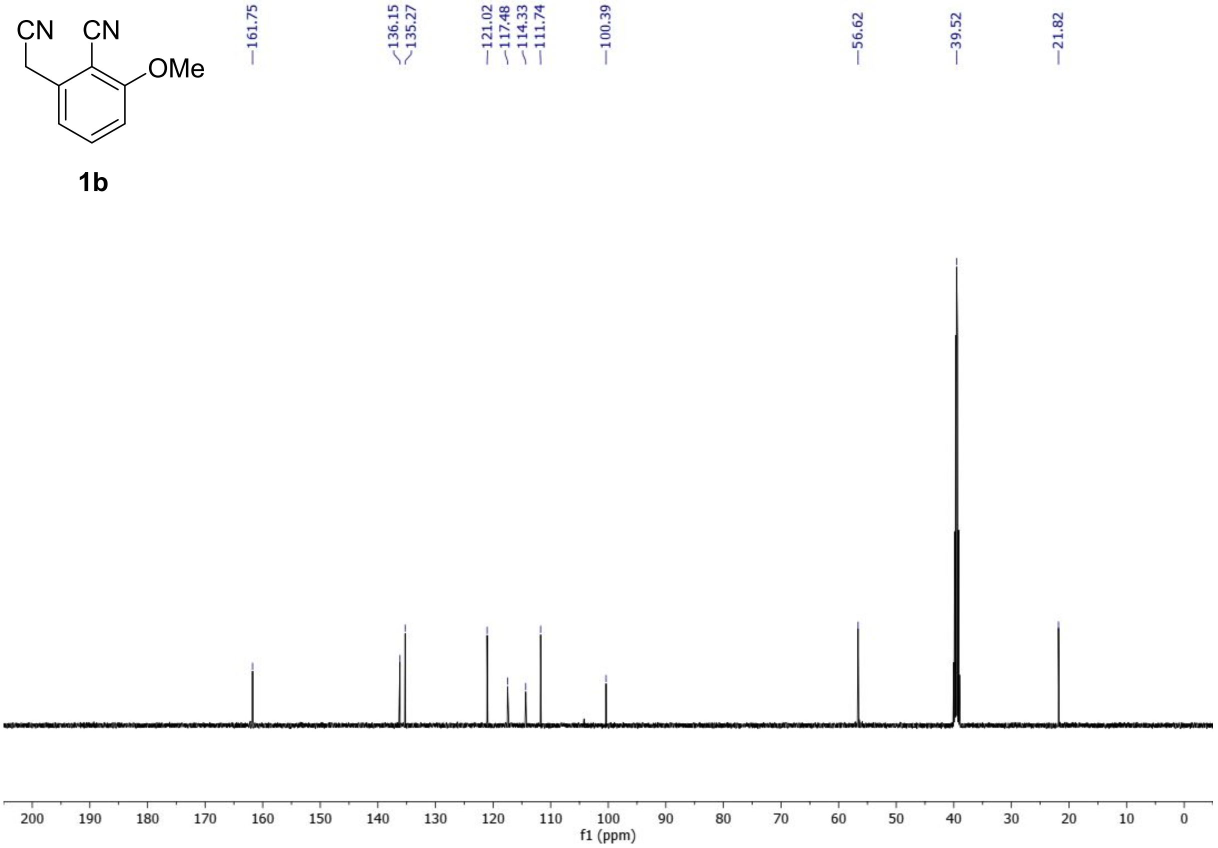

1b

^{1}H NMR (500 MHz, DMSO-d$_6$, 298K)

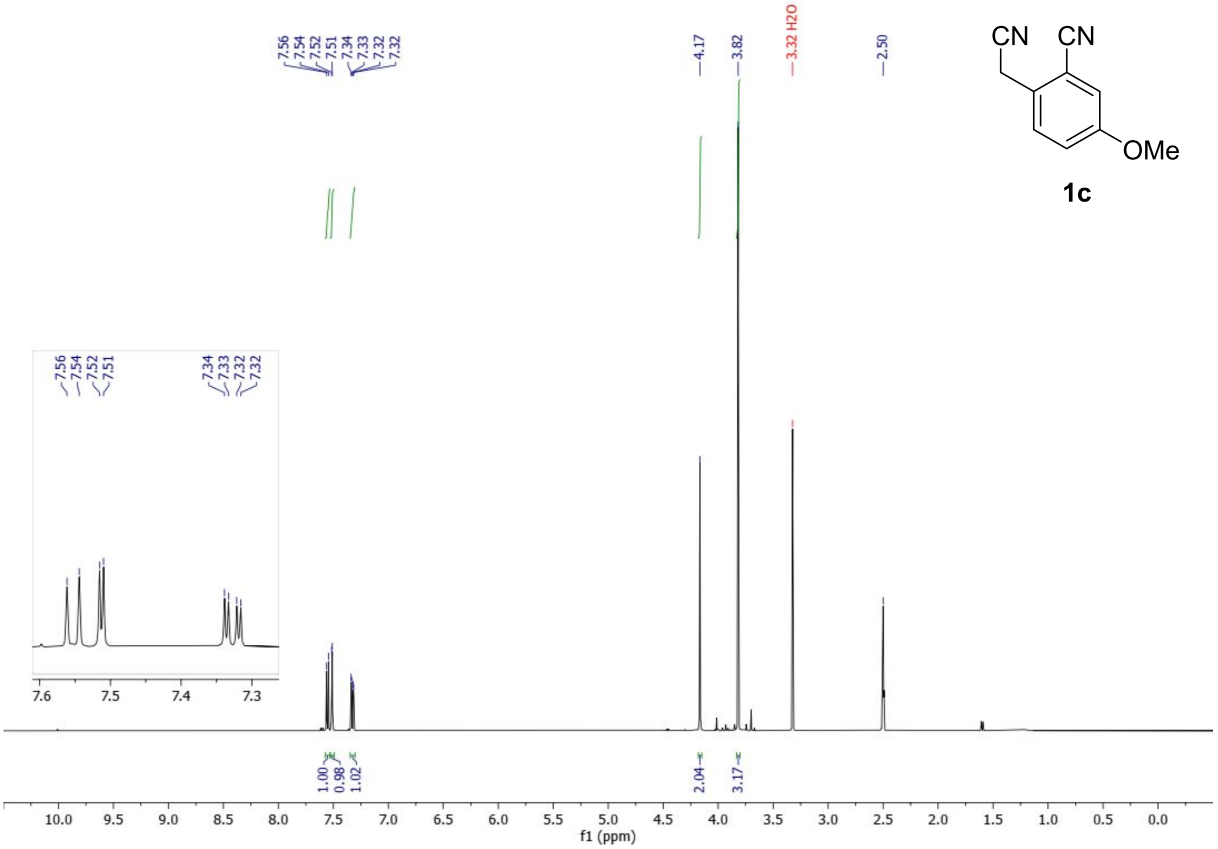

1c

$^{13}C\{^{1}H\}$ NMR (126 MHz, DMSO-d$_6$, 298K)

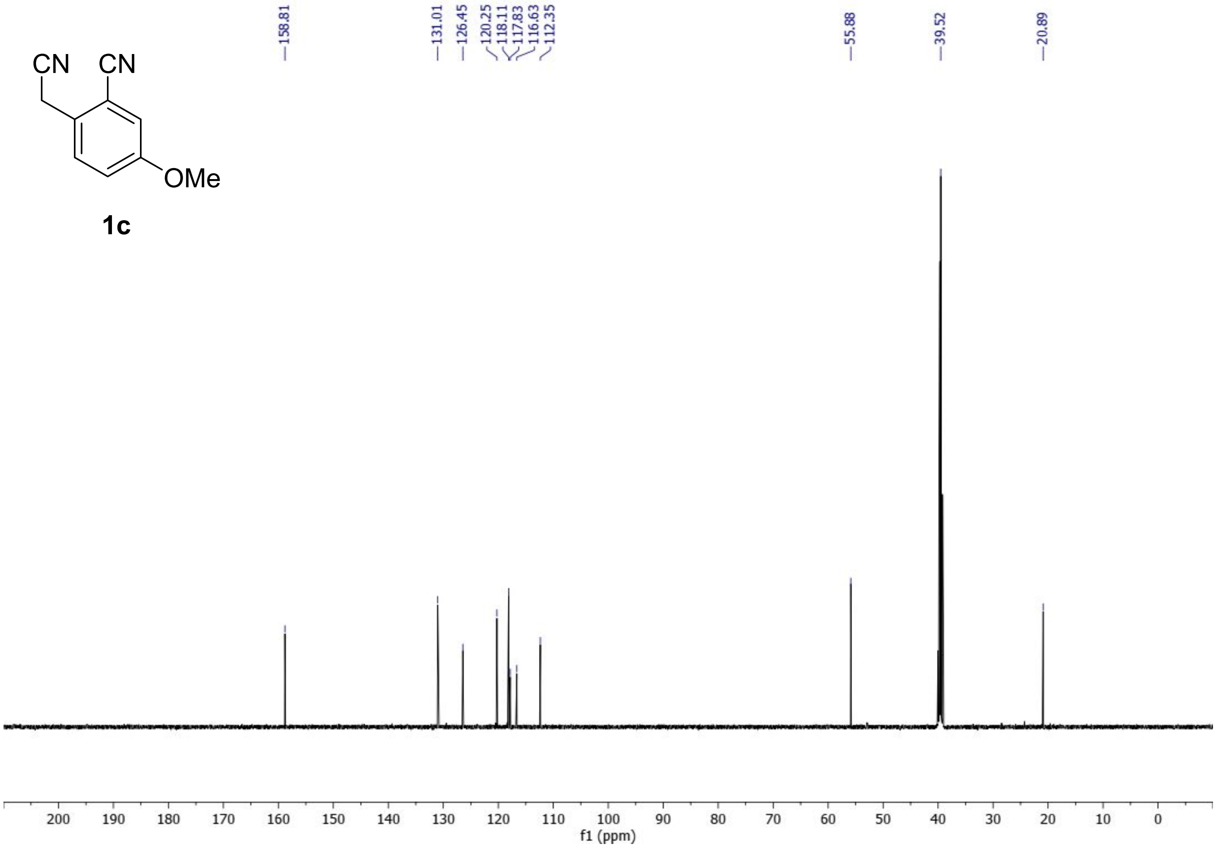

^{1}H NMR (500 MHz, DMSO-d_6, 298K)

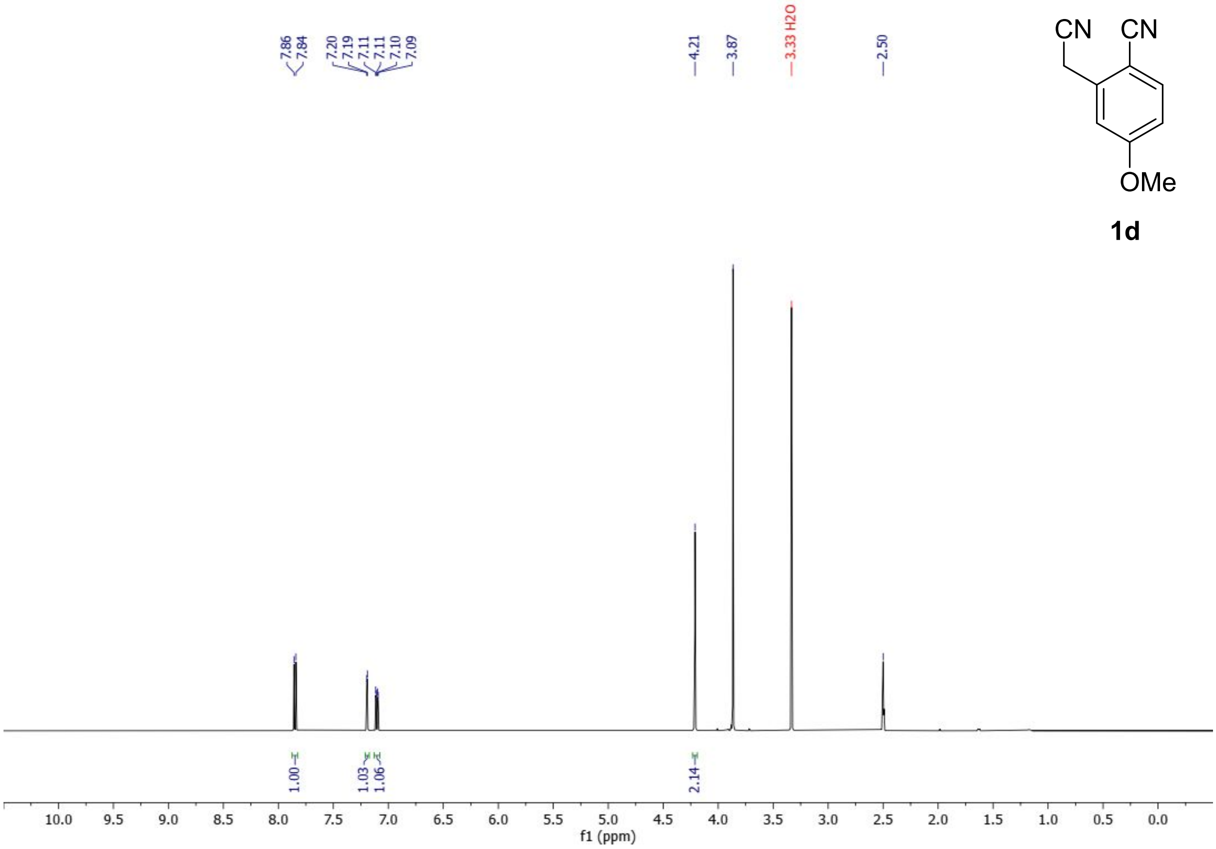

1d

$^{13}C\{^{1}H\}$ NMR (126 MHz, DMSO-d_6, 298K)

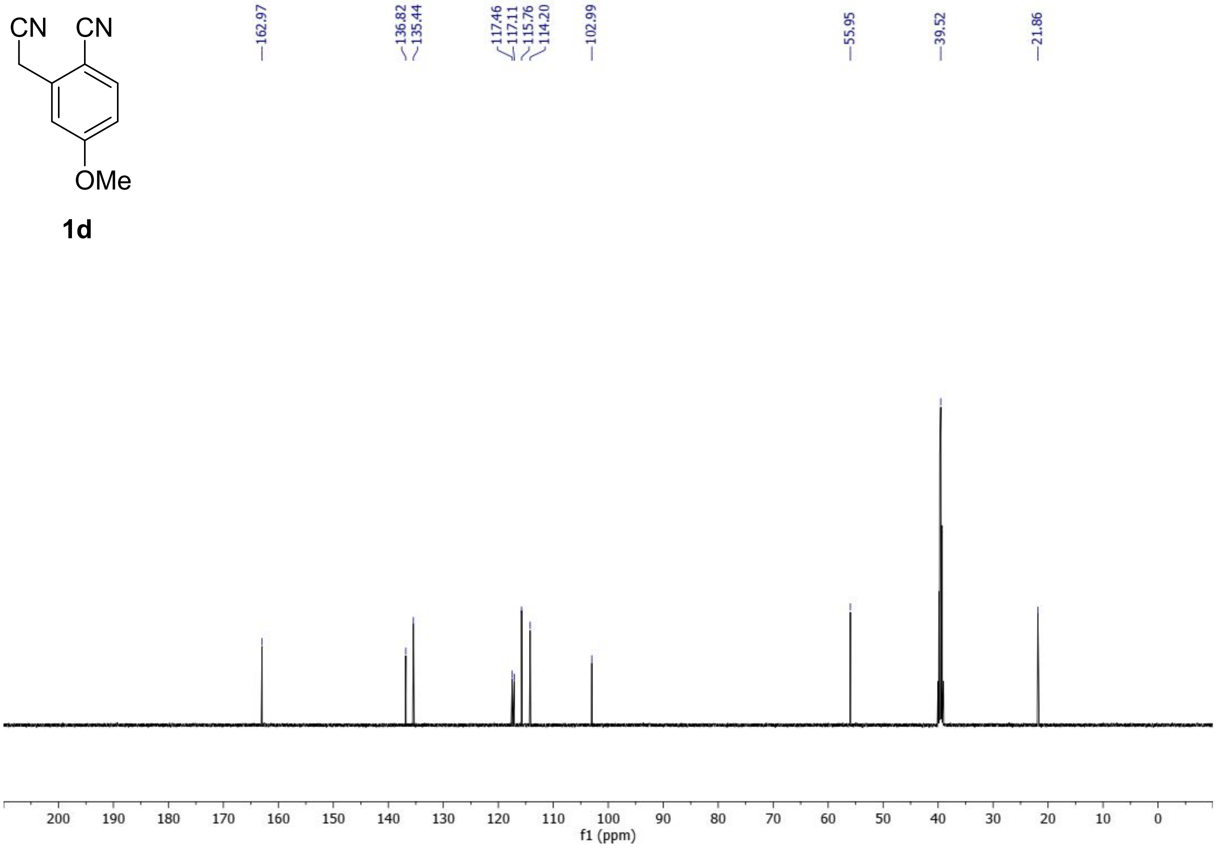

1d

^{1}H NMR (500 MHz, DMSO-d_6, 298K)

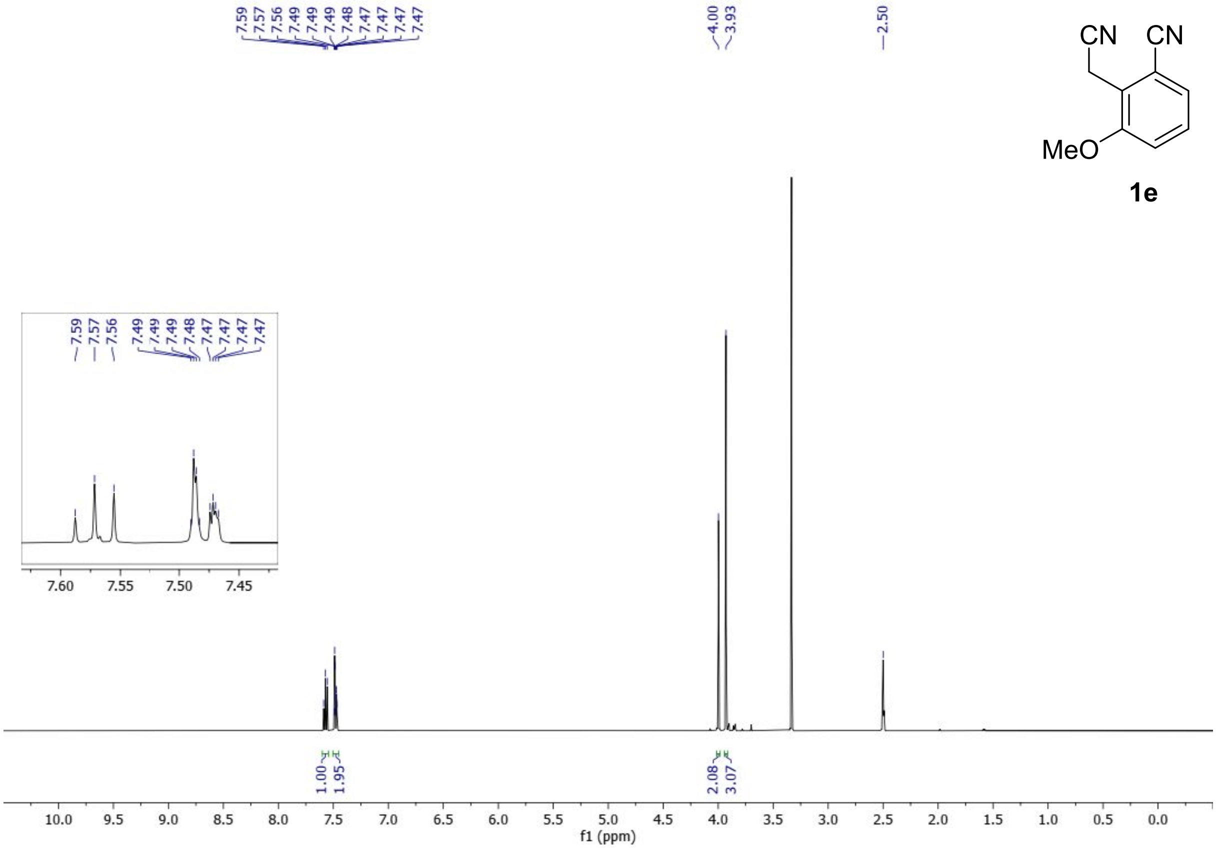

$^{13}C\{^{1}H\}$ NMR (126 MHz, DMSO-d_6, 298K)

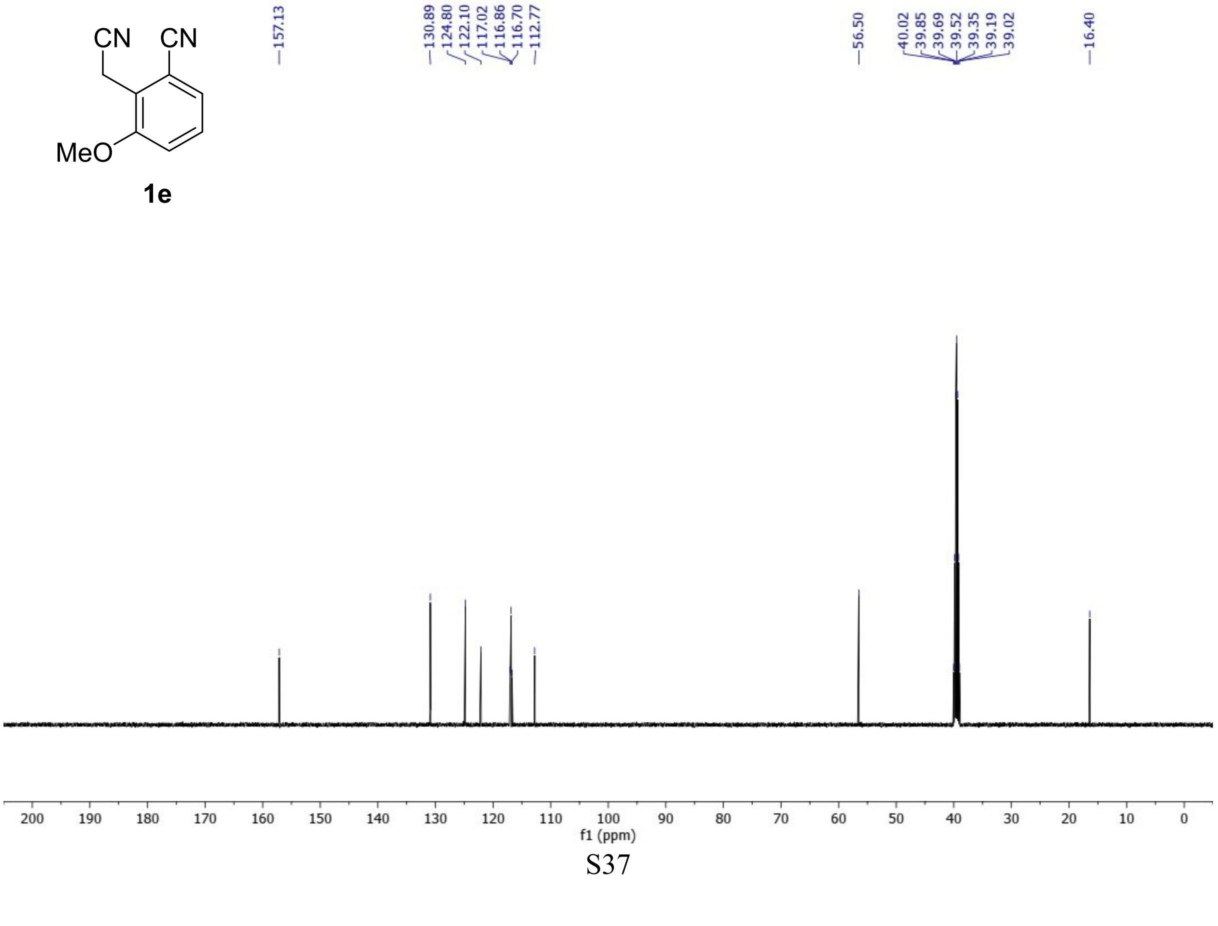

^{1}H NMR (500 MHz, DMSO-d_6, 298K)

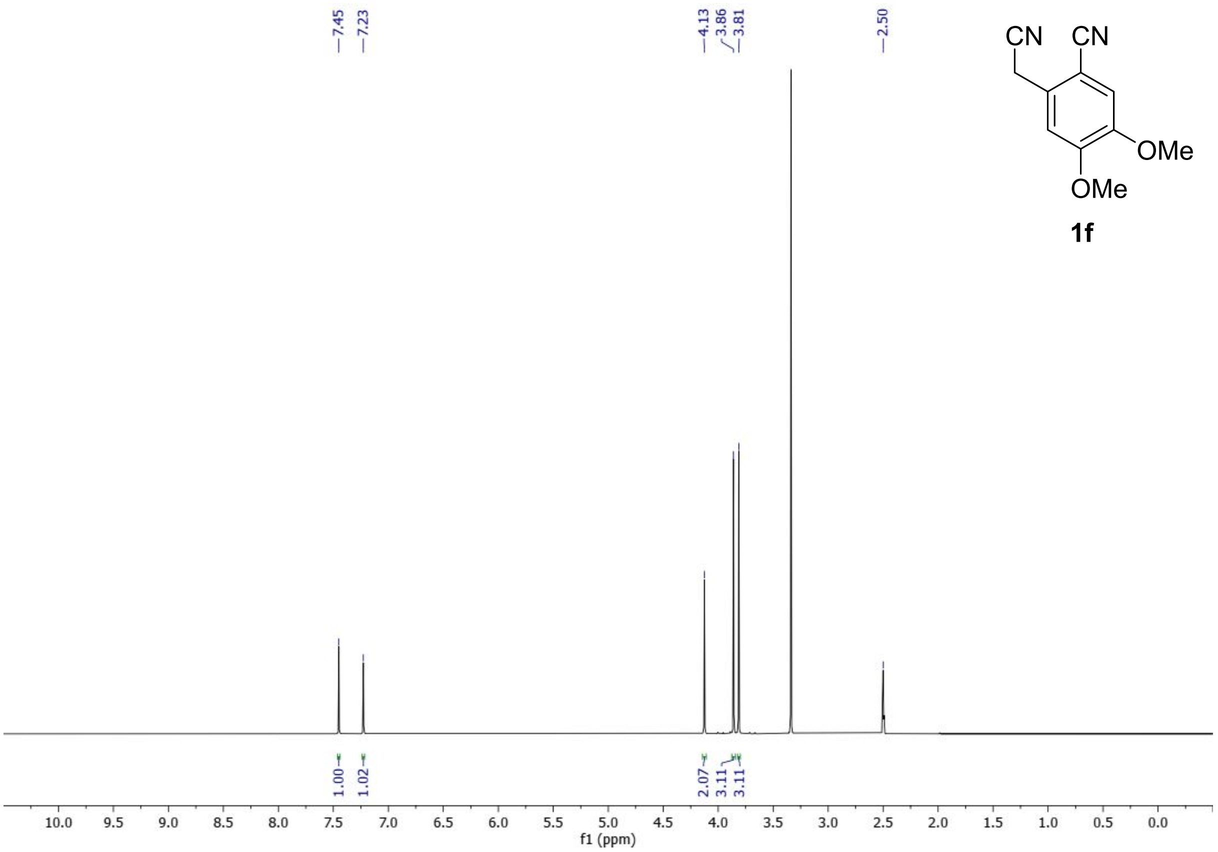

$^{13}C\{^{1}H\}$ NMR (126 MHz, DMSO-d_6, 298K)

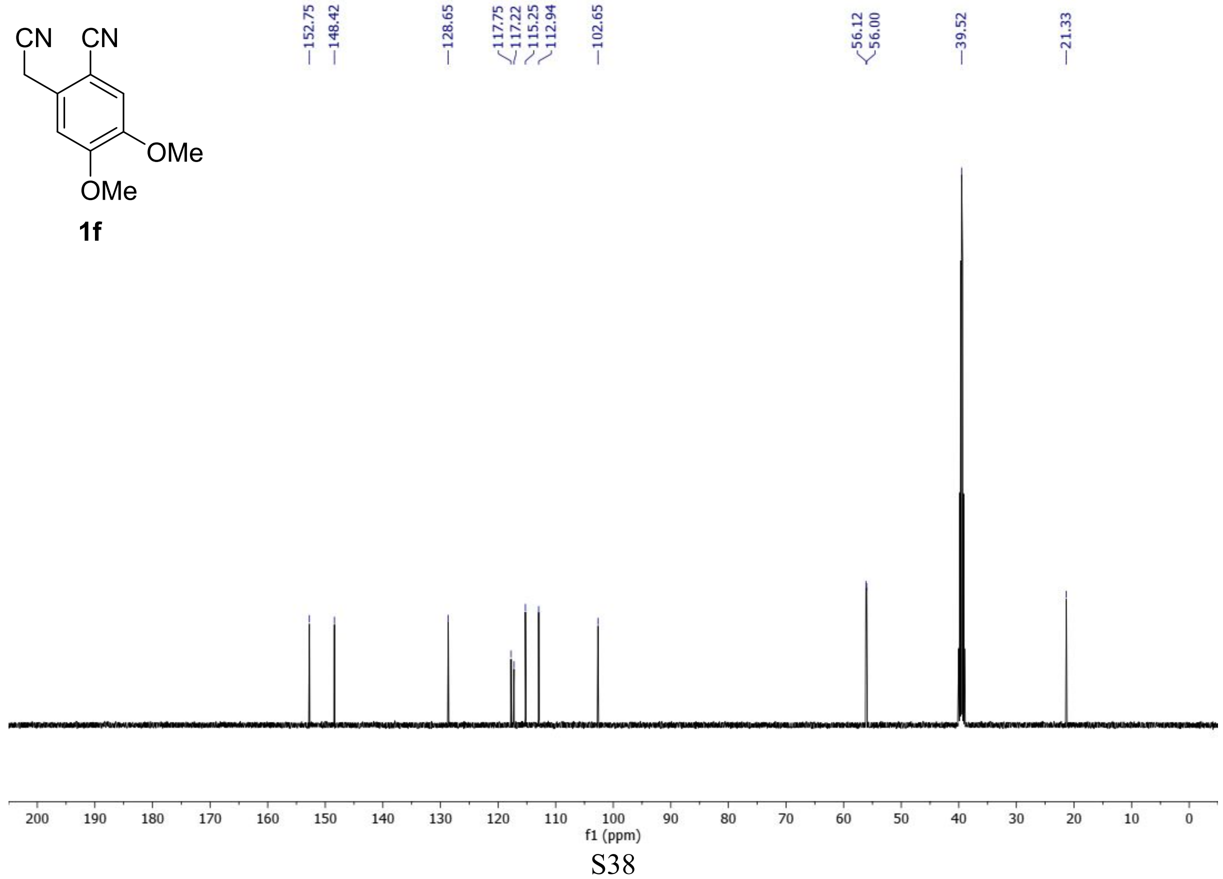

^{1}H NMR (500 MHz, DMSO-d_6, 298K)

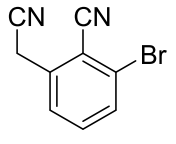

1g

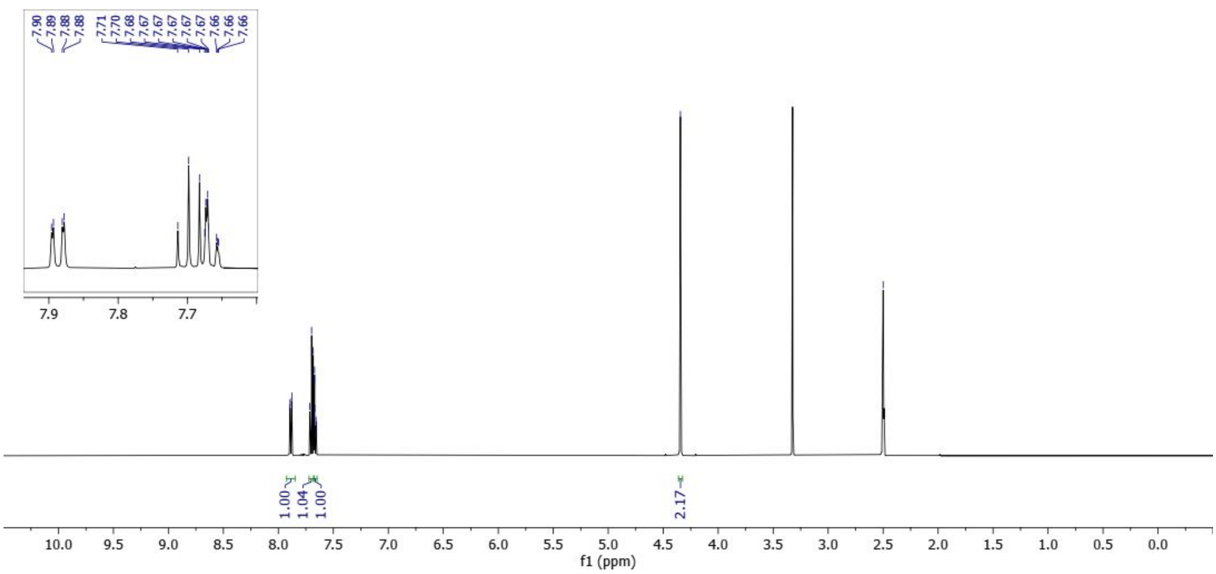

$^{13}C\{^{1}H\}$ NMR (126 MHz, DMSO-d_6, 298K)

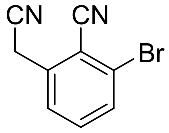

1g

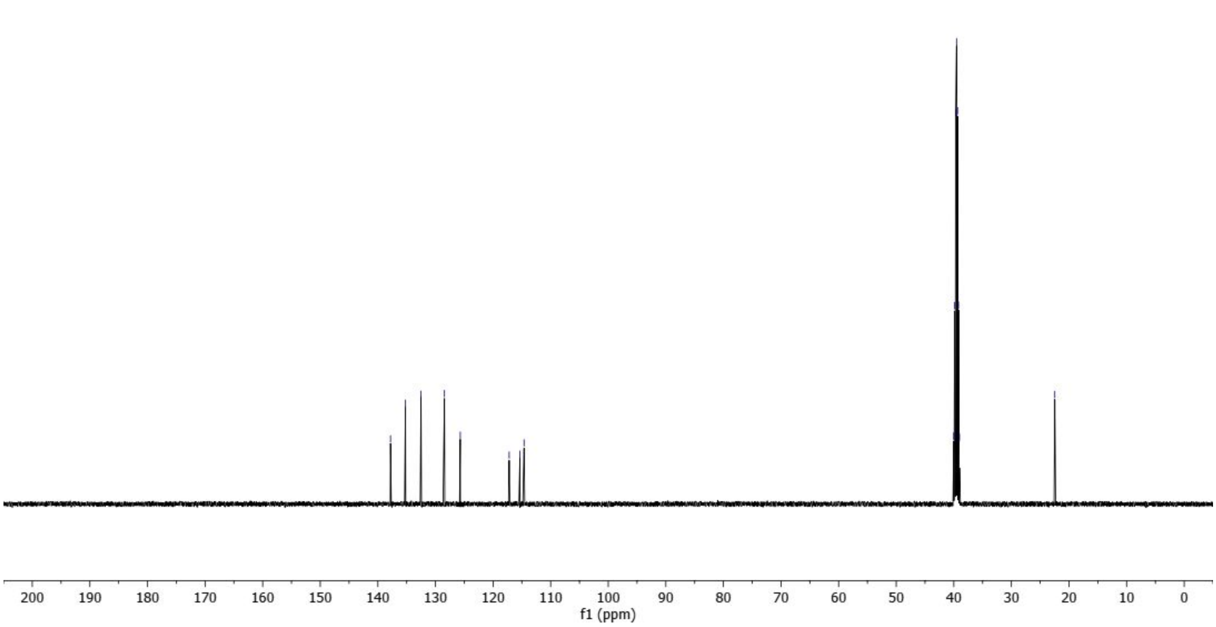

^{1}H NMR (500 MHz, DMSO-d_6, 298K)

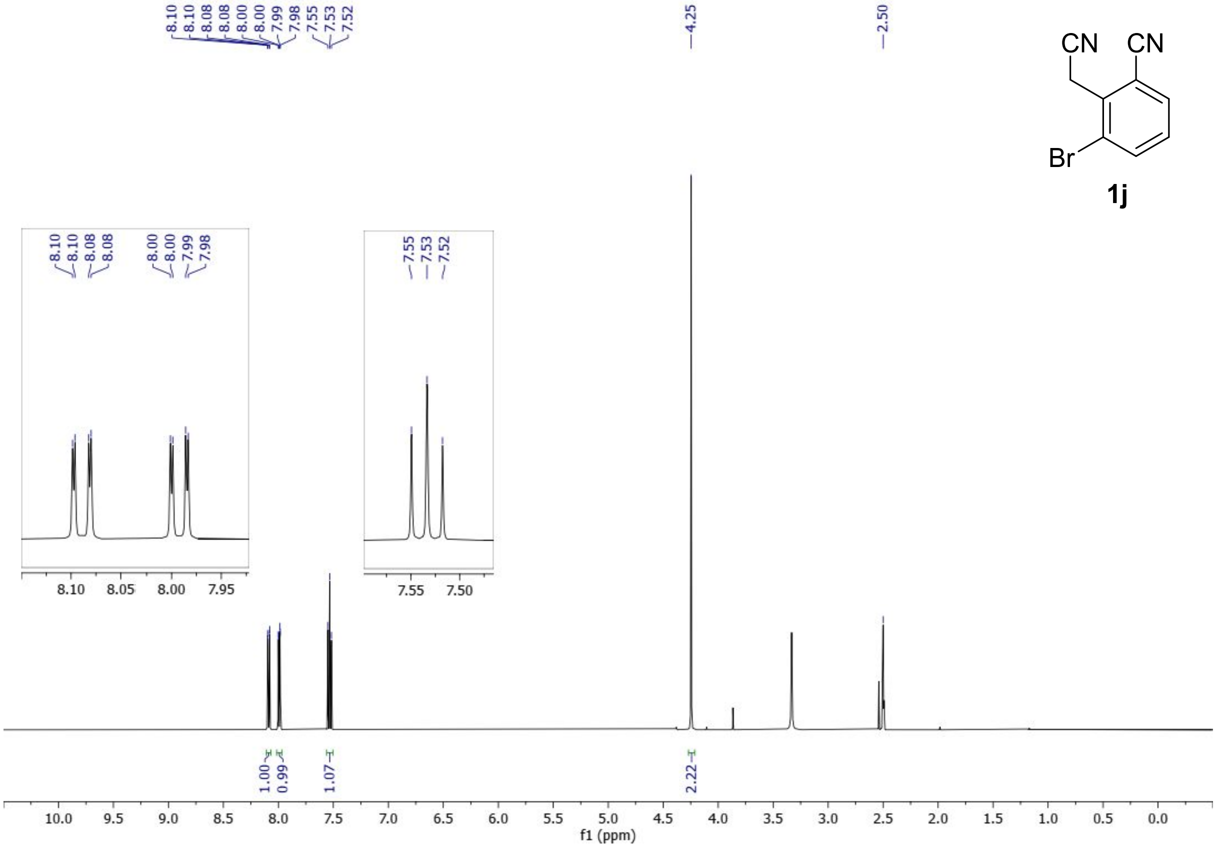

$^{13}C\{^{1}H\}$ NMR (126 MHz, DMSO-d_6, 298K)

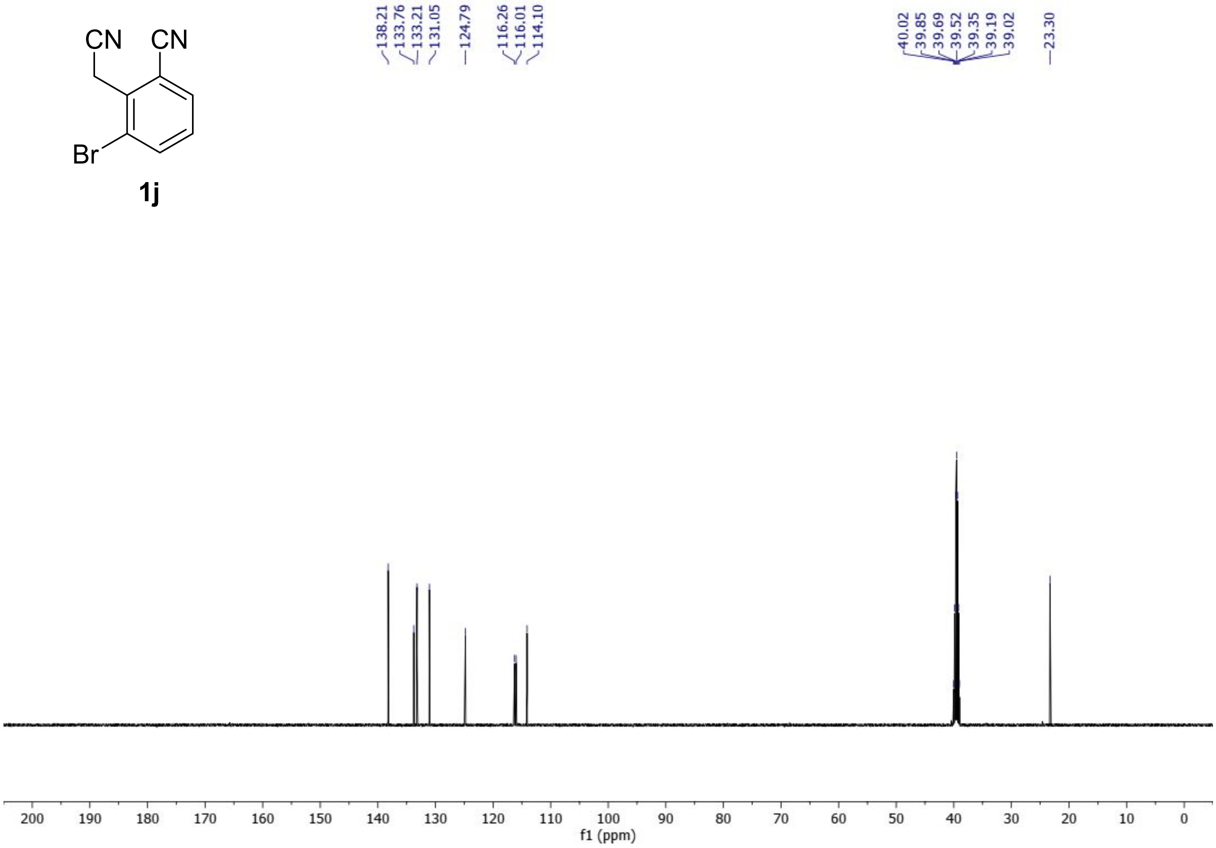

^{1}H NMR (500 MHz, DMSO-d_6, 298K)

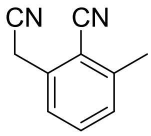

1k

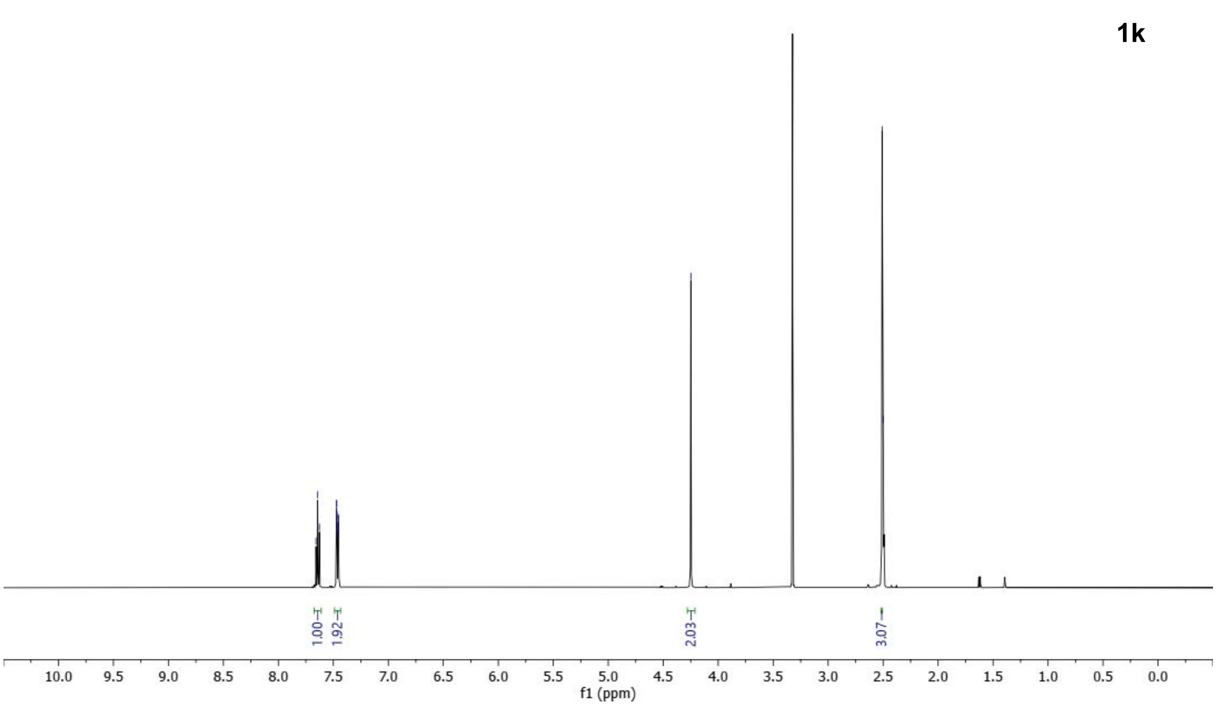

$^{13}C\{^{1}H\}$ NMR (126 MHz, DMSO-d_6, 298K)

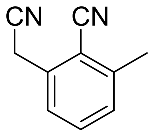

1k

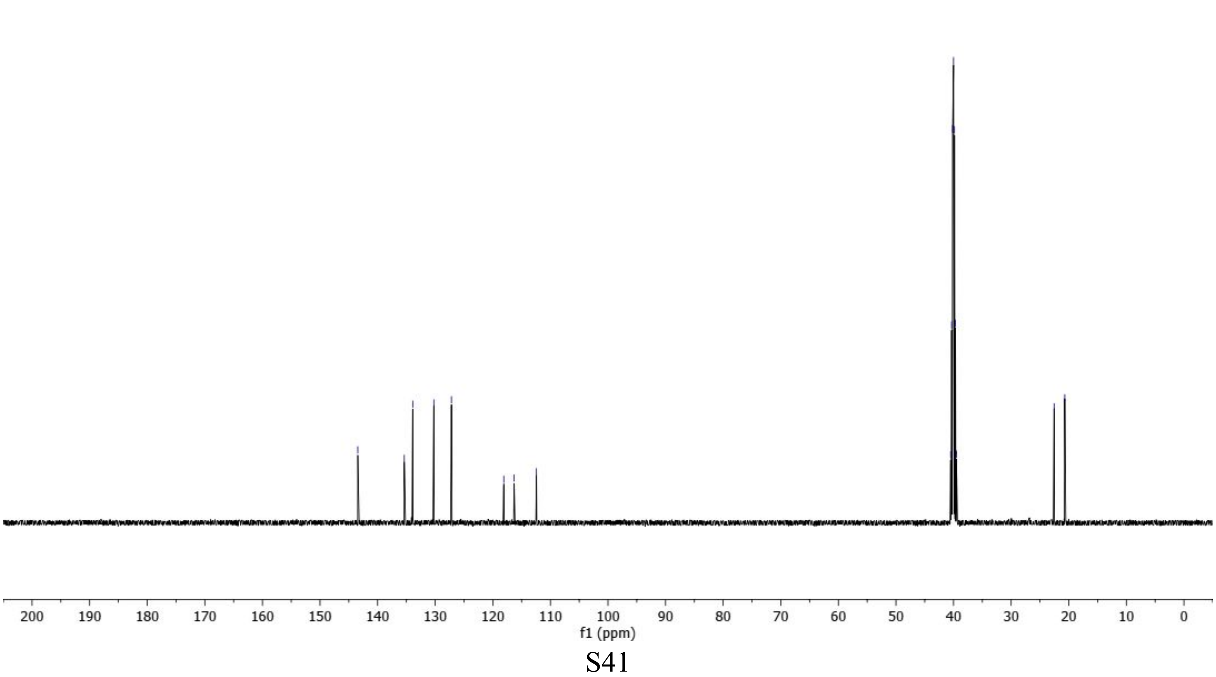

^{1}H NMR (500 MHz, DMSO-d$_6$, 298K)

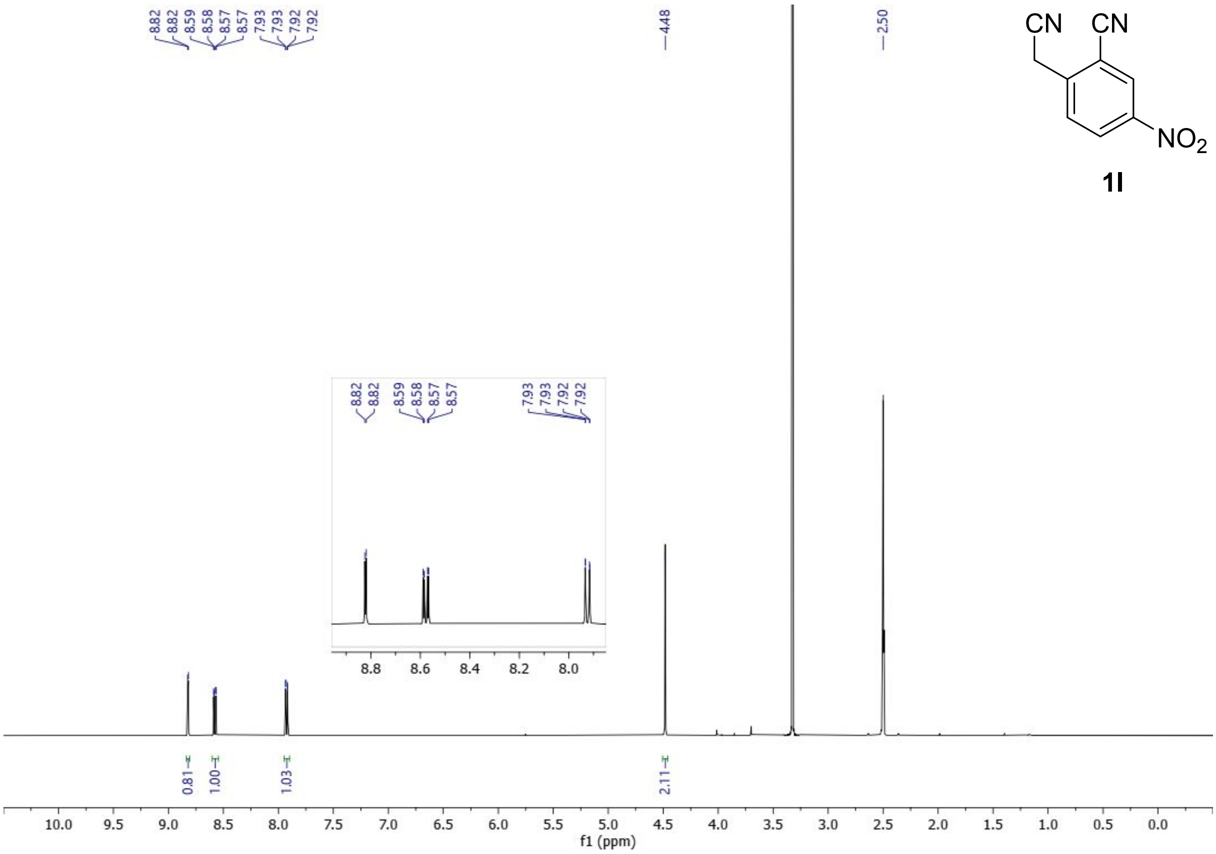

$^{13}C\{^{1}H\}$ NMR (126 MHz, DMSO-d$_6$, 298K)

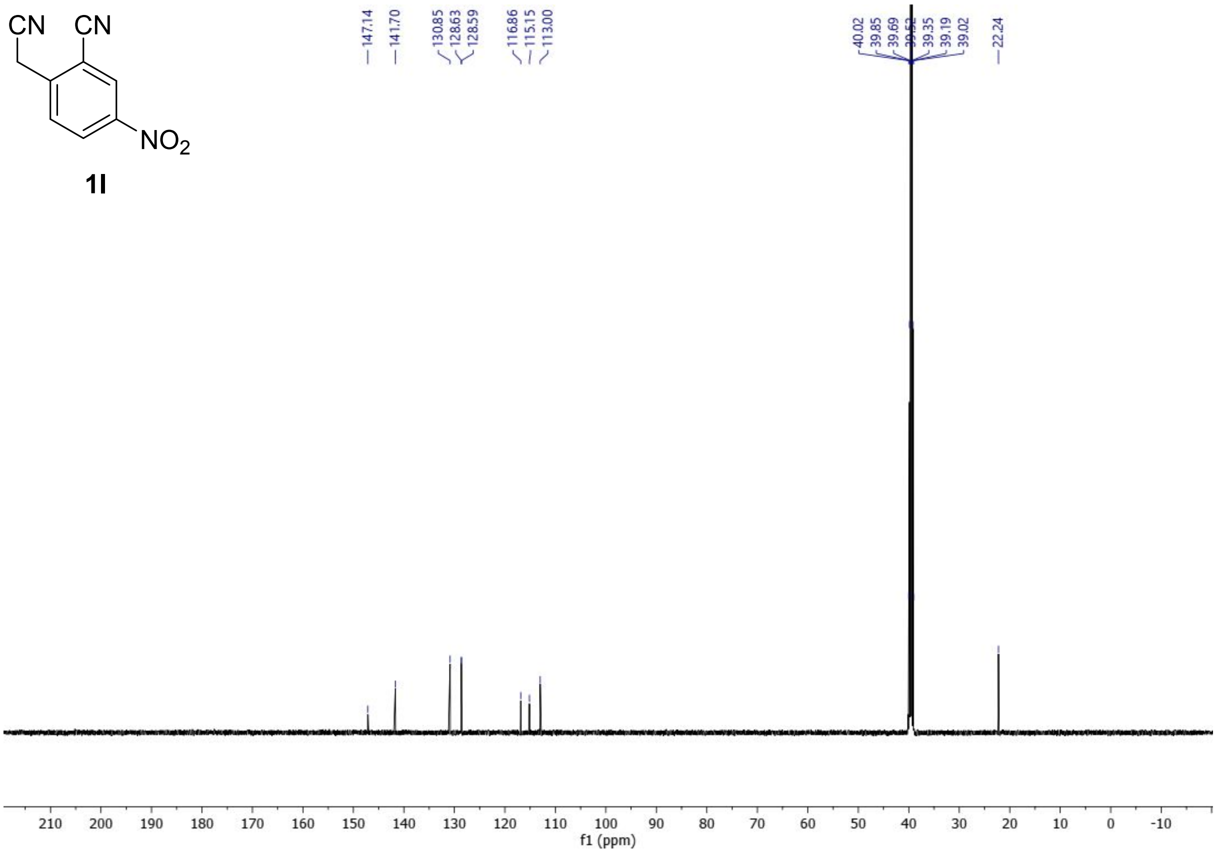

^{1}H NMR (500 MHz, DMSO-d$_6$, 298K)

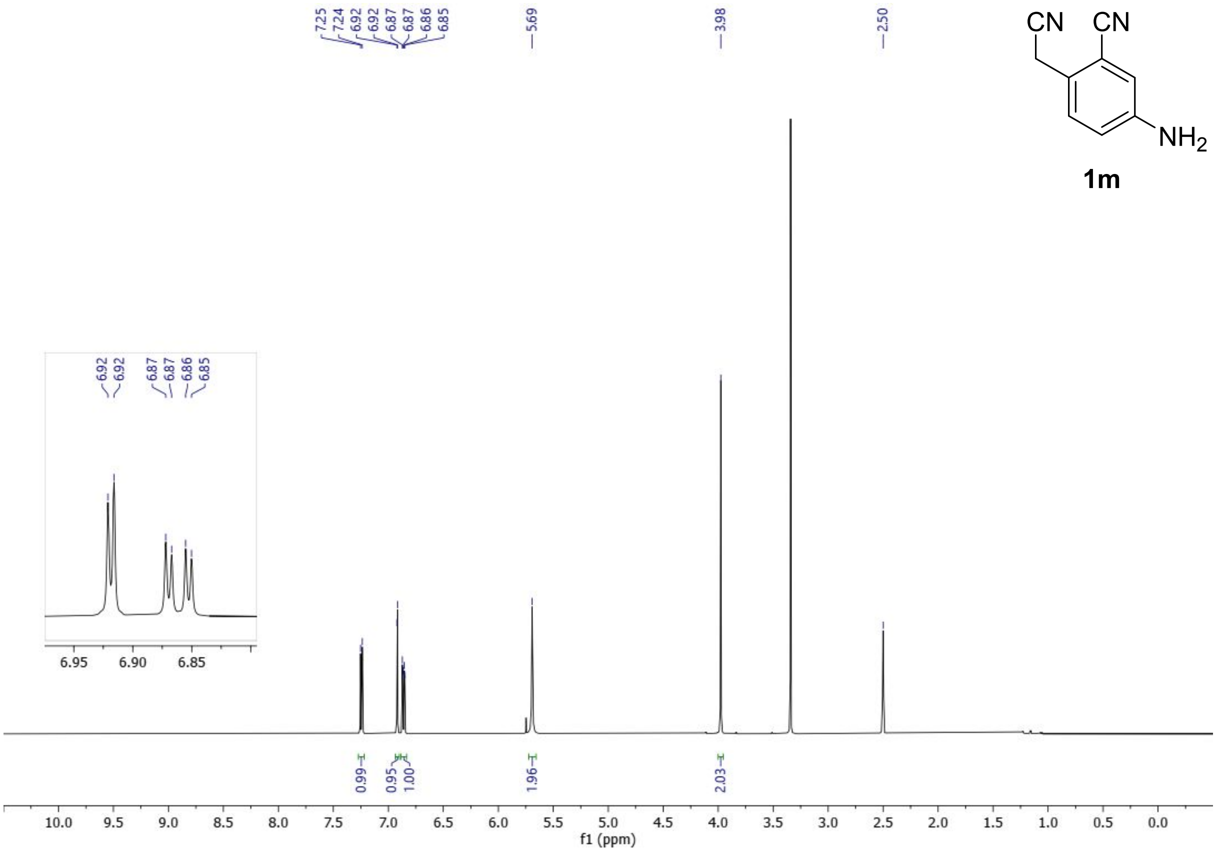

^{13}C{^{1}H} NMR (126 MHz, DMSO-d$_6$, 298K)

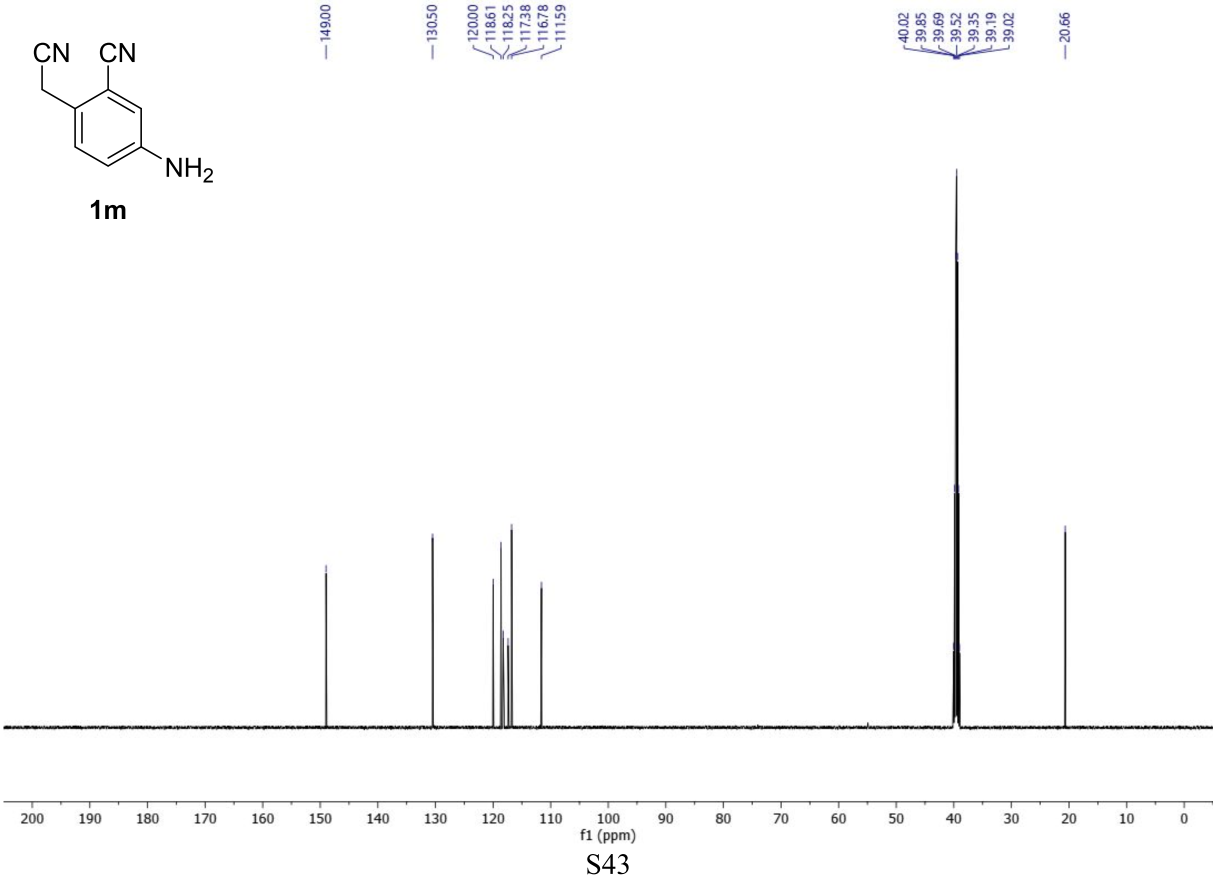

^{1}H NMR (500 MHz, DMSO-d_6, 298K)

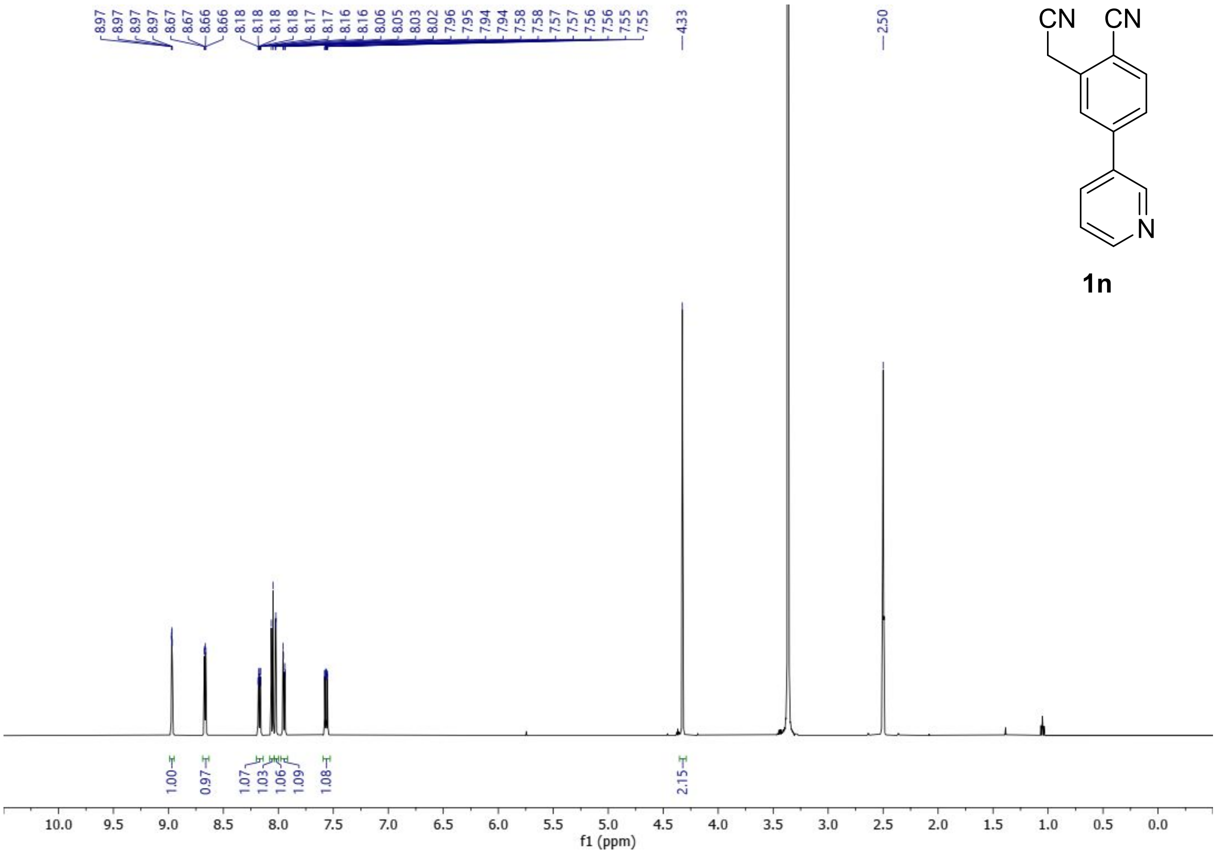

$^{13}C\{^{1}H\}$ NMR (126 MHz, DMSO-d_6, 298K)

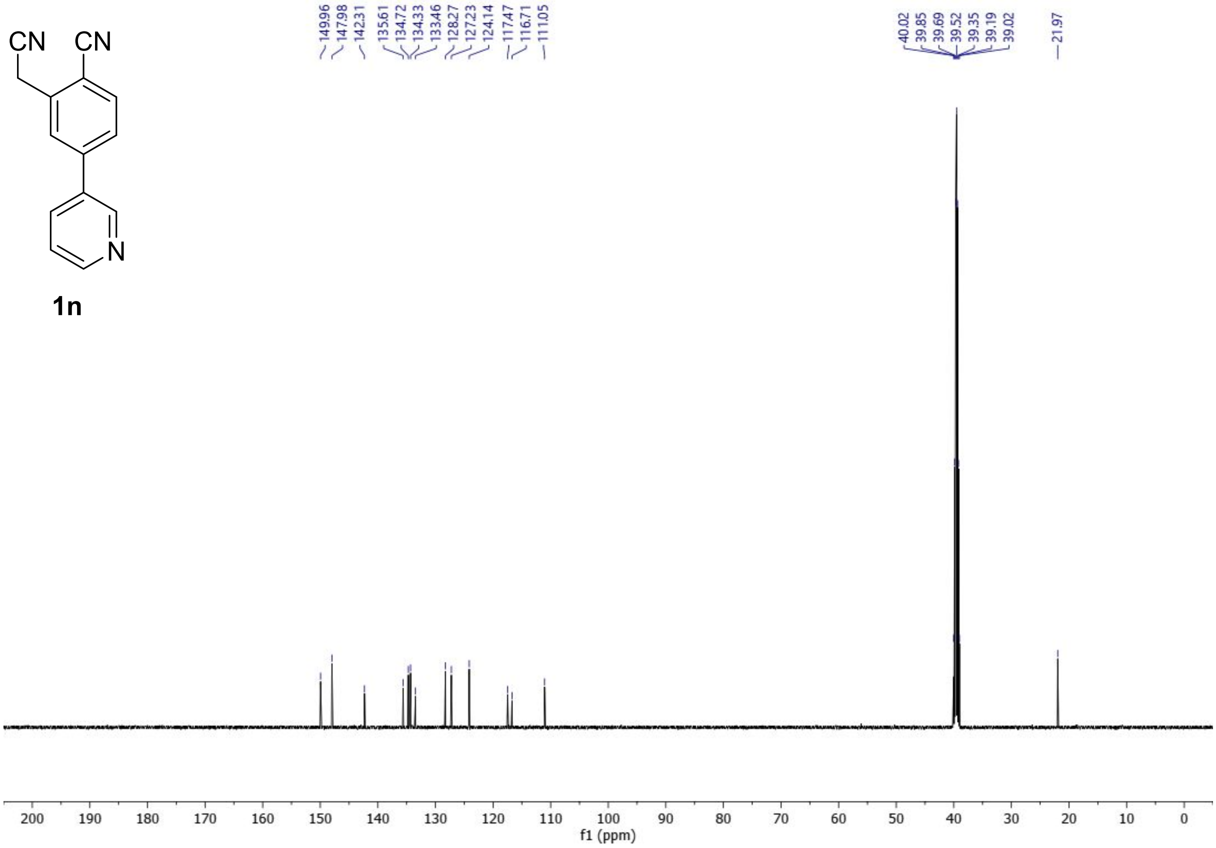

^{1}H NMR (500 MHz, DMSO-d_6, 298K)

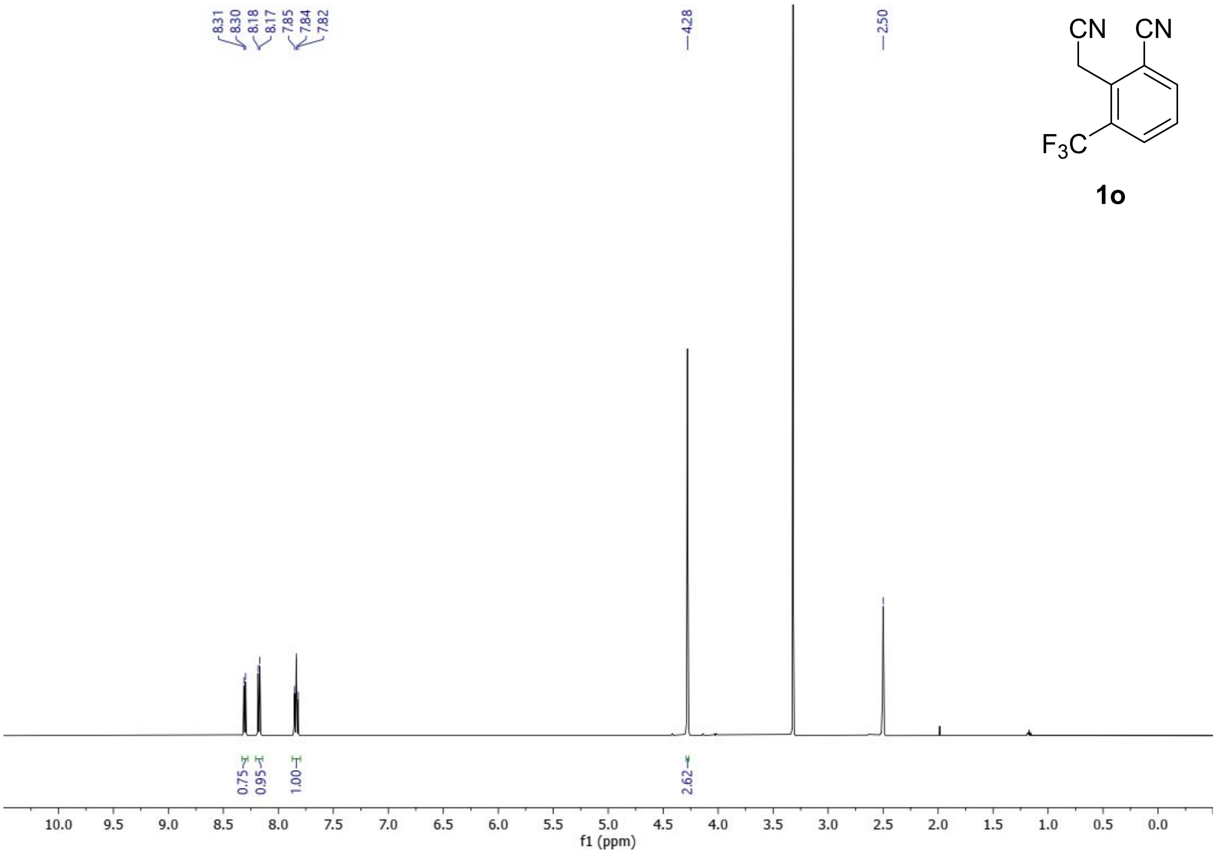

$^{13}C\{^{1}H\}$ NMR (126 MHz, DMSO-d_6, 298K)

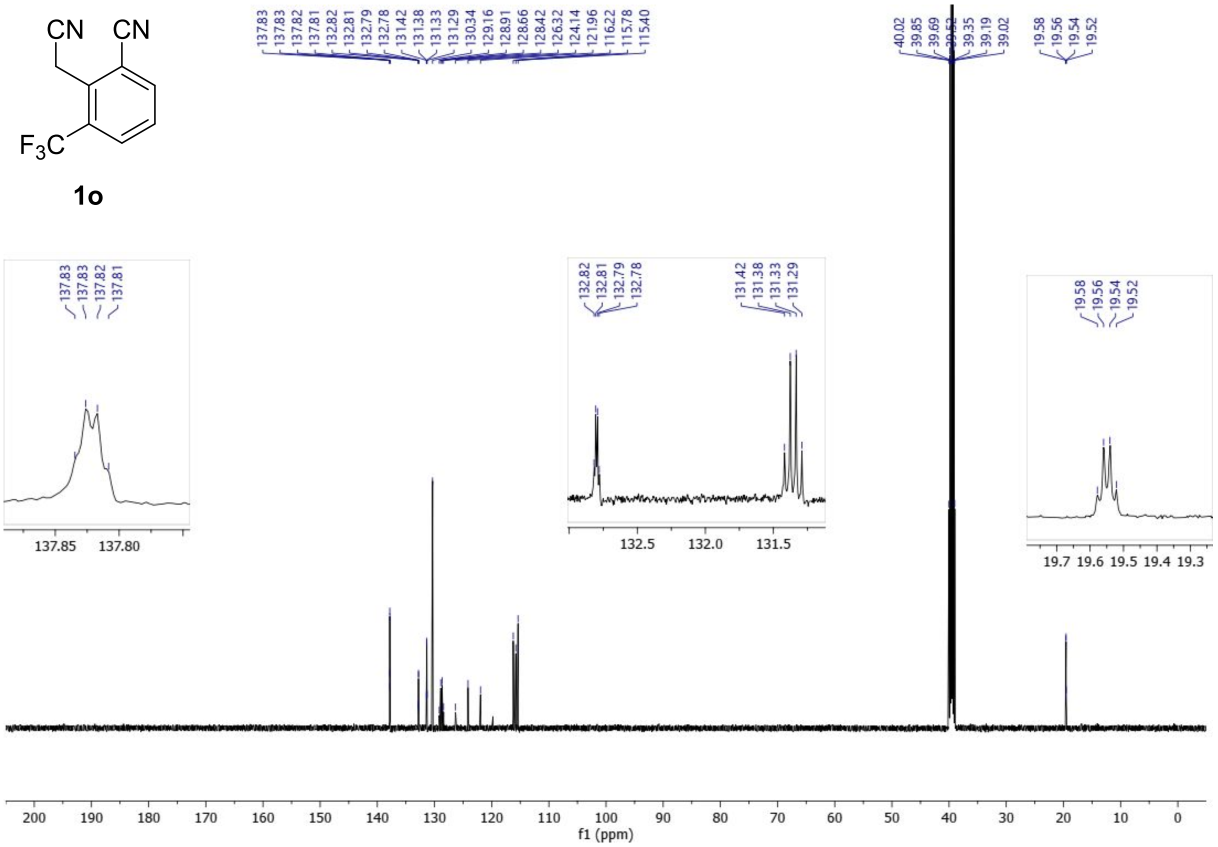

^{1}H NMR (500 MHz, DMSO-d$_6$, 298K)

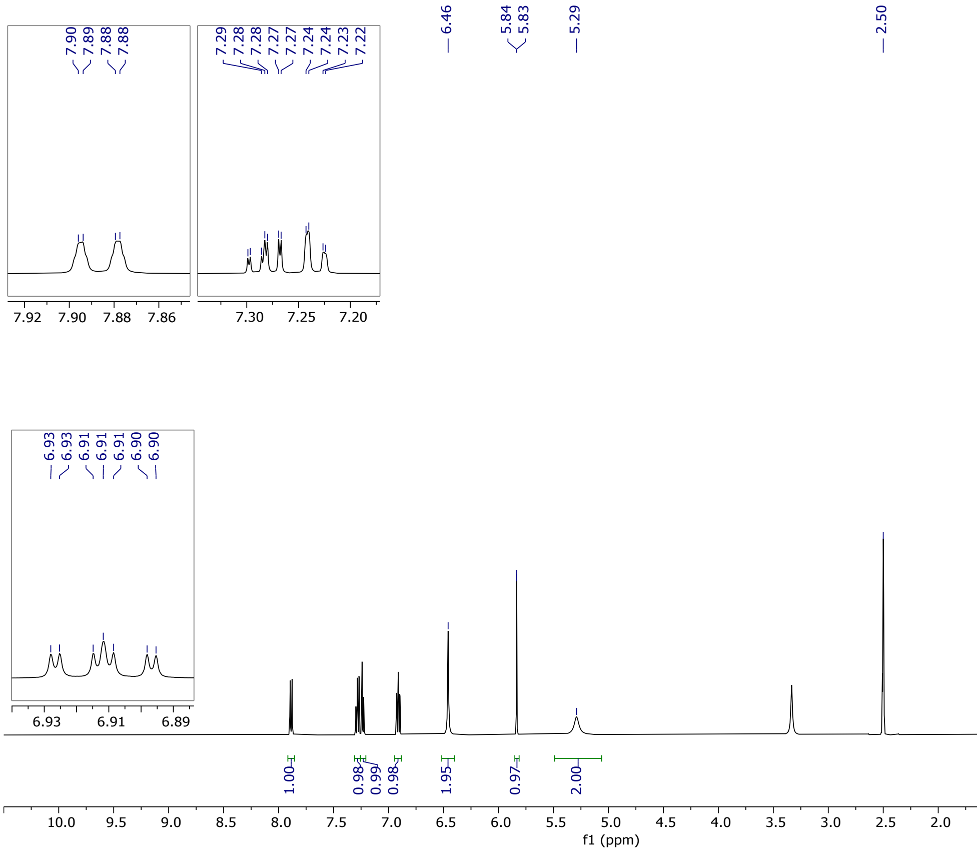

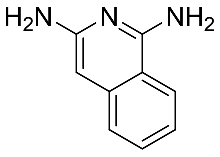

2a

$^{13}C\{^{1}H\}$ NMR (126 MHz, DMSO-d$_6$, 298K)

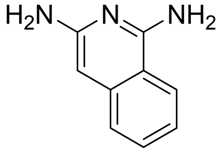

2a

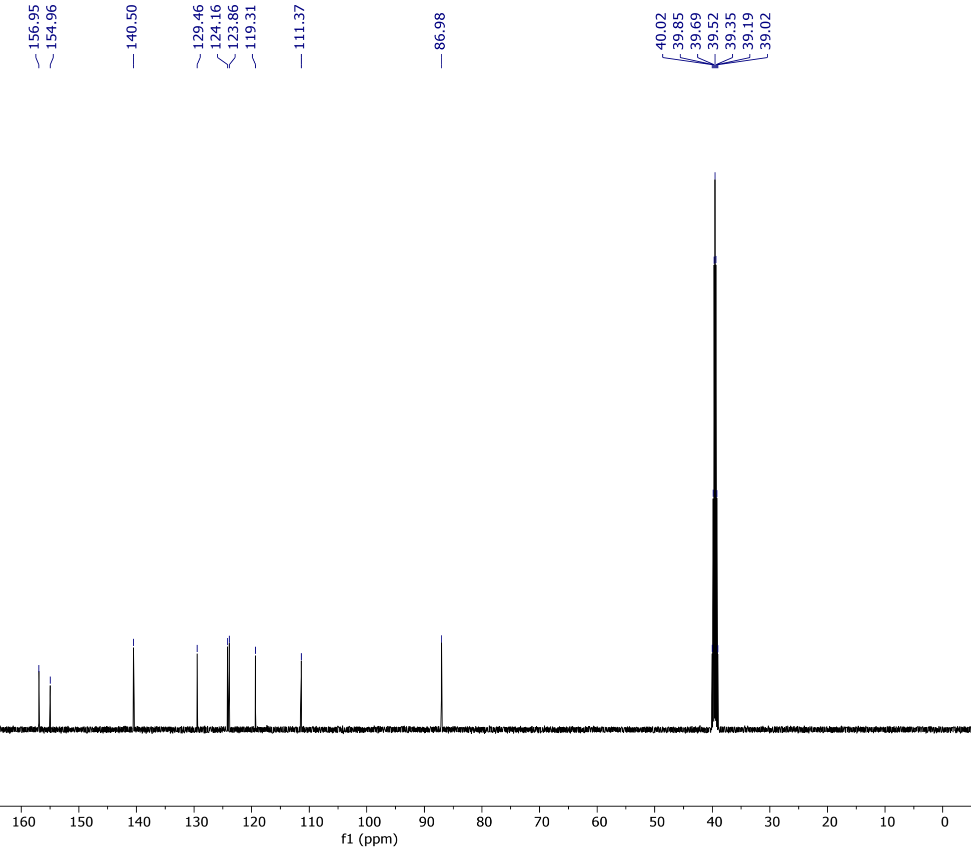

^{1}H NMR (500 MHz, DMSO-d$_6$, 298K)

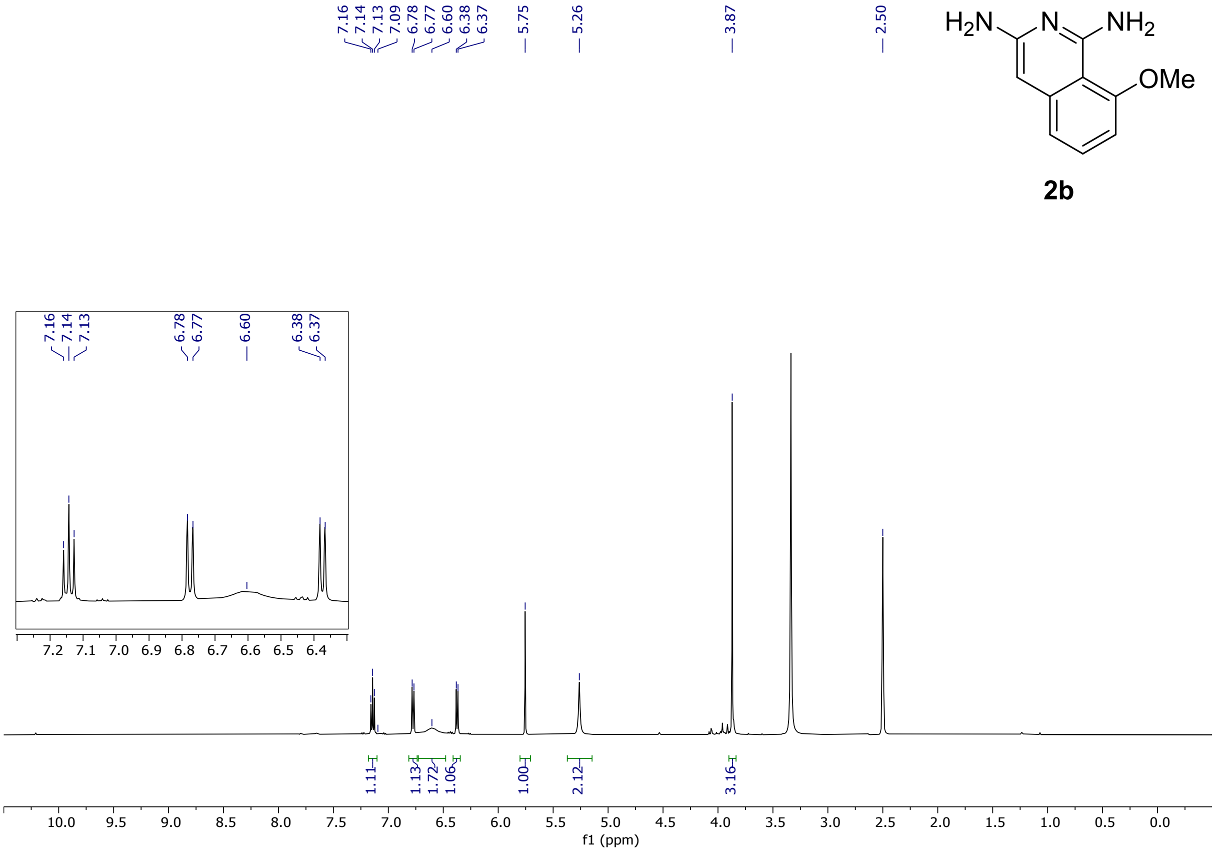

2b

^{13}C{^{1}H} NMR (126 MHz, DMSO-d$_6$, 298K)

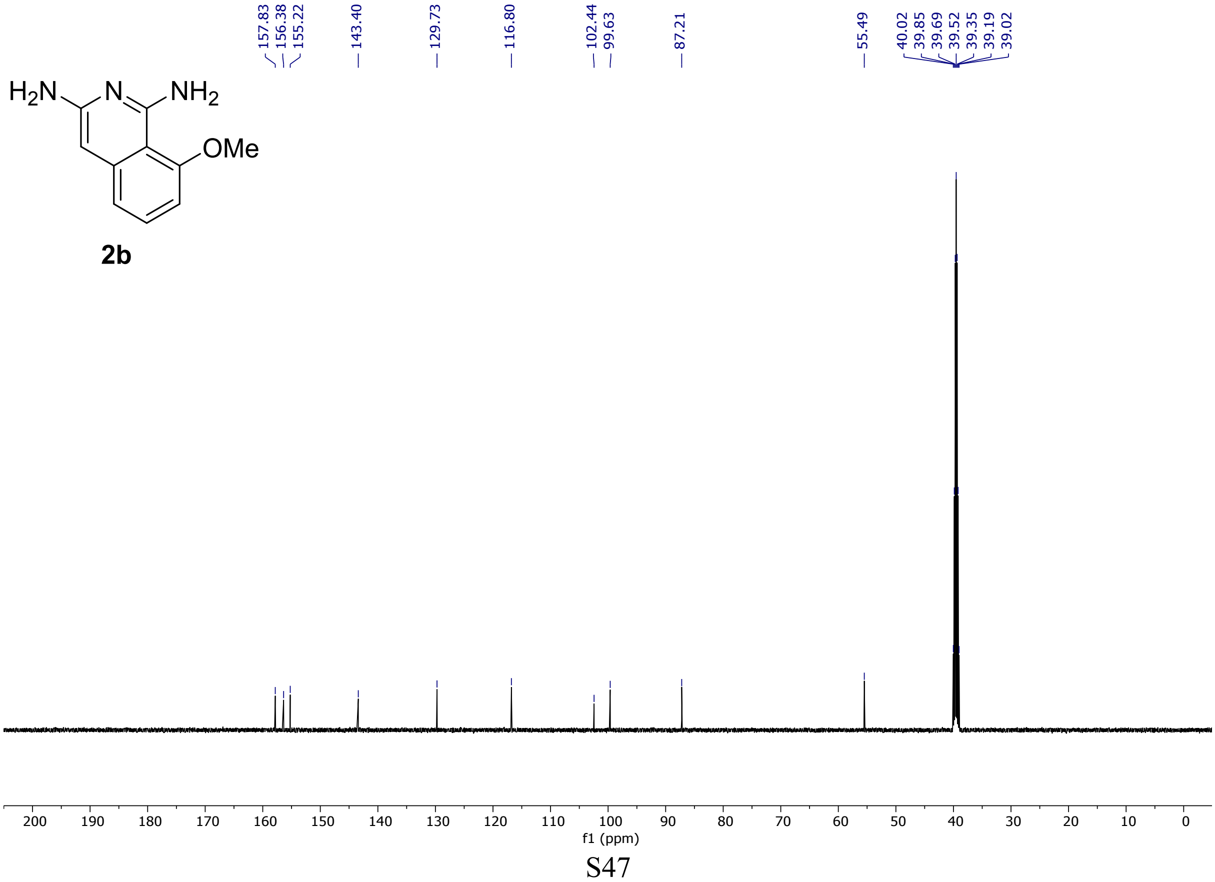

2b

^{1}H NMR (500 MHz, DMSO-d_6, 298K)

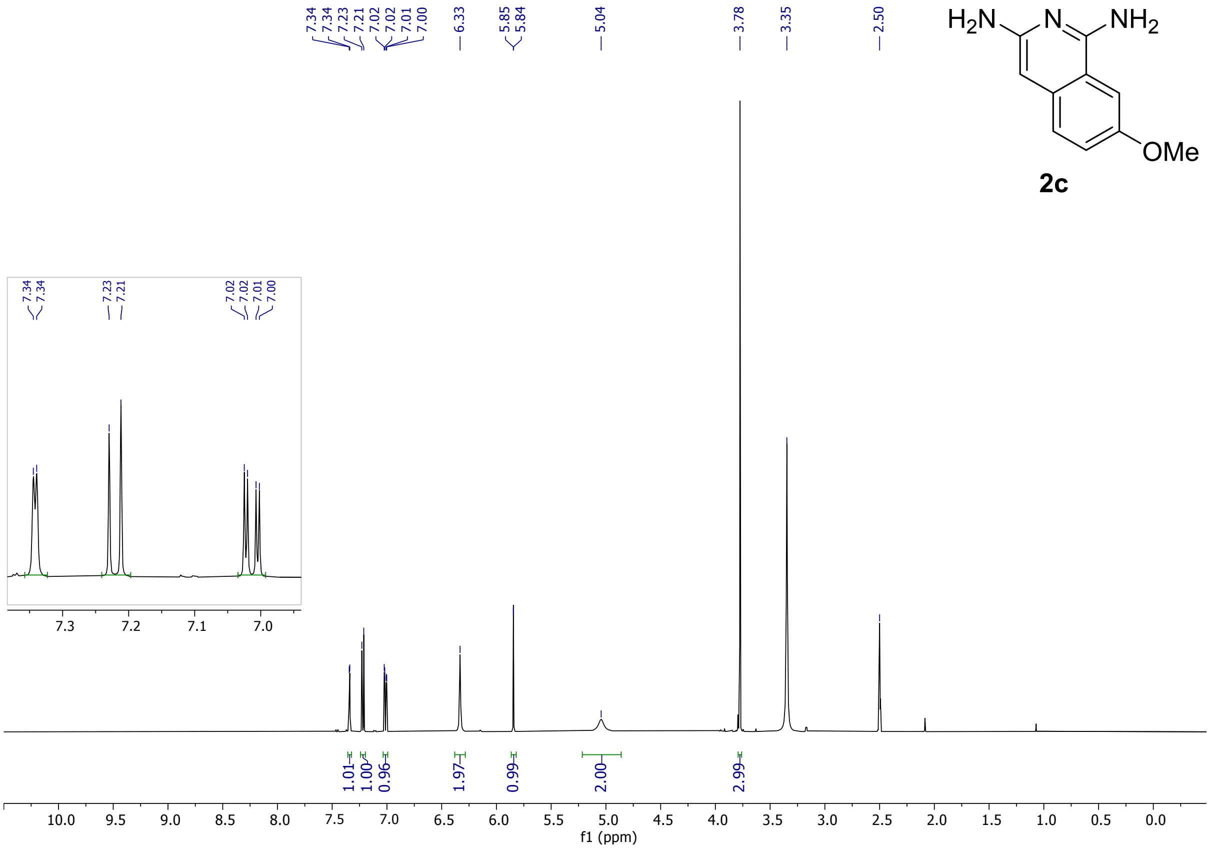

^{13}C{^{1}H} NMR (126 MHz, DMSO-d_6, 298K)

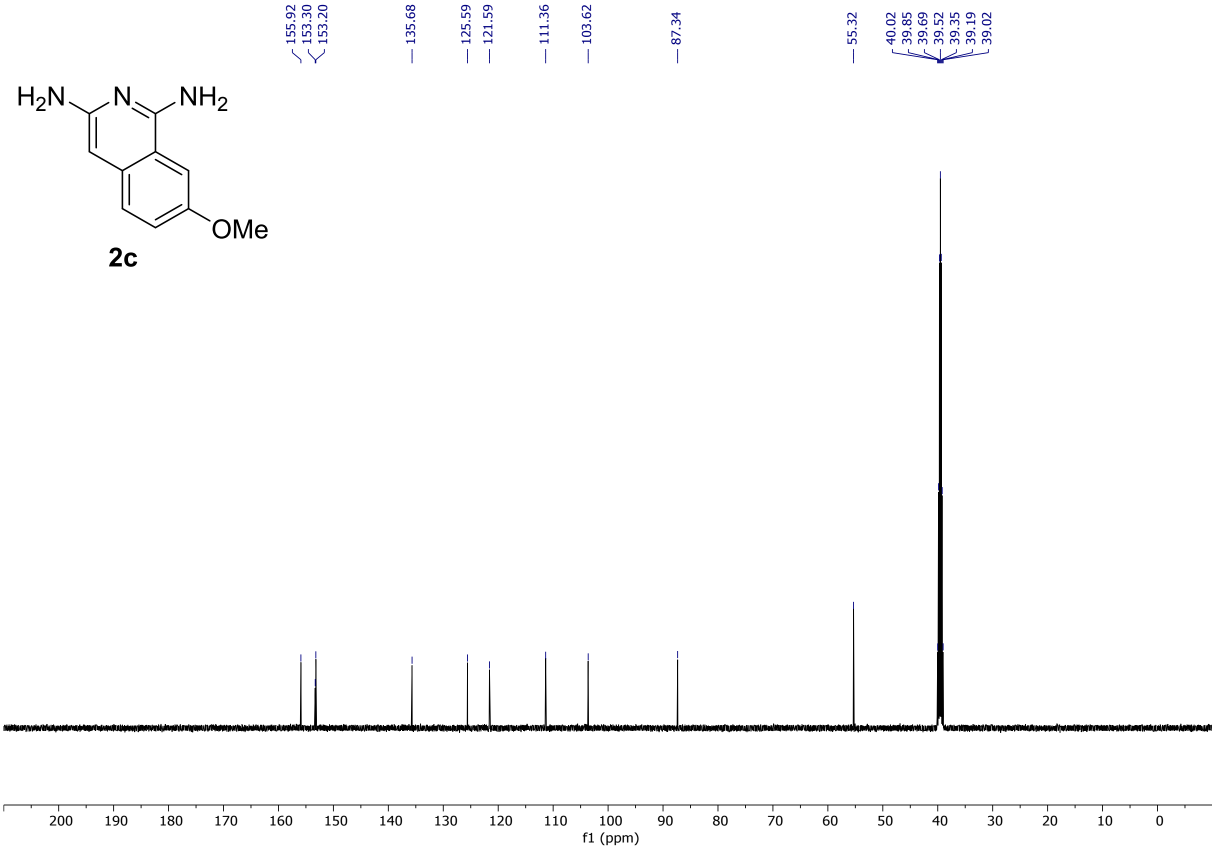

^{1}H NMR (500 MHz, DMSO-d$_6$, 298K)

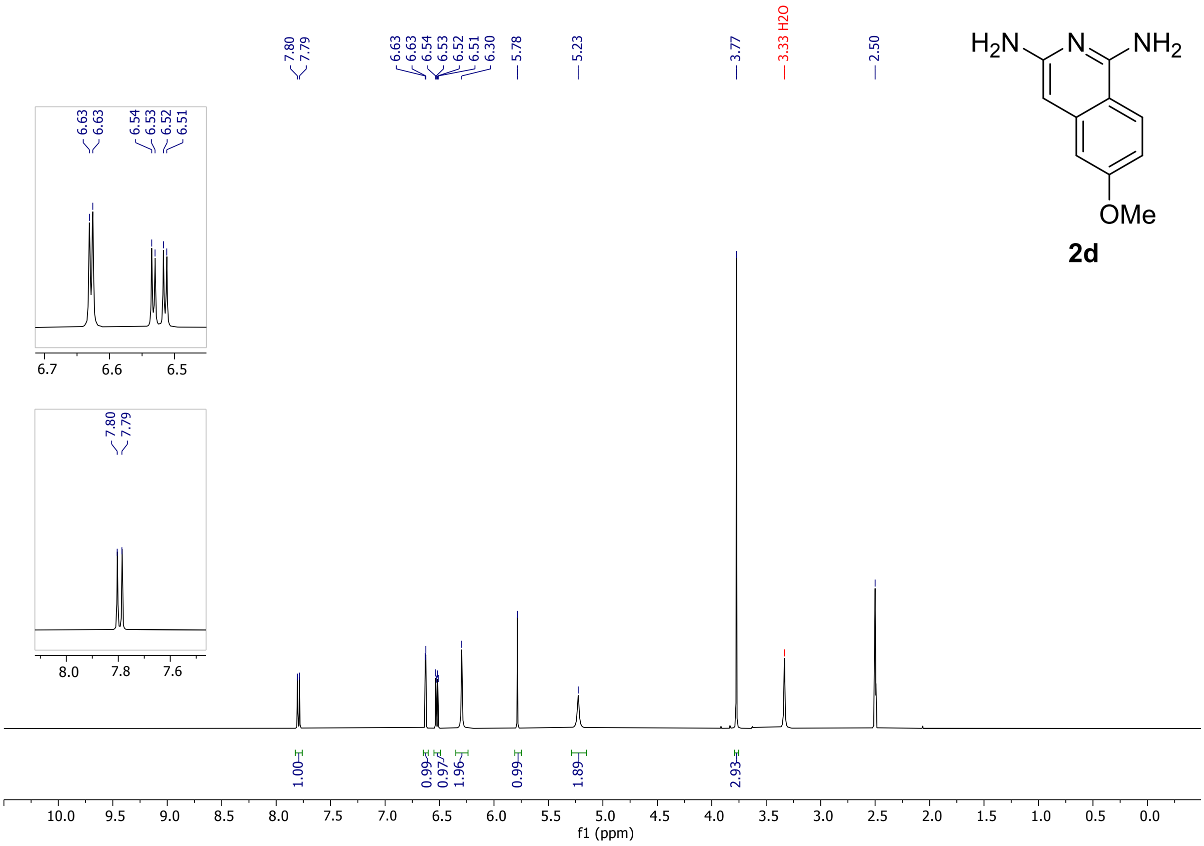

2d

^{13}C{^{1}H} NMR (126 MHz, DMSO-d$_6$, 298K)

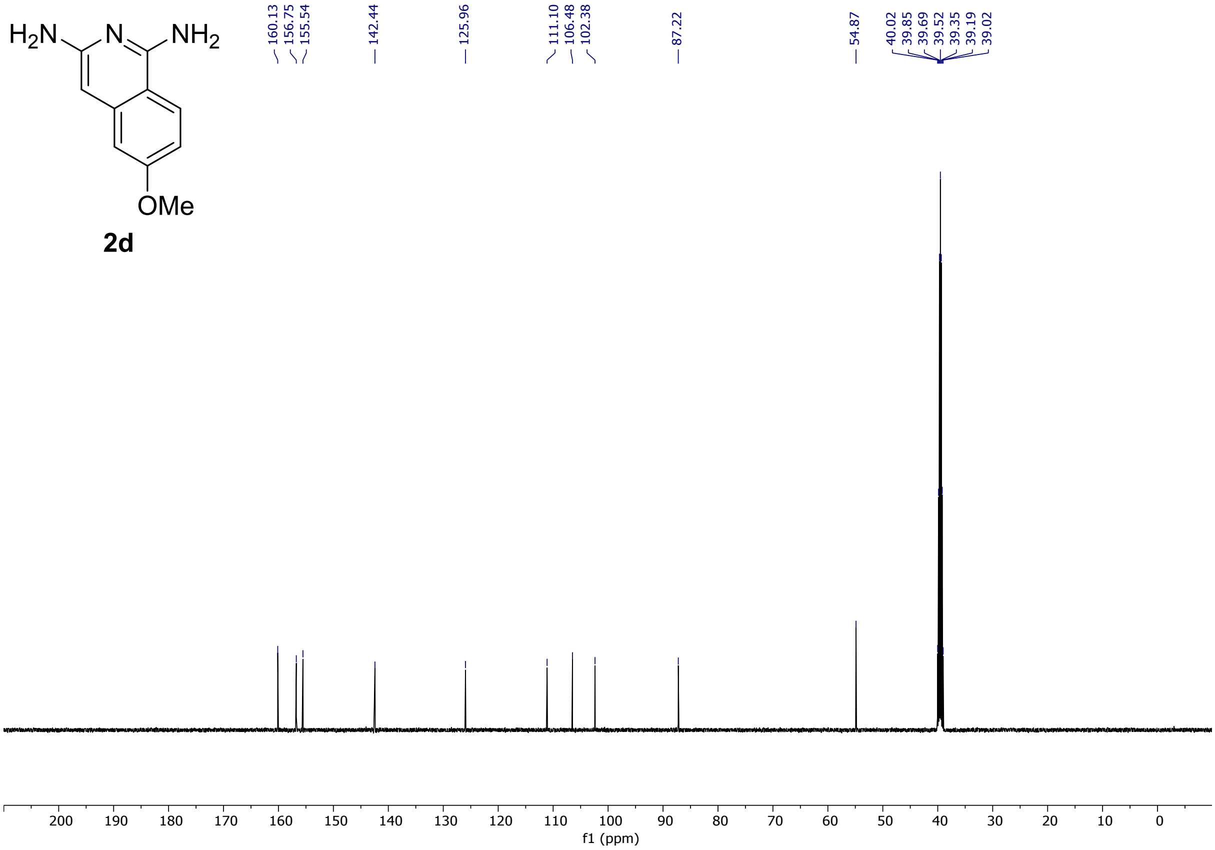

^{1}H NMR (500 MHz, DMSO-d$_6$, 298K)

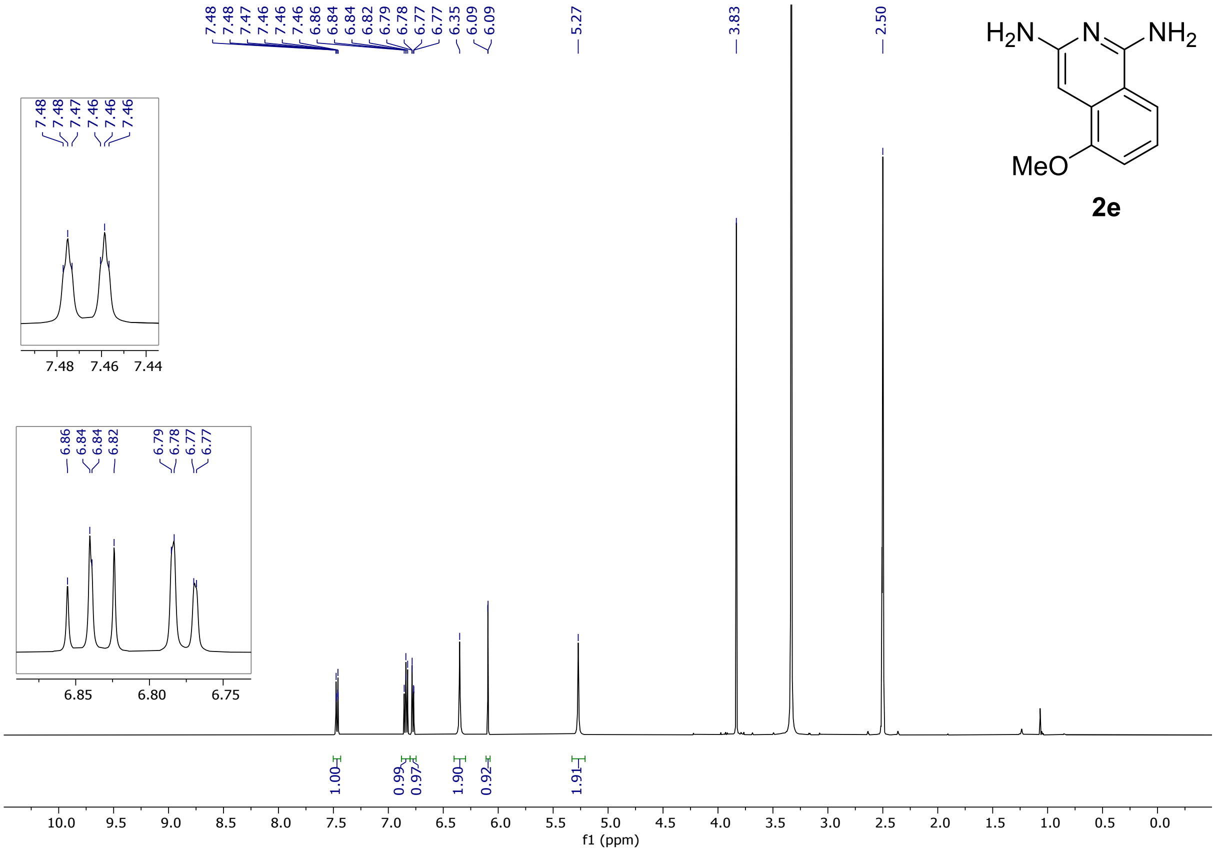

$^{13}C\{^{1}H\}$ NMR (126 MHz, DMSO-d$_6$, 298K)

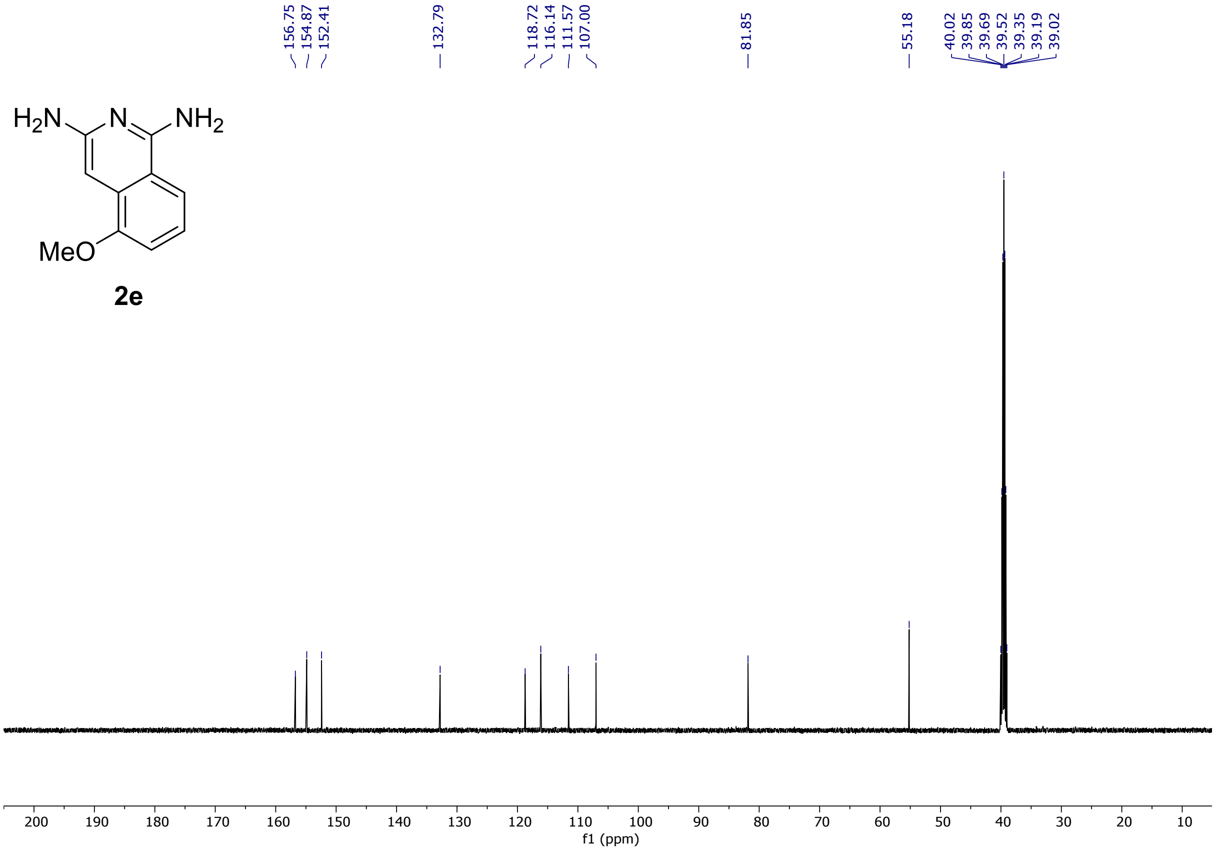

^{1}H NMR (500 MHz, DMSO-d_6, 298K)

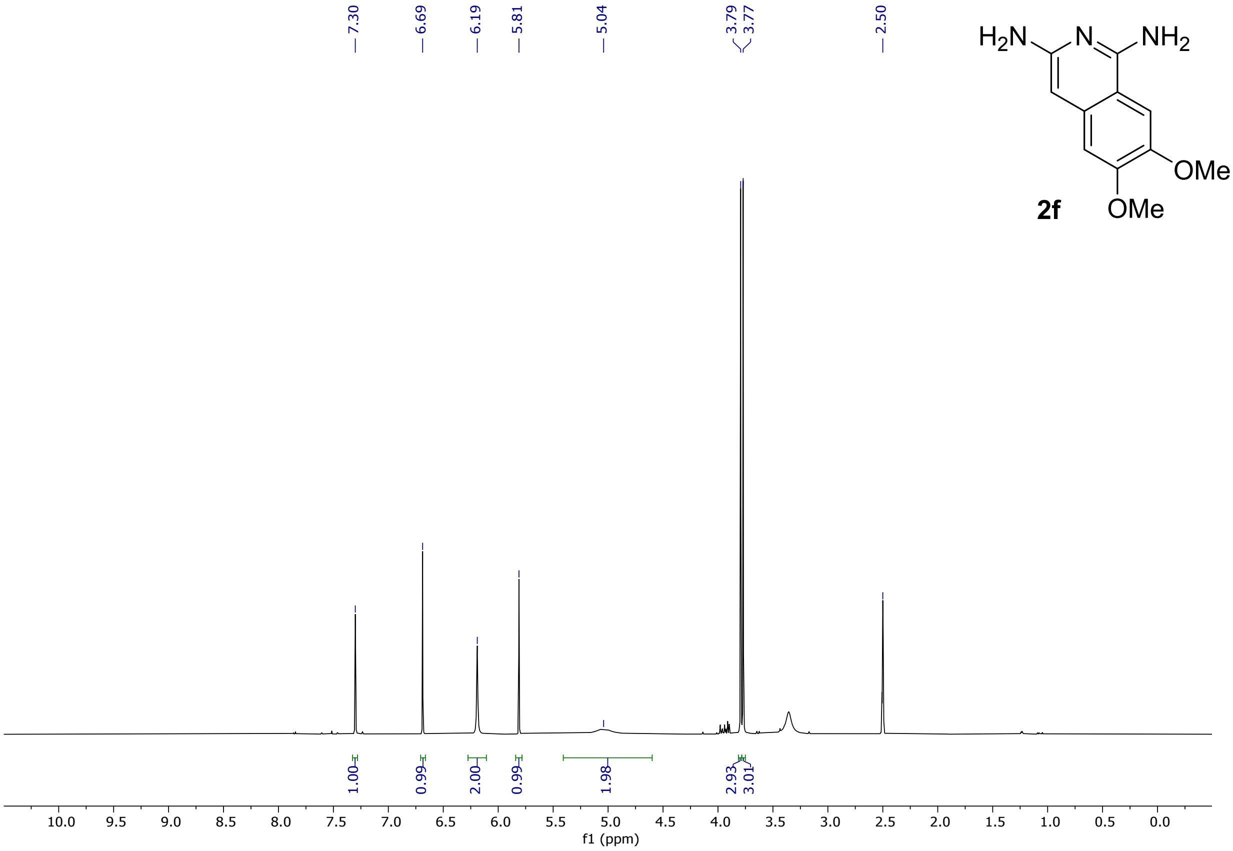

$^{13}C\{^{1}H\}$ NMR (126 MHz, DMSO-d_6, 298K)

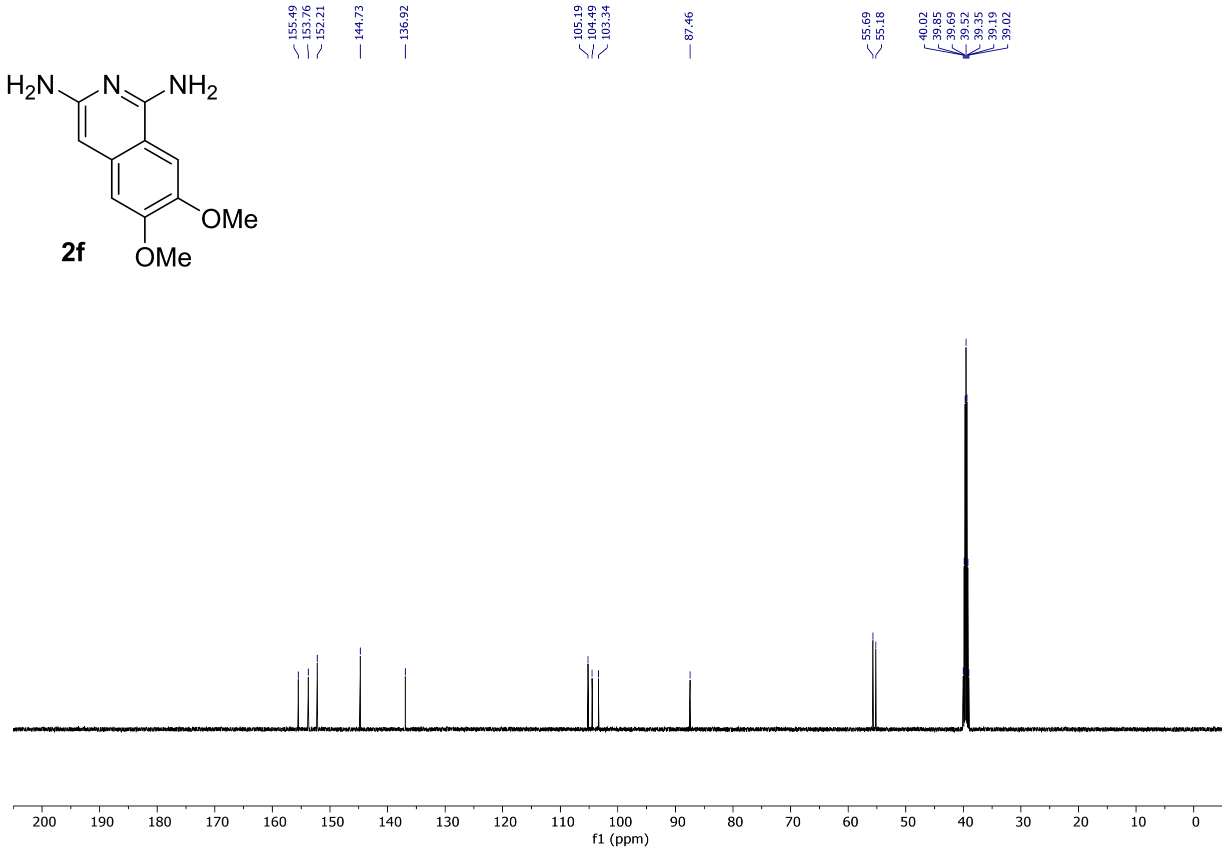

^{1}H NMR (500 MHz, DMSO-d_6, 298K)

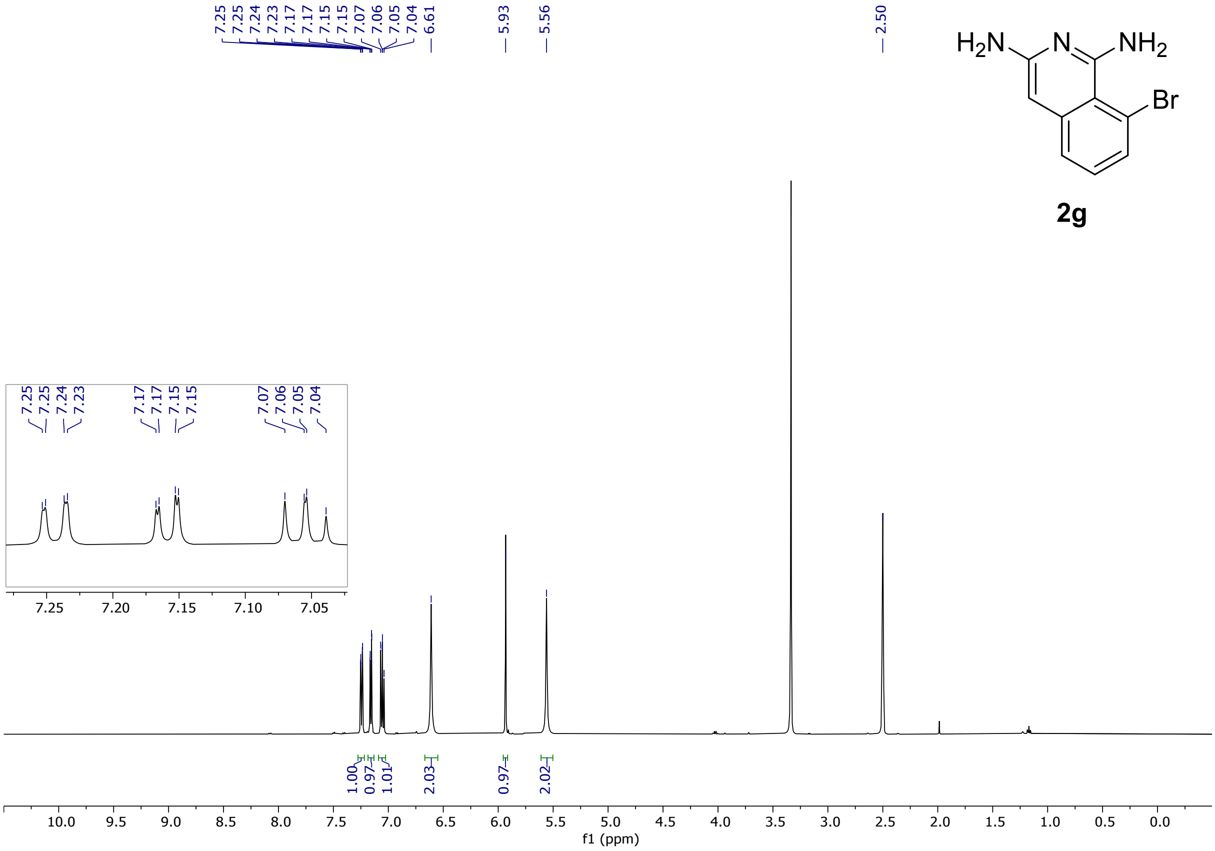

$^{13}C\{^{1}H\}$ NMR (126 MHz, DMSO-d_6, 298K)

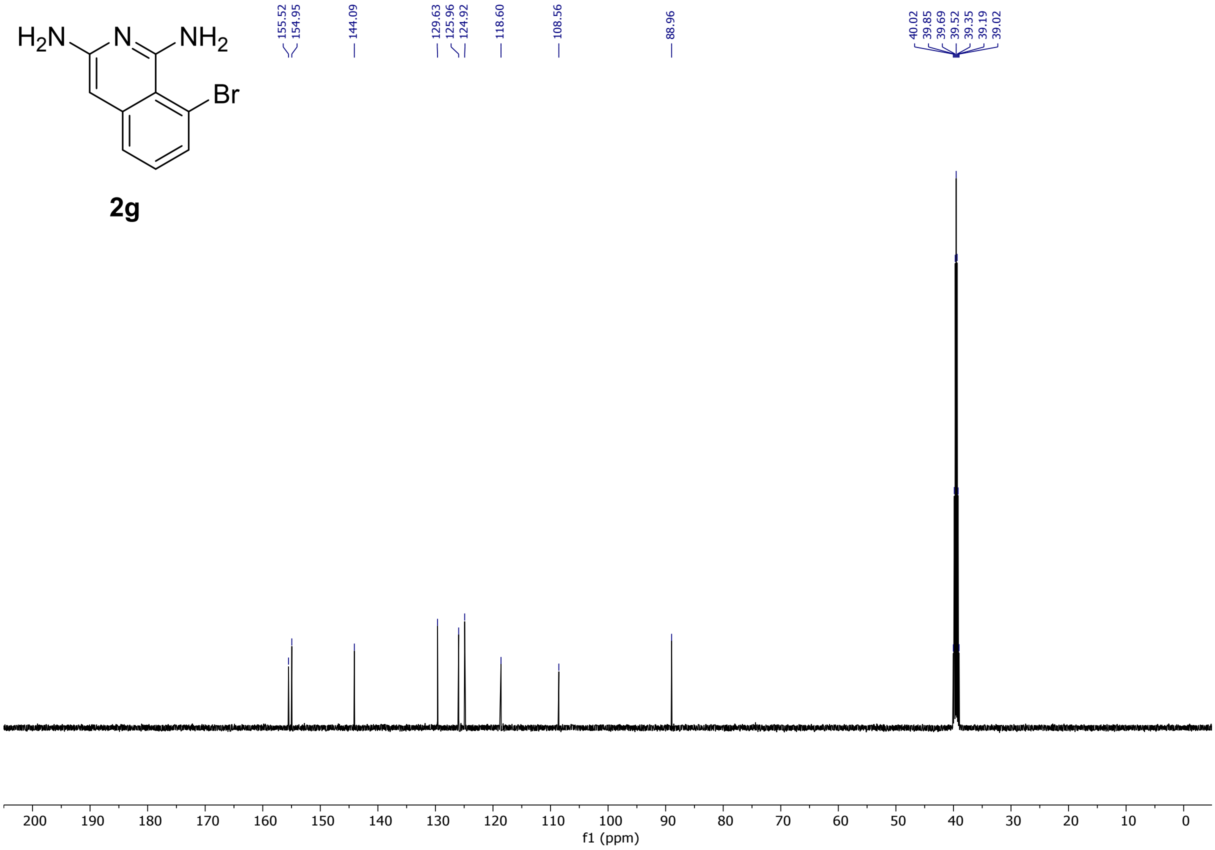

^{1}H NMR (500 MHz, DMSO-d_6, 298K)

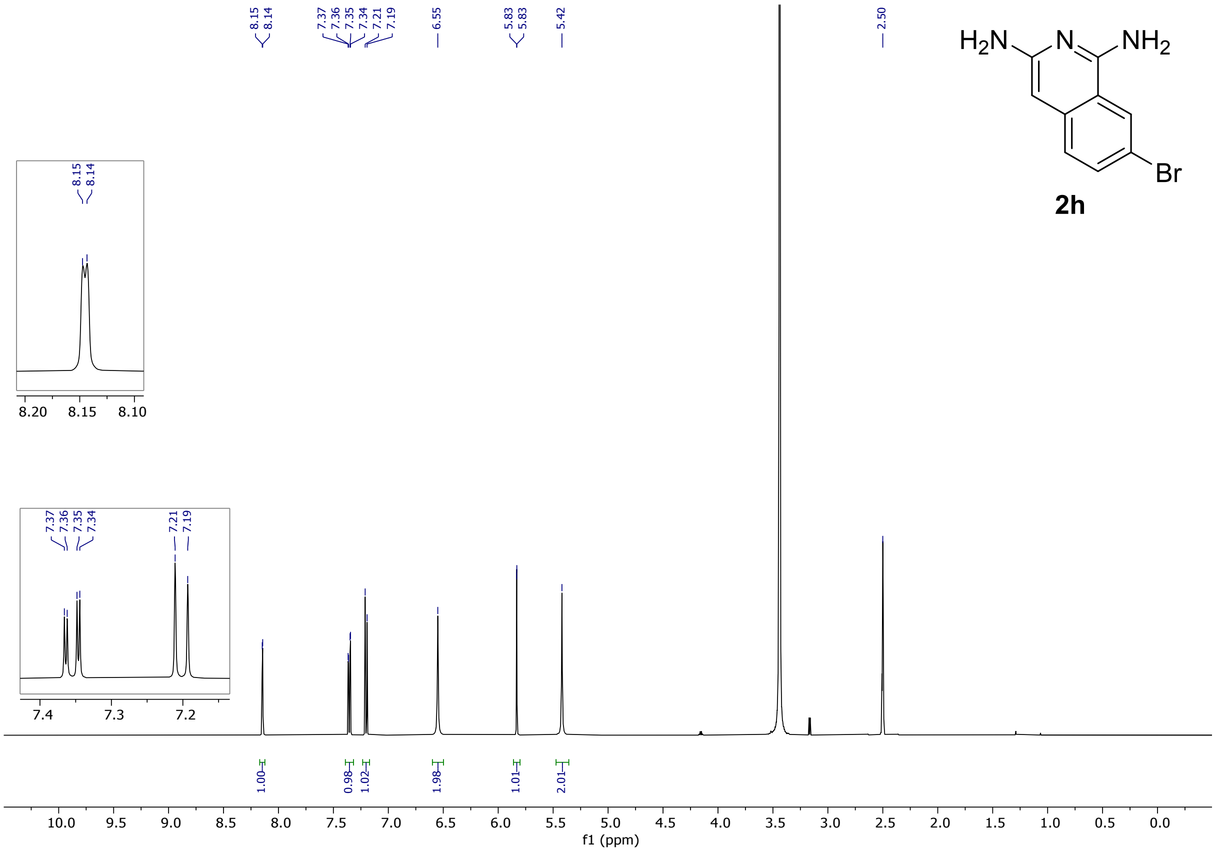

2h

^{13}C{^{1}H} NMR (126 MHz, DMSO-d_6, 298K)

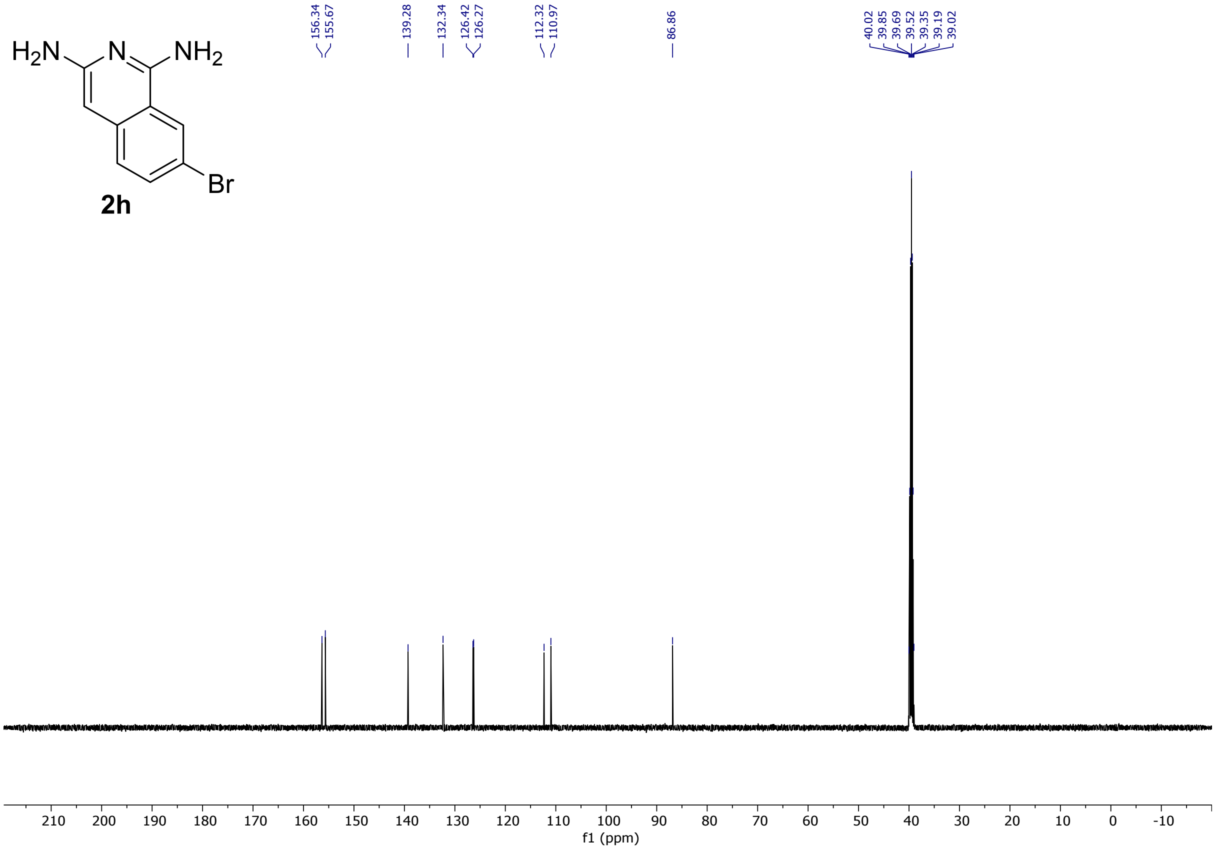

2h

^{1}H NMR (500 MHz, DMSO-d_6, 298K)

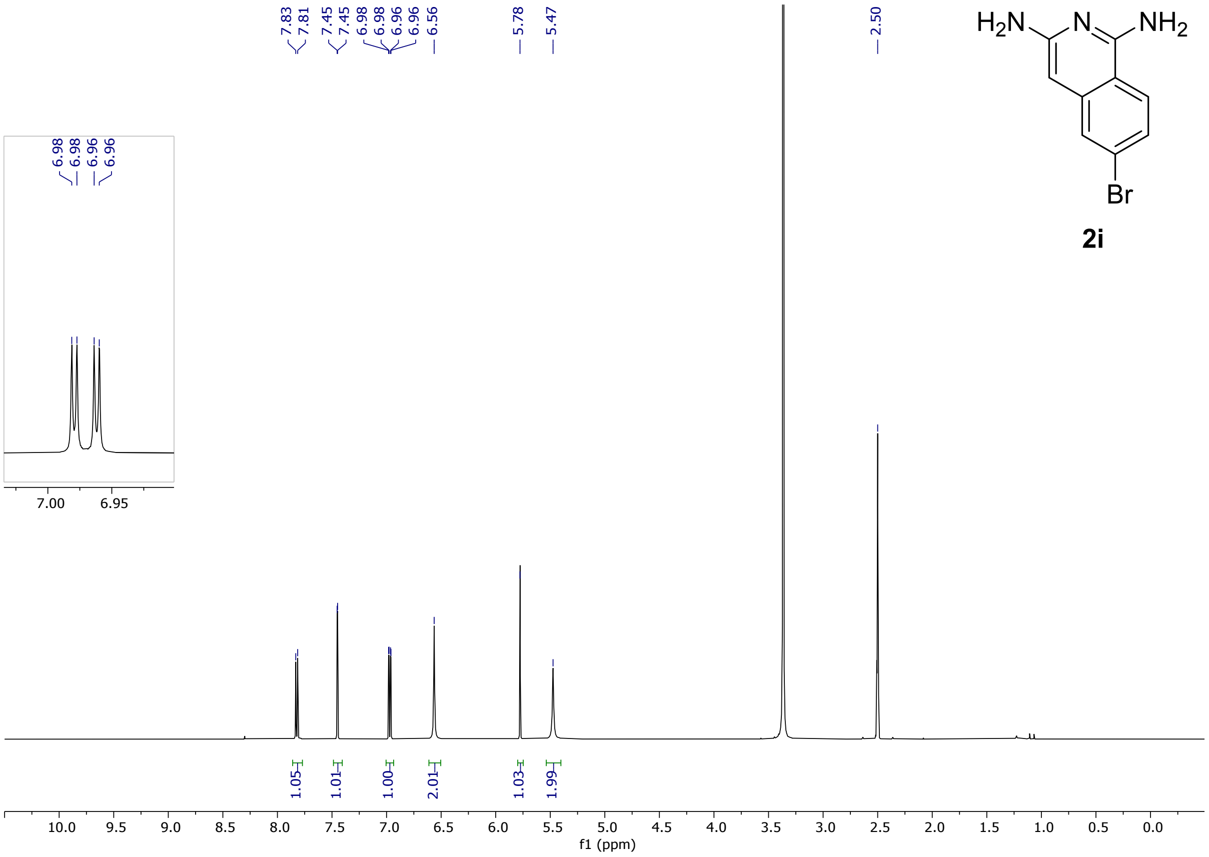

$^{13}C\{^{1}H\}$ NMR (126 MHz, DMSO-d_6, 298K)

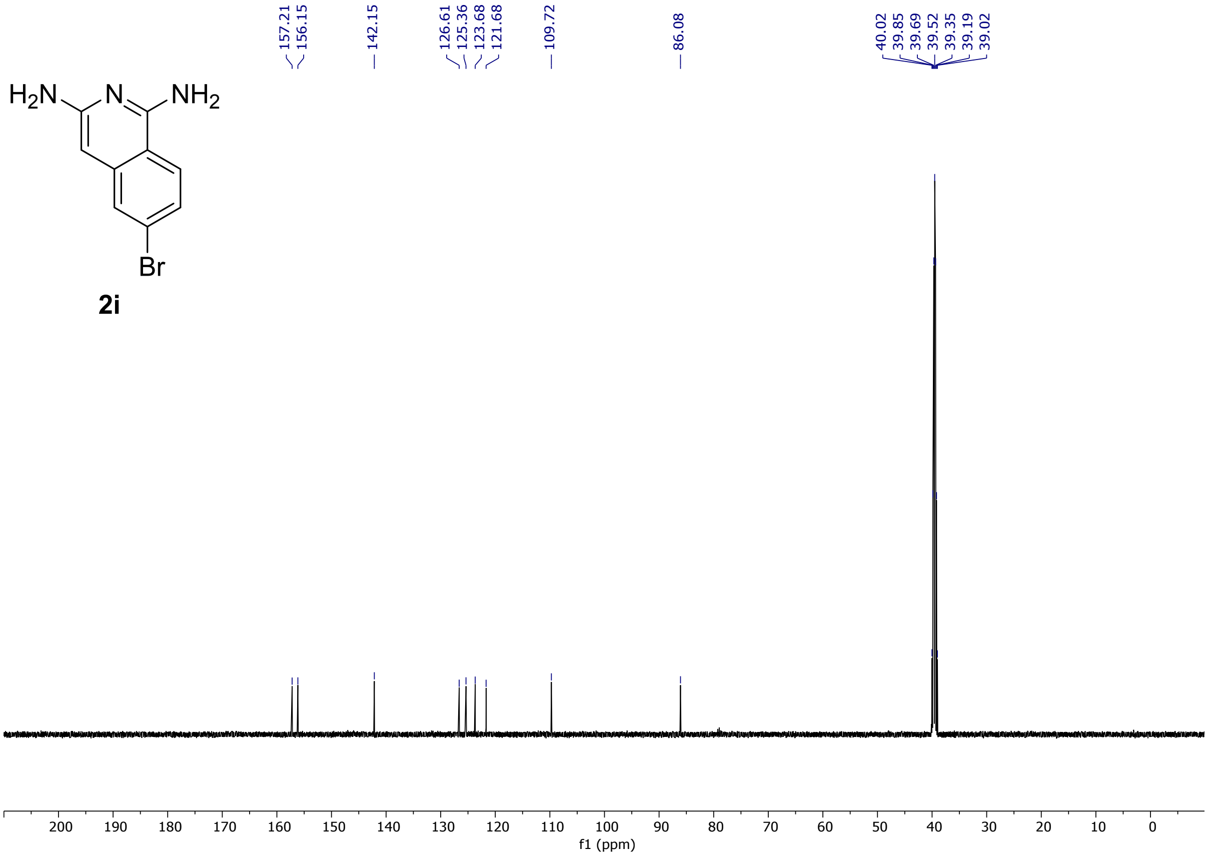

^{1}H NMR (500 MHz, DMSO-d_6, 298K)

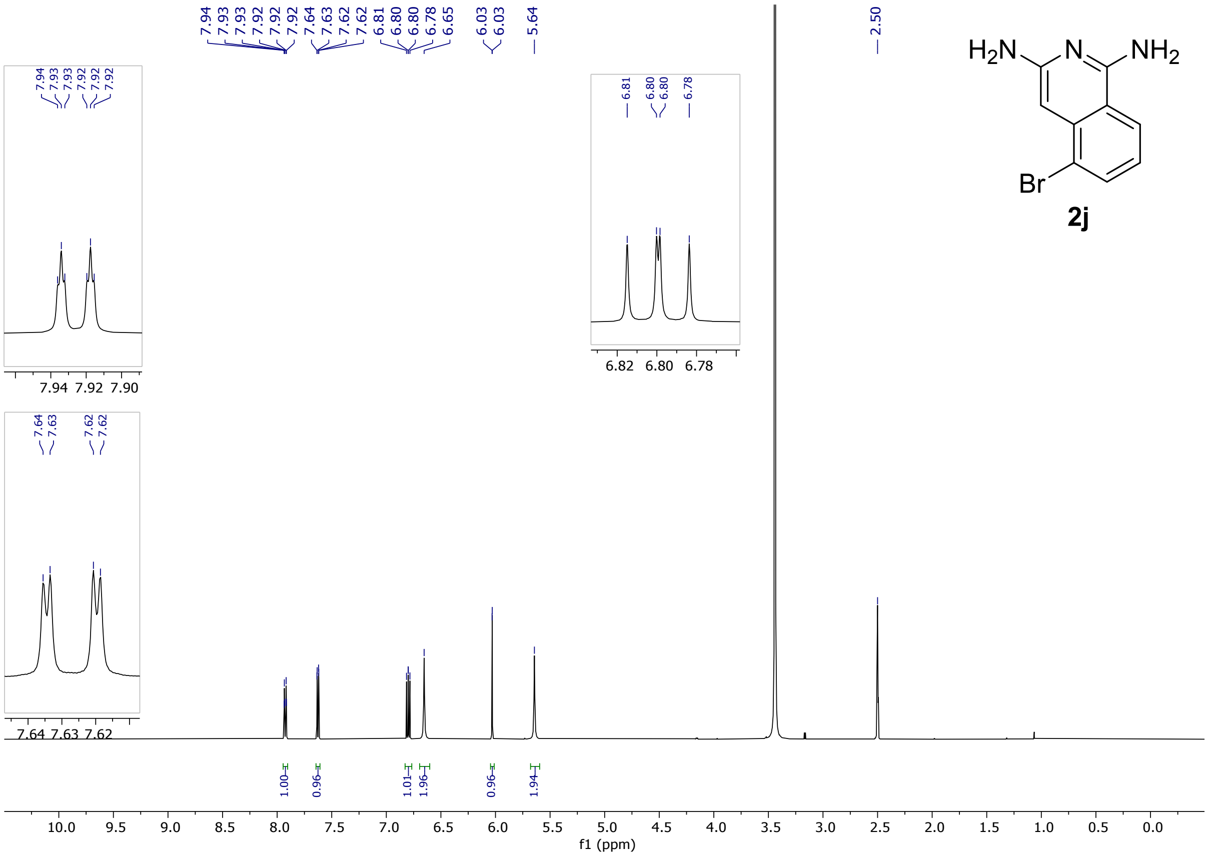

$^{13}C\{^{1}H\}$ NMR (126 MHz, DMSO-d_6, 298K)

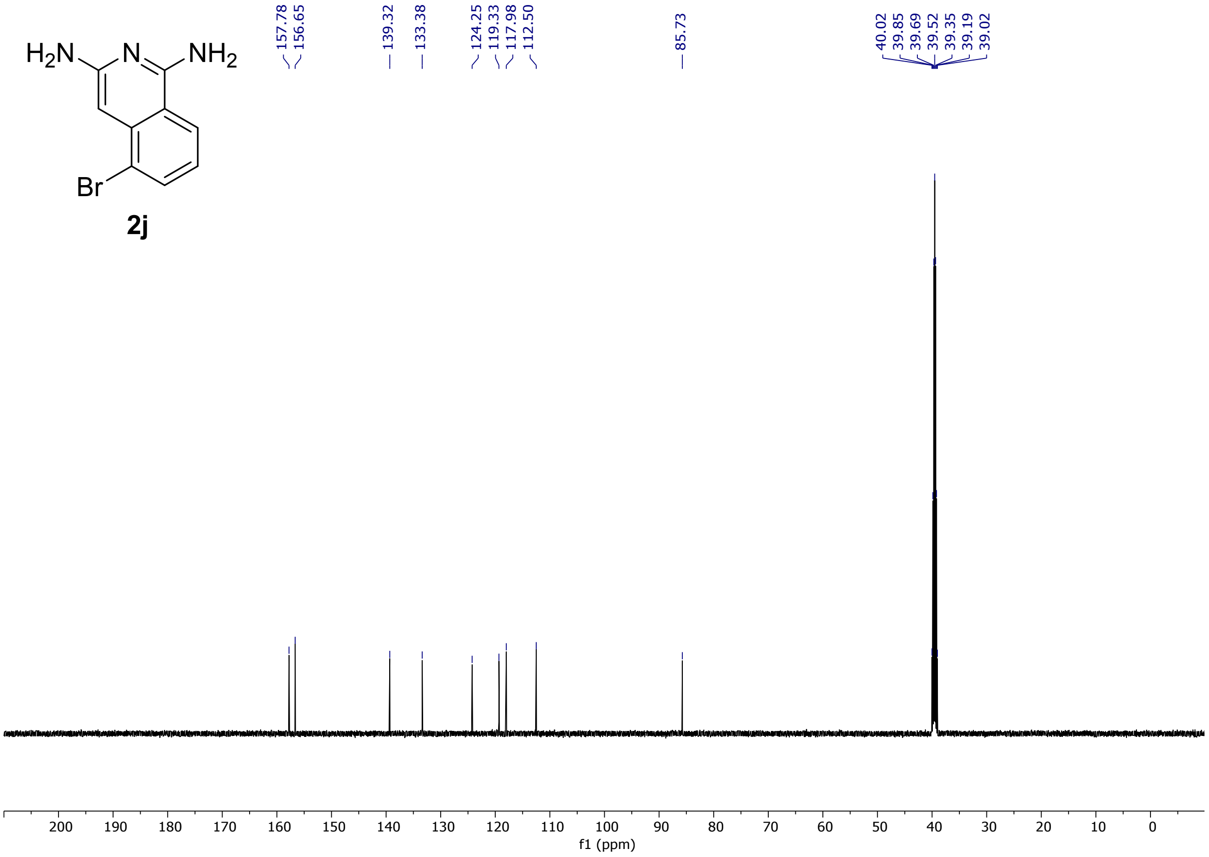

^{1}H NMR (500 MHz, DMSO-d_6, 298K)

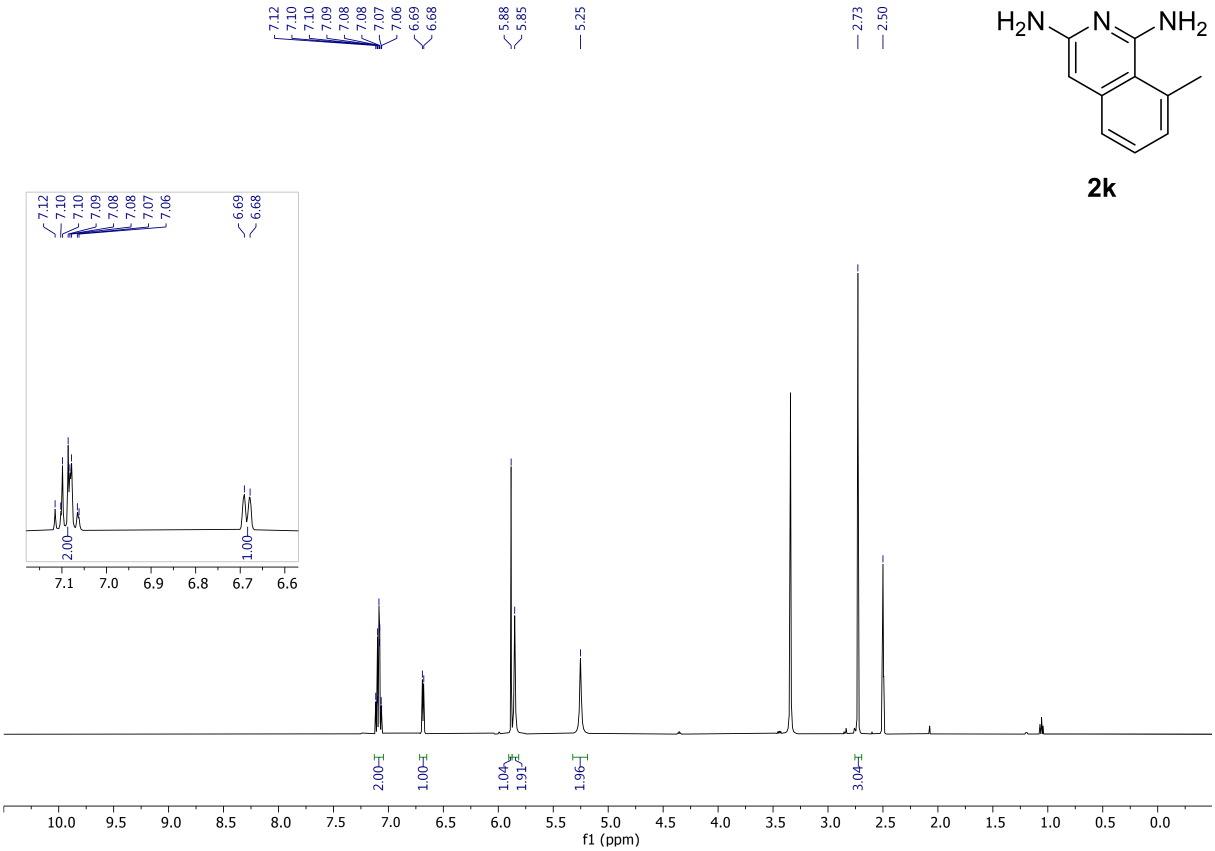

$^{13}C\{^{1}H\}$ NMR (126 MHz, DMSO-d_6, 298K)

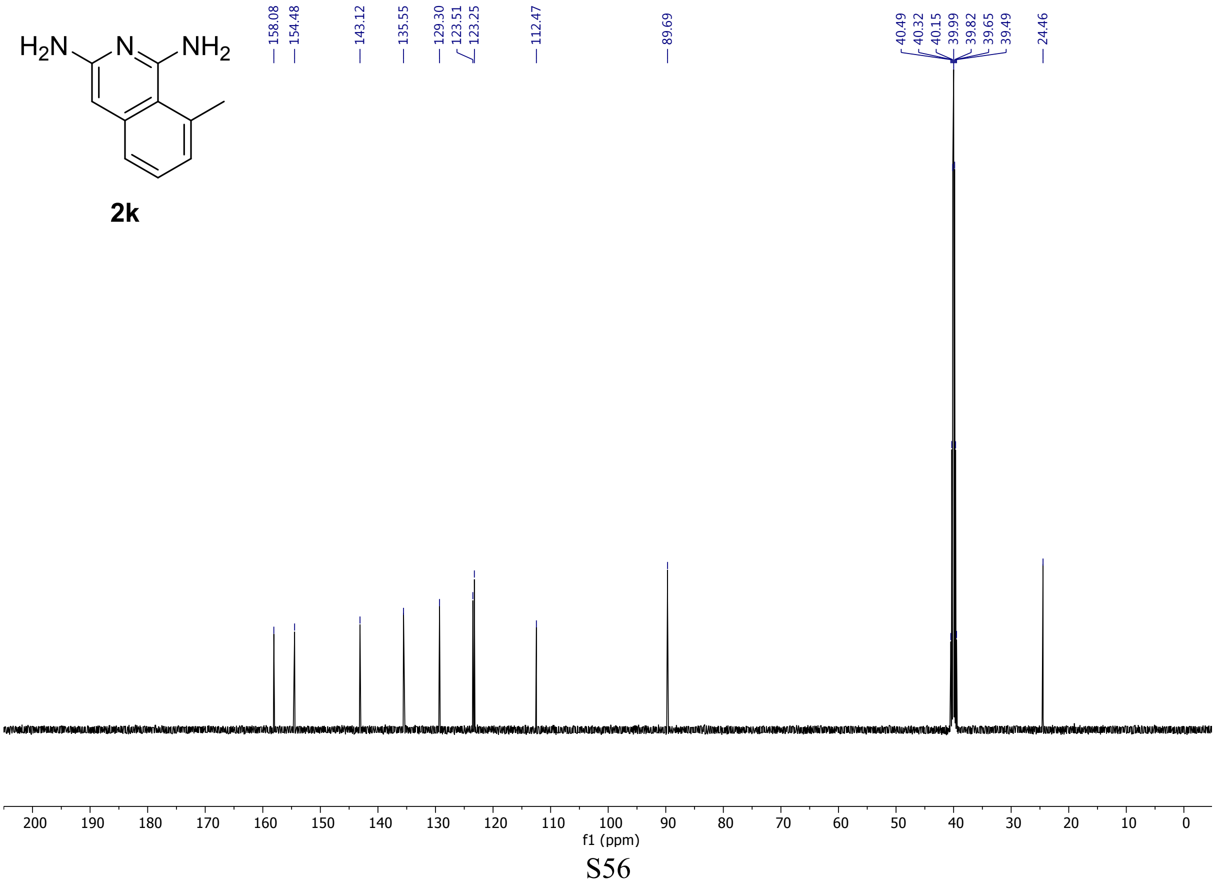

^{1}H NMR (500 MHz, DMSO-d$_6$, 298K)

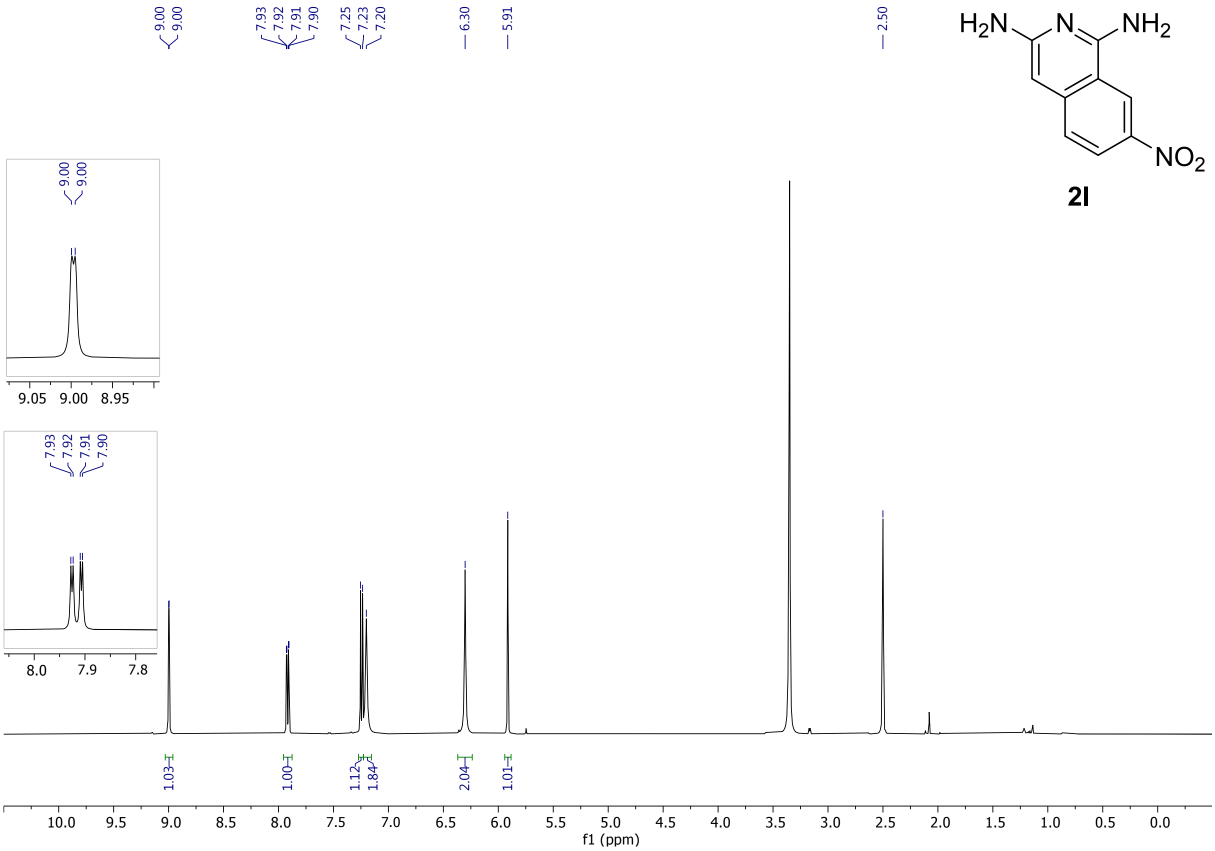

^{13}C{^{1}H} NMR (126 MHz, DMSO-d$_6$, 298K)

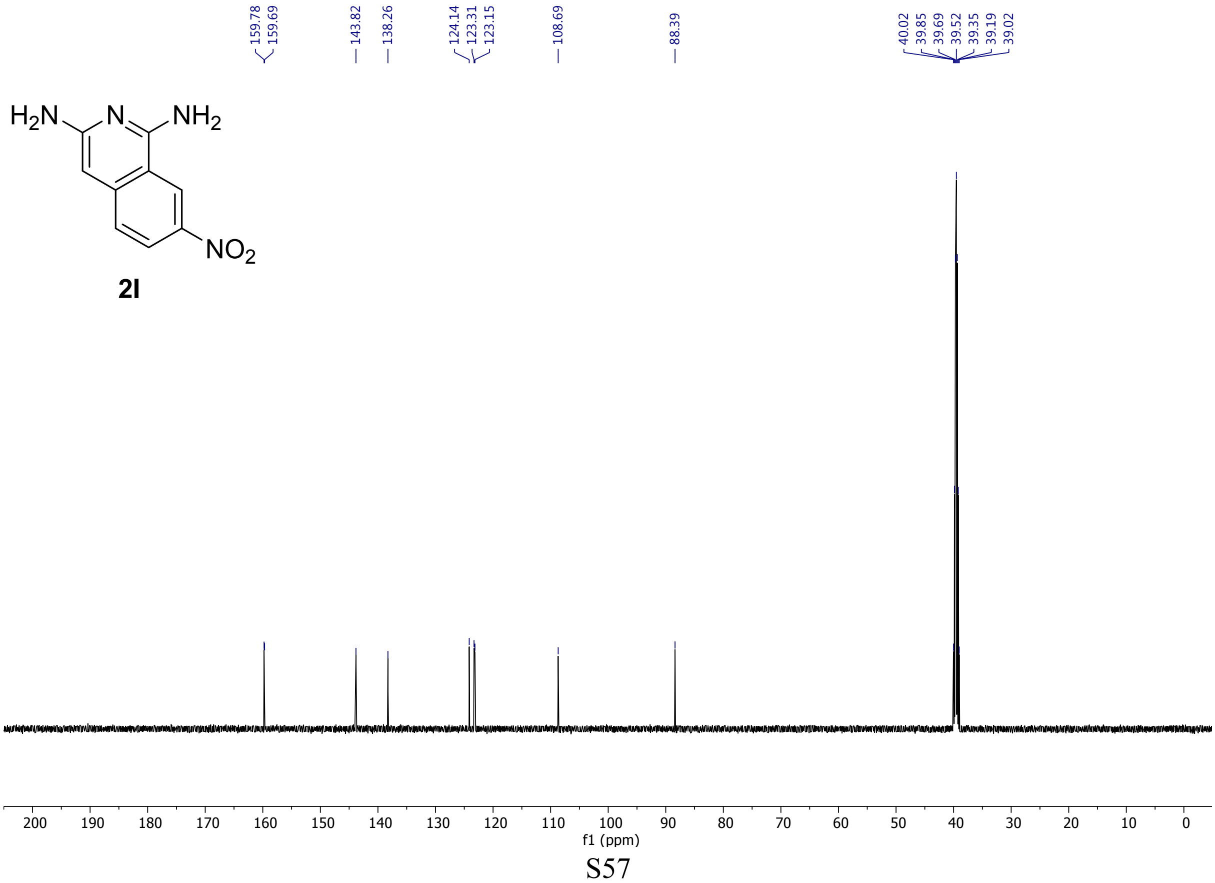

^{1}H NMR (500 MHz, DMSO-d$_6$, 298K)

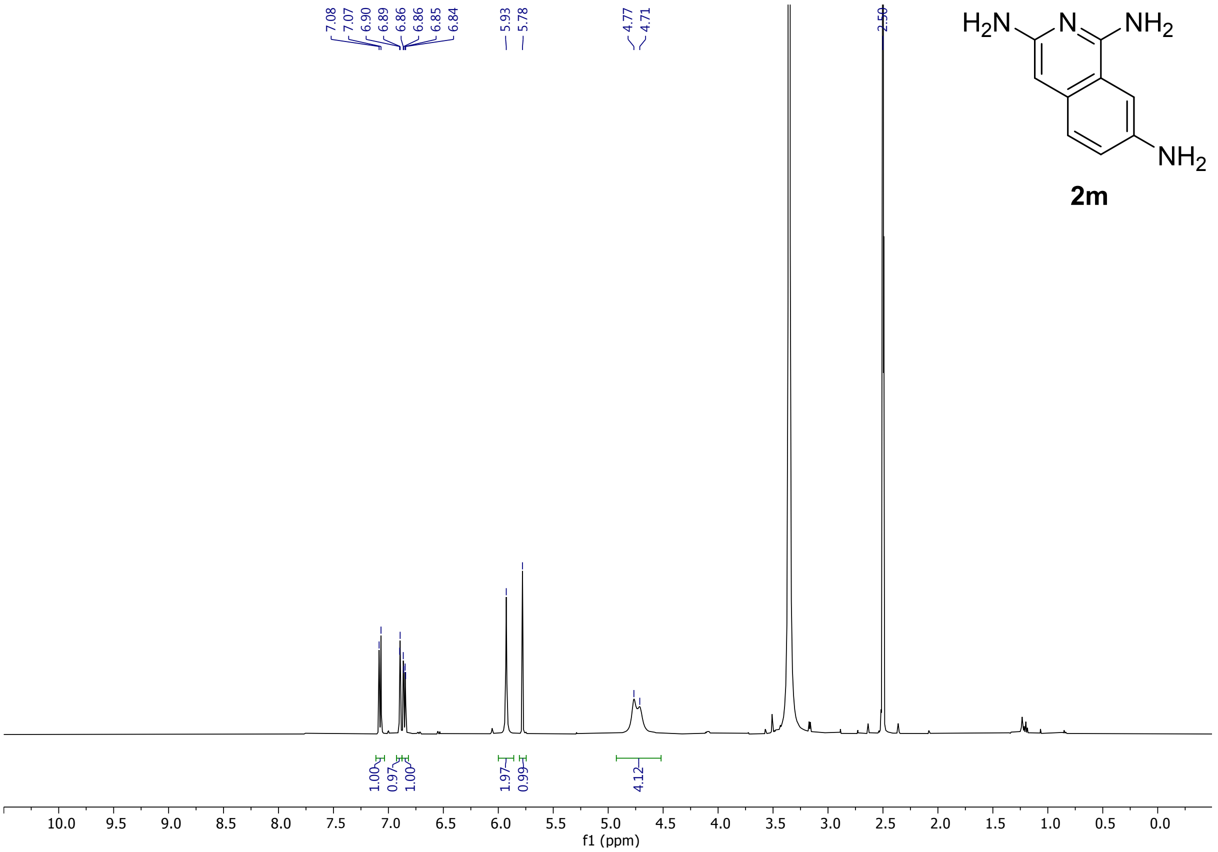

^{13}C{^{1}H} NMR (126 MHz, DMSO-d$_6$, 298K)

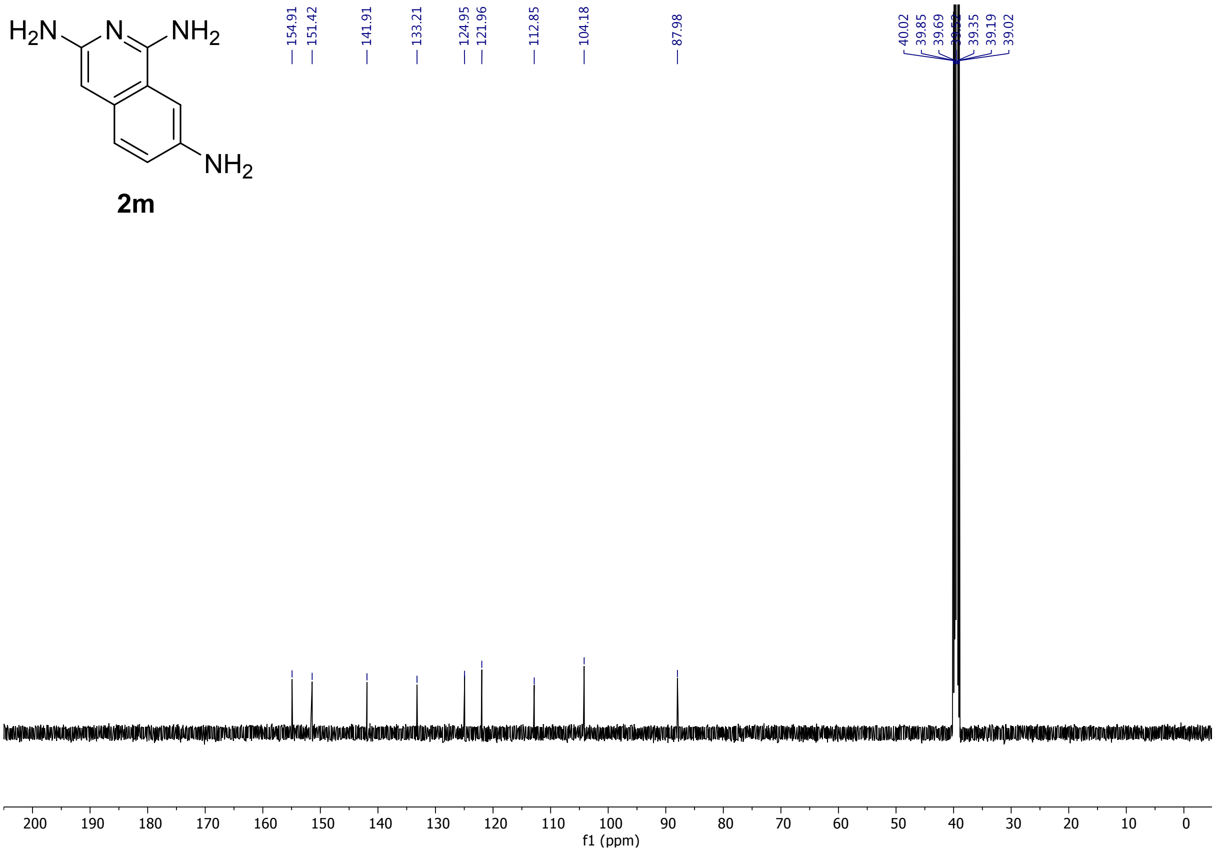

^{1}H NMR (500 MHz, DMSO-d_6, 298K)

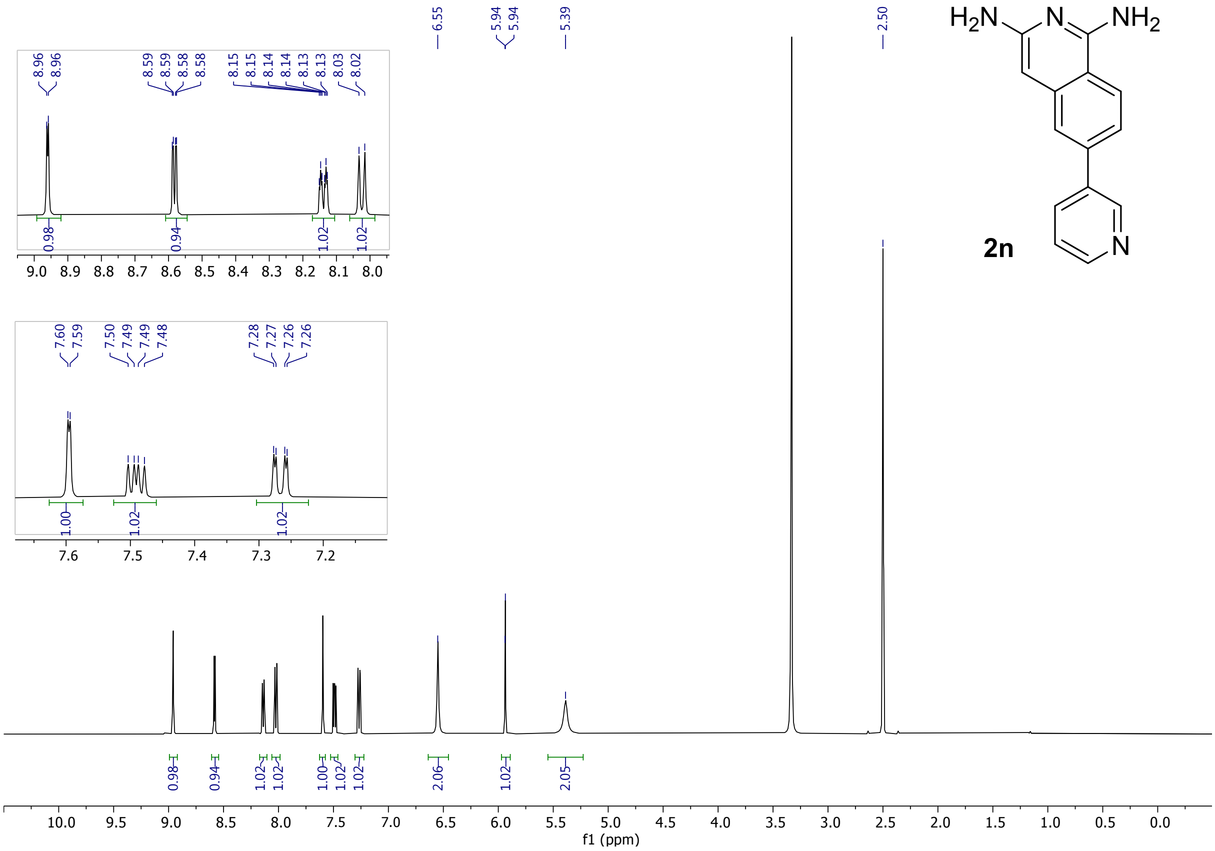

^{13}C{^{1}H} NMR (126 MHz, DMSO-d_6, 298K)

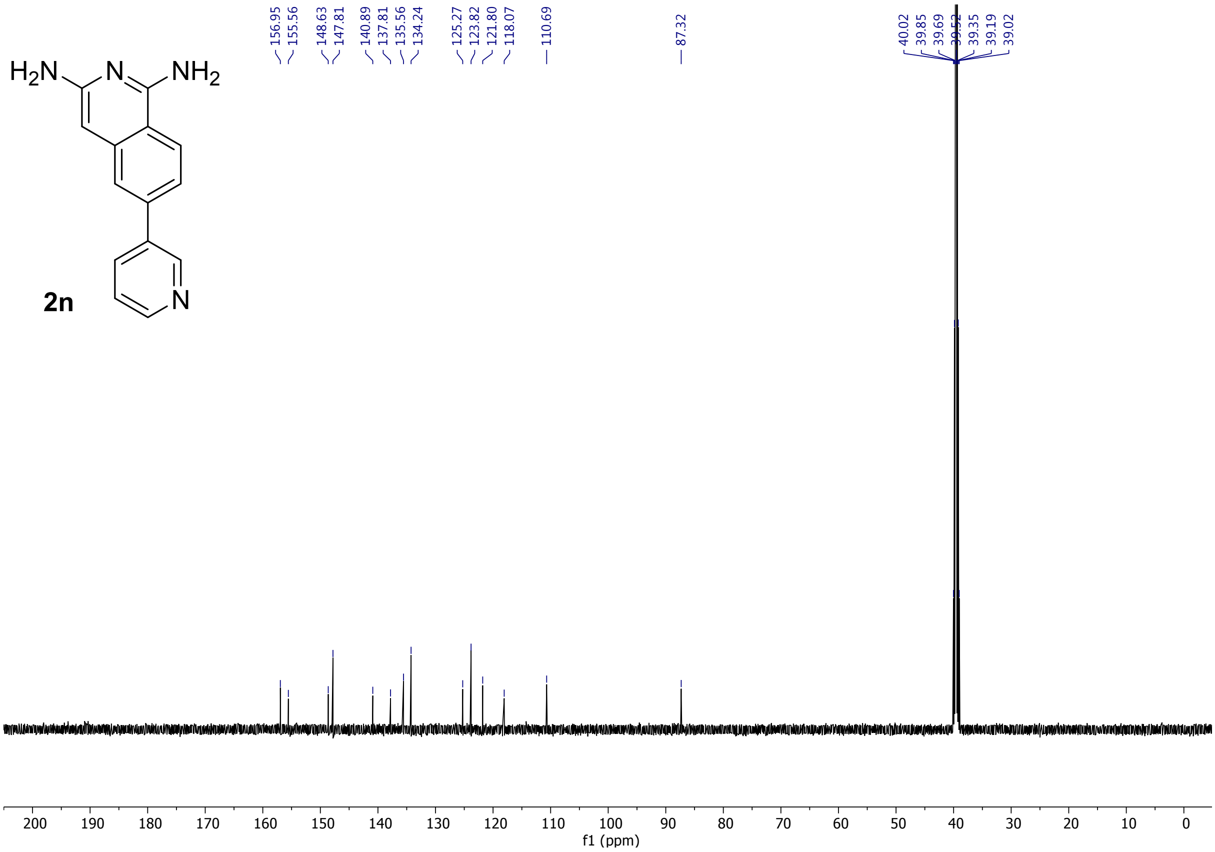

^{1}H NMR (500 MHz, DMSO-d$_6$, 298K)

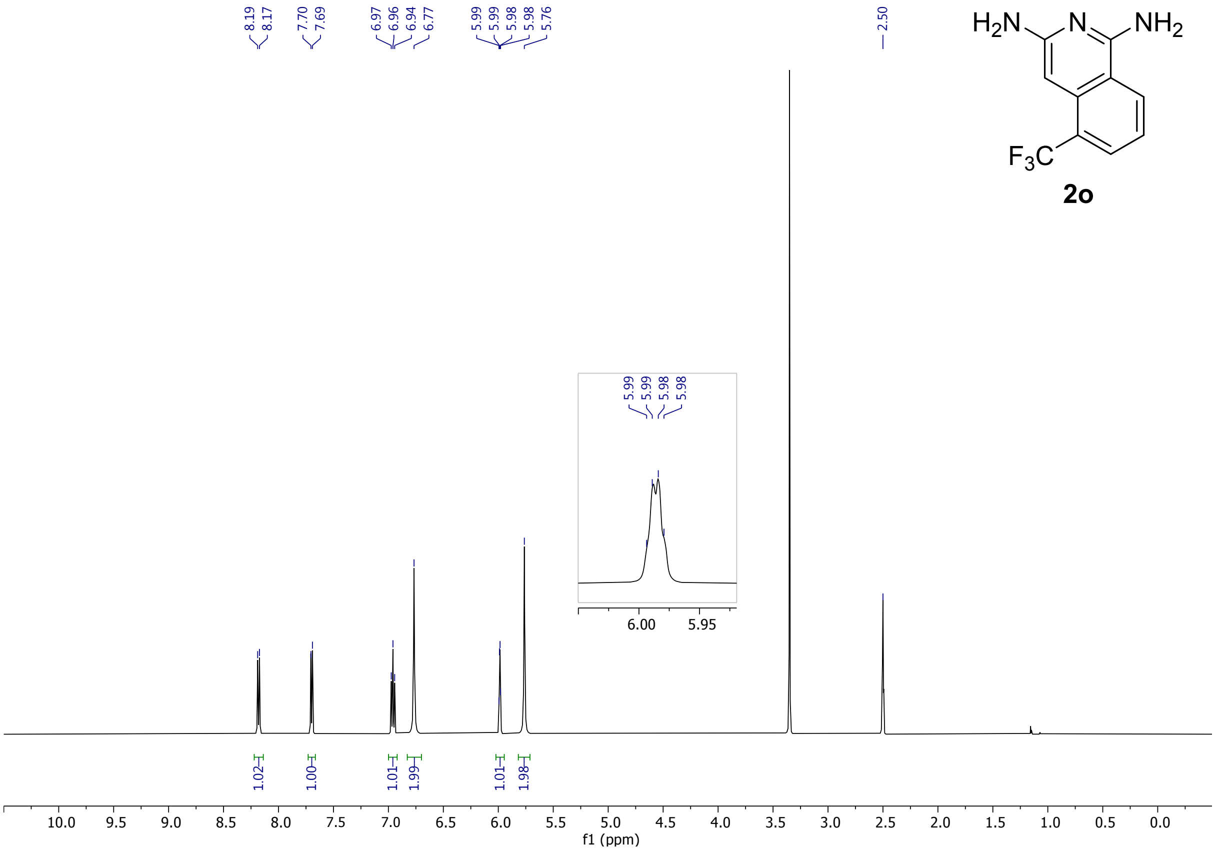

$^{13}C\{^{1}H\}$ NMR (126 MHz, DMSO-d$_6$, 298K)

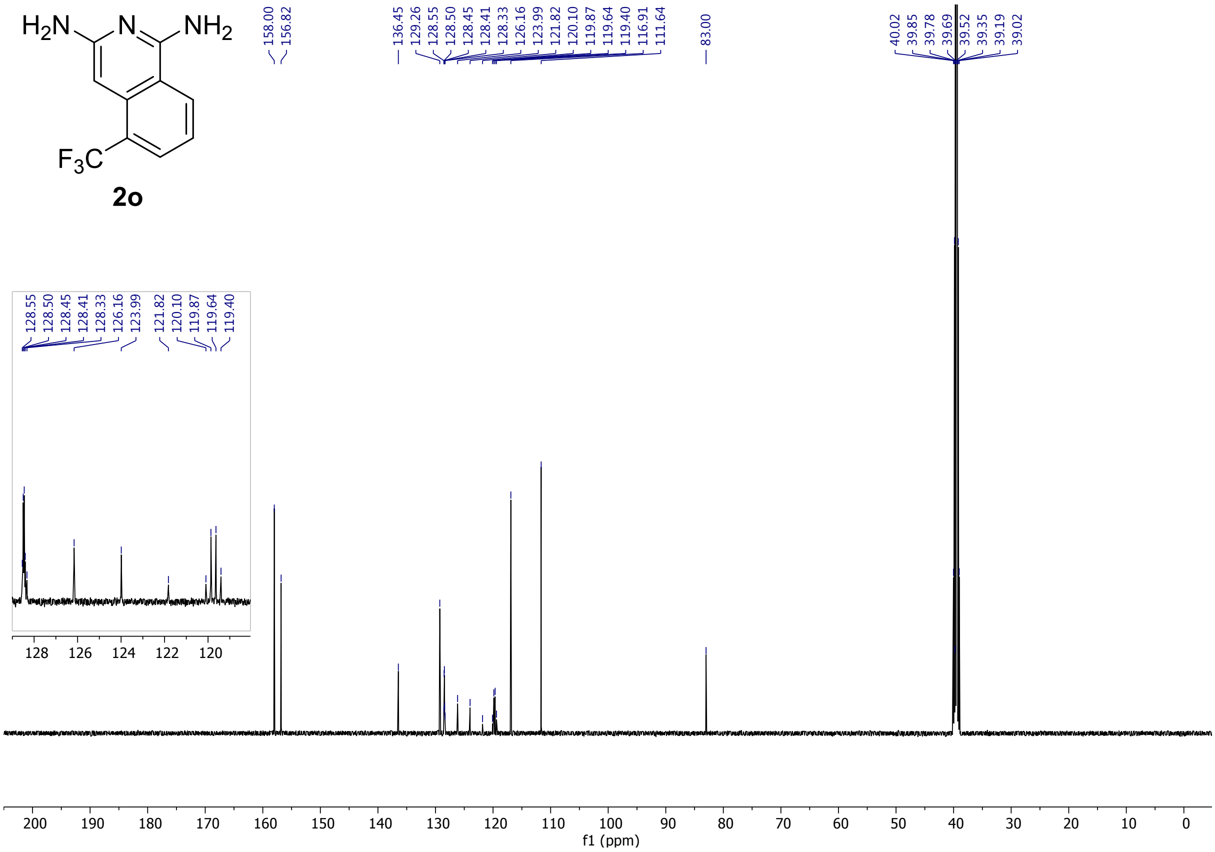

^1H NMR (500 MHz, DMSO-d$_6$, 298K)

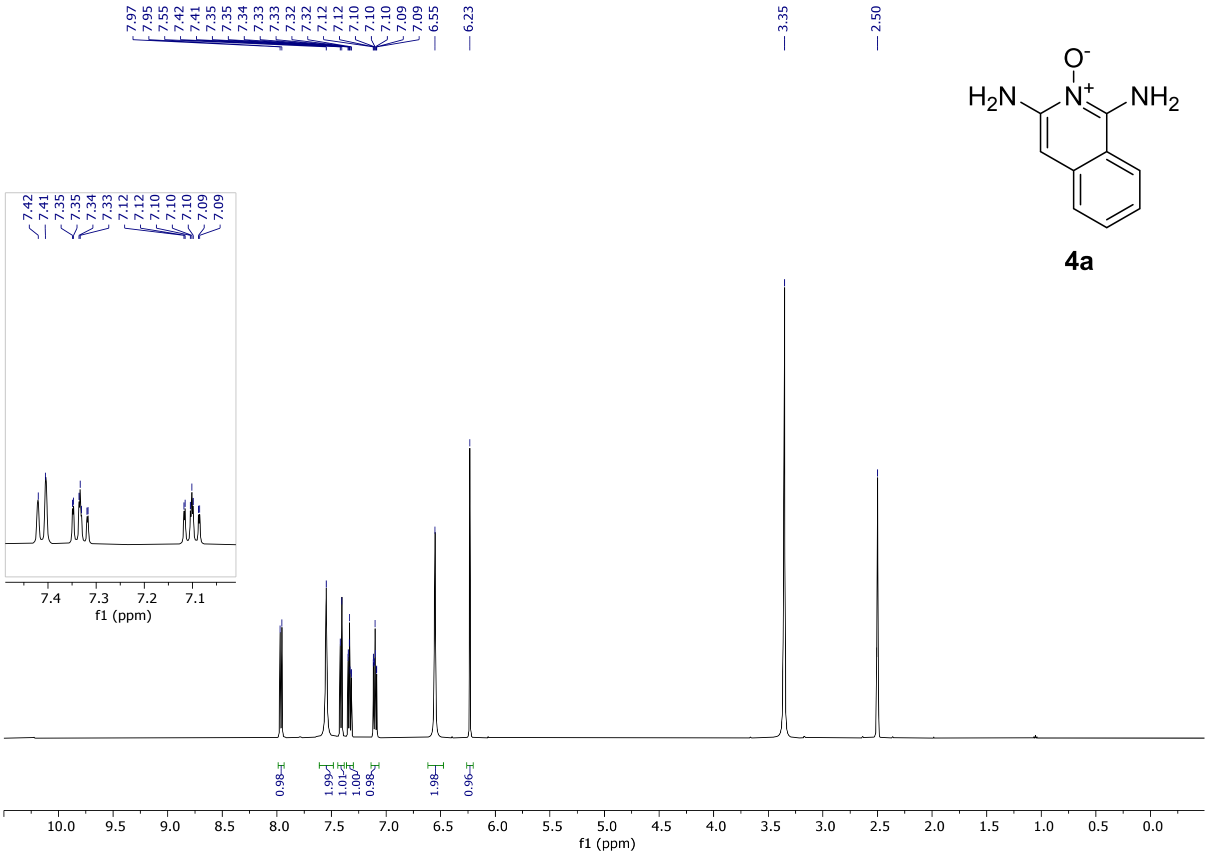

$^{13}C\{^1H\}$ NMR (126 MHz, DMSO-d$_6$, 298K)

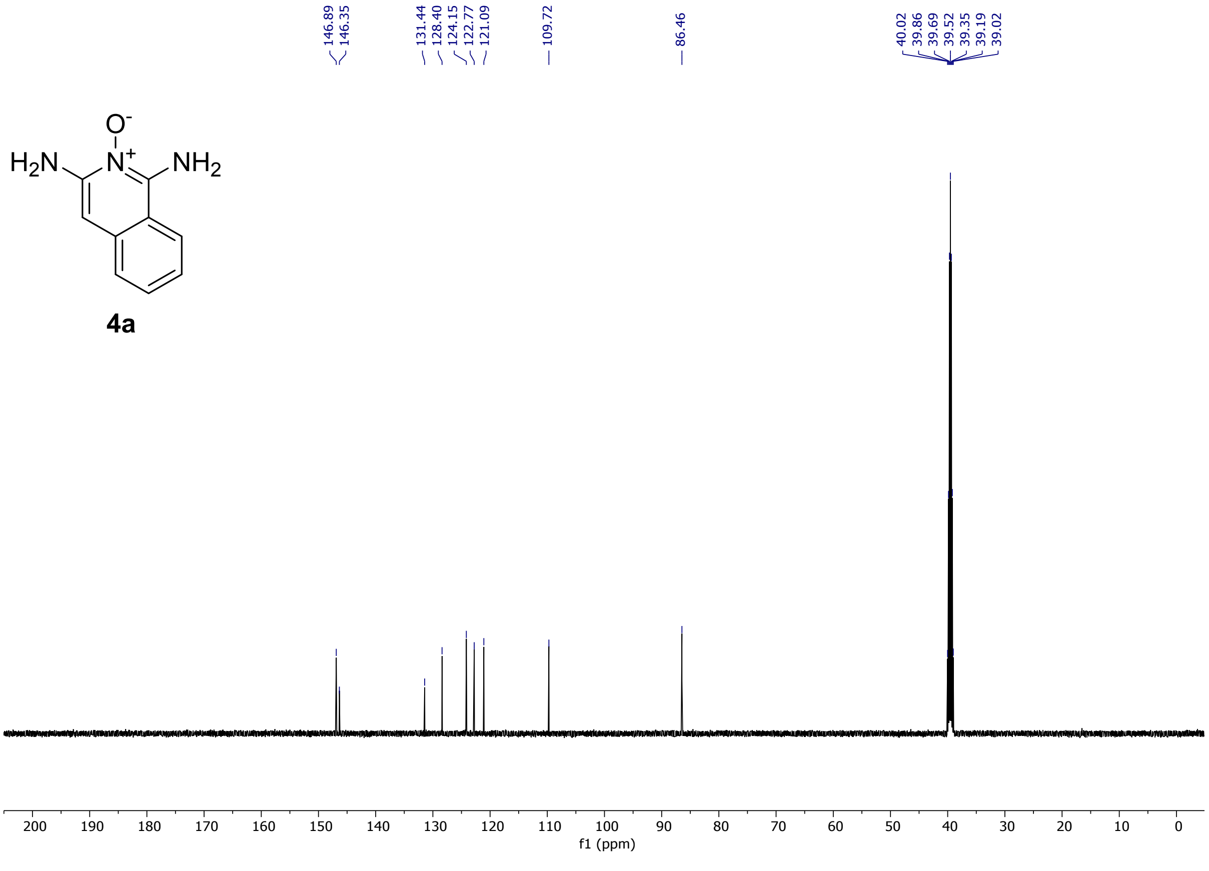

^{1}H NMR (500 MHz, DMSO-d$_6$, 298K)

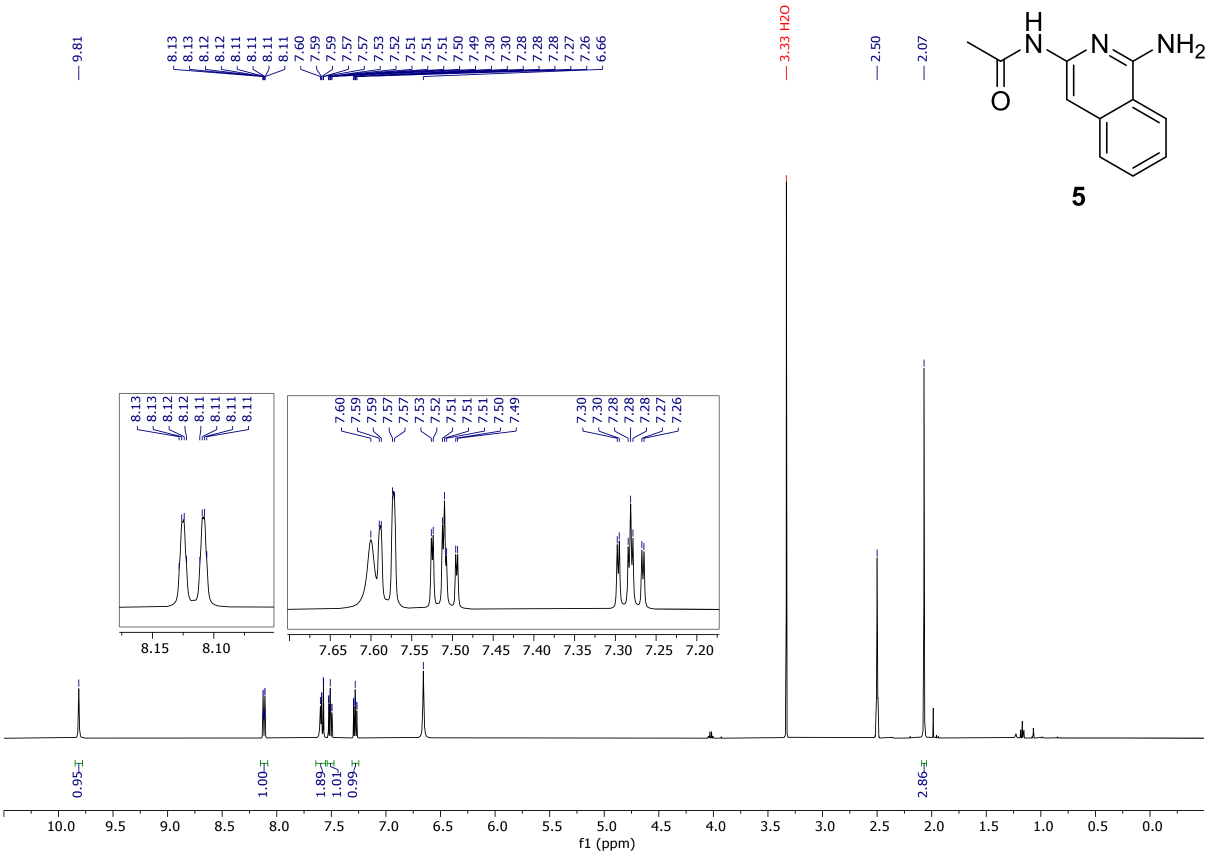

^{13}C{^{1}H} NMR (126 MHz, DMSO-d$_6$, 298K)

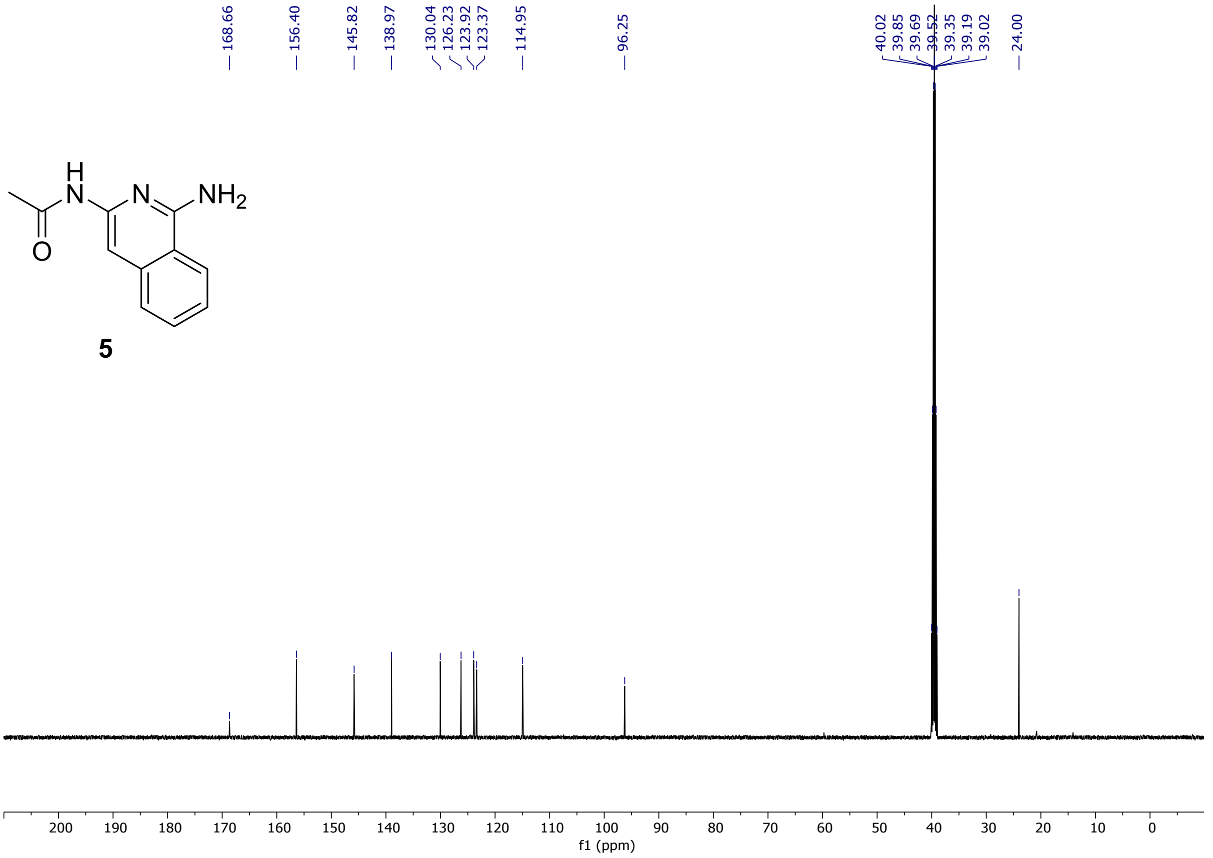

7. References

[1] Cox, J. M.; Elvidge, J. A.; Jones, D. E. H. Heterocyclic Imines Und Amines. Part X. 1,3-Diaminoisoquinoline, and the Fine Structures of Related Nitroso-Compounds and Pyridine Derivatives. *J. Chem. Soc.* **1964**, 1423–1430. https://doi.org/10.1039/jr9640001423.
